# Forced Disorder in the Solid Solution Li_3_P–Li_2_S: A New Class of Fully Reduced Solid Electrolytes for Lithium Metal Anodes

**DOI:** 10.1021/jacs.2c01913

**Published:** 2022-08-30

**Authors:** Conrad Szczuka, Bora Karasulu, Matthias F. Groh, Farheen N. Sayed, Timothy J. Sherman, Joshua D. Bocarsly, Sundeep Vema, Svetlana Menkin, Steffen P. Emge, Andrew J. Morris, Clare P. Grey

**Affiliations:** † Yusuf Hamied Department of Chemistry, University of Cambridge, Lensfield Road, Cambridge CB2 1EW, U.K.; ‡ Institute of Energy and Climate Research (IEK-9), Forschungszentrum Jülich GmbH, 52425 Jülich, Germany; § Institute of Physical Chemistry, RWTH Aachen University, 52056 Aachen, Germany; ∥ Department of Chemistry, University of Warwick, Gibbet Hill Road, Coventry CV4 7AL, U.K.; ⊥ The Faraday Institution, Quad One, Harwell Campus, Didcot OX11 0RA, U.K.; # School of Metallurgy and Materials, University of Birmingham, Birmingham B15 2TT, U.K.

## Abstract

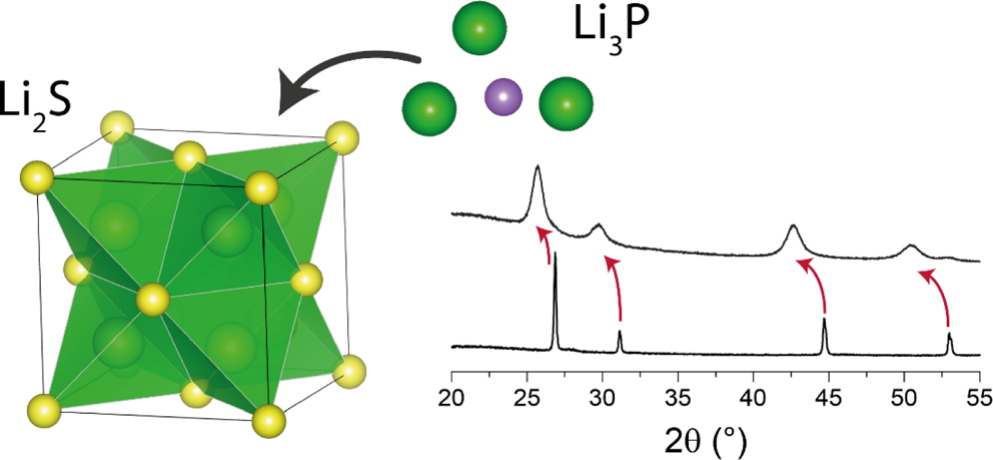

All-solid-state batteries based on non-combustible solid electrolytes are promising candidates for safe energy storage systems. In addition, they offer the opportunity to utilize metallic lithium as an anode. However, it has proven to be a challenge to design an electrolyte that combines high ionic conductivity and processability with thermodynamic stability toward lithium. Herein, we report a new highly conducting solid solution that offers a route to overcome these challenges. The Li–P–S ternary was first explored via a combination of high-throughput crystal structure predictions and solid-state synthesis (via ball milling) of the most promising compositions, specifically, phases within the Li_3_P–Li_2_S tie line. We systematically characterized the structural properties and Li-ion mobility of the resulting materials by X-ray and neutron diffraction, solid-state nuclear magnetic resonance spectroscopy (relaxometry), and electrochemical impedance spectroscopy. A Li_3_P–Li_2_S metastable solid solution was identified, with the phases adopting the fluorite (Li_2_S) structure with P substituting for S and the extra Li^+^ ions occupying the octahedral voids and contributing to the ionic transport. The analysis of the experimental data is supported by extensive quantum-chemical calculations of both structural stability, diffusivity, and activation barriers for Li^+^ transport. The new solid electrolytes show Li-ion conductivities in the range of established materials, while their composition guarantees thermodynamic stability toward lithium metal anodes.

## Introduction

Key goals for designing next-generation energy storage systems include improving energy density, safety, reliability, and cost. Lithium-ion batteries (LIBs) are among the most promising storage systems due to their high energy densities and voltages.^1^ However, a transition toward all-solid-state batteries (ASSBs) would not only allow the graphite to be replaced by pure or alloyed lithium anodes,^2−4^ leading to higher capacities, while also potentially removing the flammability issues associated with the non-aqueous electrolyte. ASSBs also outperform conventional LIBs with regard to high-temperature applications with reduced flammability and risk of thermal runaway.^5,6^ Moreover, ASSBs could, in principle, achieve faster (dis)charging than traditional LIBs due to the absence of bulk polarization effects since Li^+^ ions are the sole conductors of ionic charge.^7^ Nonetheless, commercialization of lithium ASSBs is still impeded by the absence of a highly conductive solid electrolyte (SE) that is (kinetically and/or thermodynamically) stable toward lithium metal and the cathode materials, while also accommodating the mechanical stresses that occur during battery cycling from volume changes in the active materials. In addition, dendrite-free plating of lithium metal remains a problem.

A wide range of promising SEs have emerged and attracted considerable and widespread interest.^8^ One class involves oxides such as cation-substituted Li_7_La_3_Zr_2_O_12_ (LLZO)-derived compounds, which feature high ionic conductivity, kinetic stability toward lithium metal, and can be handled in ambient atmosphere.^9^ However, due to their mechanical stiffness, they are prone to cracking, mechanical instability, and are not resilient toward lithium dendrite formation, particularly at high current densities.^7^

A second promising class of materials contains sulfide ions. Although moisture and air sensitivity make their use in practical applications challenging, their extremely high ionic conductivity and stress-accommodation has motivated activity world-wide to identify new materials.^10^ Their superior conductivity arises from the replacement of oxide anions with larger, more polarizable sulfide anions, leading to decreased Li-ion jump barriers.^11^ Extremely high ionic conductivities similar to those measured for liquid electrolytes were found in materials containing thiophosphate polyhedra as key building blocks. While initial work focused on glasses and crystalline phases within the pseudo-binary (Li_2_S)_*x*_–(P_2_S_5_)_1–*x*_,^12,13^ doped crystalline derivatives are among the most conductive SEs to date, including Li_10_GeP_2_S_12_ (LGPS, σ = 12 mS cm^–1^),^14^ Li_6–*x*_PS_5–*x*_ClBr_*x*_ (σ = 24 mS cm^–1^),^15^ and Li_9.54_Si_1.74_P_1.44_S_11.7_Cl_0.3_ (σ = 25 mS cm^–1^).^16^

More recently, lithium-rich ternary phosphides were introduced as SE candidates, with the phosphide anions P^3–^ possessing even larger polarizabilities than sulfide anions. These phases are based on anionic TtP_4_ tetrahedra with Tt = Al, Si, Ge, Ga, or Sn and show conductivities of up to 3 mS cm^–1^ for Li_9_AlP_4_ at room temperature.^17−23^ Their large numbers of charge carriers and low density make them attractive potential SEs, albeit being sensitive to oxygen and moisture.

Despite numerous advantages, the introduced SE materials suffer from a unifying drawback; they are inherently thermodynamically unstable toward lithium metal, as demonstrated for metal/metalloid-containing oxides, sulfides, and thiophosphates.^24−27^ For example, thiophosphates degrade to mixtures of lithium sulfides and phosphides through reduction of phosphorus ions from formal oxidation states of +5 to −3 in Li_3_P. The resulting lithium binaries (Li_2_O, Li_2_S, Li_3_P, and LiX with X = halogenide, etc.) are mediocre electrolytes at best and thus form a conductivity bottleneck at the SE–lithium metal interface. Furthermore, other involved cations might eventually lead to short circuiting due to reduction to lithium intermetallics. For example, Ge^4+^ present in LGPS^28^ and phosphidogermanates can result in the formation of electronically conductive germanides,^26,27^ which pose the risk of propagated decomposition and short circuits via the formation of mixed ionic-electronic conducting interphases.^28^

In searching for a highly Li^+^-ion conductive SE that is thermodynamically stable toward lithium metal, both the structural and thermodynamic characteristics and the method used to synthesize the material have to be considered. Many transport processes in solid-state materials are enabled by the presence of defects, the various processes being generally described by vacancy, interstitial, and collective mechanisms.^11,29^ Aside from introducing vacancies or interstitial carrier sites by doping aliovalent lattice ions, a synthetic approach that introduces a manifold of general defects, such as ball milling, may lead to inherently high conductive electrolytes for two reasons. First, amorphization and partial reorganization during subsequent annealing allows control of the desired level of crystallinity. Consequently, many sulfide SEs are synthesized via ball milling of Li_2_S and sulfides such as P_2_S_5_, B_2_S_3_, and GeS_2_, often followed by a subsequent heat treatment to increase crystallinity. Second, low-temperature approaches such as ball milling can also allow the formation of metastable electrolytes with superior ionic conductivity compared to their more ordered thermodynamically stable counterparts, as observed for the materials discussed in this paper. Phase formation can occur either directly during the milling process or at low subsequent annealing temperatures that allow only partial reorganization of the lattice ions.

Quantum chemical calculations, primarily employing density functional theory (DFT), have been commonly used to scrutinize structural, electronic, transport, and spectral properties of various Li-ion battery materials, particularly solid-state electrolytes.^30−32^ Moreover, DFT is often used jointly with crystal structure prediction (CSP) approaches, including stochastic (e.g., Ab Initio Random Structure Search, AIRSS), evolutionary [e.g., genetic algorithm (GA), and USPEX], and particle-swarm optimization (e.g., CALYPSO) methods in the in silico discovery of novel functional materials.^33,34^ Even though the latter tools were used to predict chemically-doped thiophosphide electrolytes [e.g., Li_3_Y(PS_4_)_2_^35^ and Li_*x*_(PS_4_)_*y*_X_*z*_ (X = Cl, Br, I),^36^ the pure Li–P–S phase diagram has so far remained largely uncharted, apart from individual studies focusing exclusively on a few Li_2_S–P_2_S_5_ phases.^37^

Here, we show that we can combine stability with high conductivity by introducing forced disorder into lithium (pseudo)-binaries: high-energy ball milling of mixtures of Li_3_P and Li_2_S leads to SEs that not only are thermodynamically stable toward lithium metal but also show Li-ion conductivities and activation energies in the range of established SEs. The structure and conduction mechanisms of these materials are studied by powder X-ray and neutron diffraction [XRD and PND], solid-state nuclear magnetic resonance (ssNMR) spectroscopy and relaxometry, and potentiostatic electrochemical impedance spectroscopy (PEIS). DFT calculations combined with structure search based on AIRSS and structure model configuration enumeration (CE) were first utilized to explore the Li–P–S phase diagram and then to validate proposed model structures and study material properties of the new Li–P–S phases.

## Experimental Section

All handling of the materials was conducted in an argon-filled UNIlab (M. Braun) [*p*(O_2_)/*p*^0^ < 1 ppm, *p*(H_2_O)/*p*^0^ < 1 ppm] or VAC glovebox [*p*(O_2_)/*p*^0^ < 5 ppm, *p*(H_2_O)/*p*^0^ < 5 ppm].

### Solid-State Synthesis of Li_3_P

A mixture of Li metal (136 mg, 19.7 mmol, 3 equiv; LTS Research, 99.95%) and *P*_red_ (204 mg, 6.60 mmol, 1 equiv; Sigma-Aldrich, ≥ 99.99%) was filled into a niobium crucible (2–4 cm high, ca. 10 mm in diameter) inside a quartz glass ampoule. After sealing the ampoule under dynamic vacuum, the reaction mixture was heated to 200 °C at a rate of 1 °C min^–1^, held at this temperature for 2 h, subsequently heated to 400 °C at 1 °C min^–1^, annealed for 2 h, and finally cooled to room temperature. After recovery in an Ar-filled glovebox and grinding, Li_3_P was obtained as a dark brown powder (purity: 95–99 wt %; impurity: LiP, Supporting Information Figure SA1). Li metal residues were occasionally found but could be easily separated from the product. For neutron diffraction, enriched ^7^Li metal (Sigma-Aldrich, ≥ 99.8%, ≥99.8 atom % ^7^Li) was used for the syntheses.

### Mechanochemical Synthesis of Ternary *x*Li_3_P-(1 – *x*)Li_2_S Solid Solutions

Typical amounts of approximately 250 mg of varying mixtures of Li_2_S (Sigma-Aldrich, 99.98%) and Li_3_P were ground together in an agate mortar and filled into a 15 mL ZrO_2_ ball mill jar containing 5 ZrO_2_ balls (10 mm diameter) and used in the Fritsch Pulverisette 23 mini mill operating at 50 Hz in an Ar-filled glovebox. The *x*Li_3_P-(1 – *x*)Li_2_S mixtures (*x* = 0.33–0.8) were ball milled in intervals of 10 min, interrupted by cooling phases of 10 min. Milling was performed until the Li_2_S signal in the powder X-ray diffractograms had disappeared, usually after 15–30 milling cycles (i.e., 2.5–5 h total milling time). The *x* = 0.67 sample was subsequently annealed at 300, 400, and 500 °C to check the thermal stability.

### X-ray Diffraction

The air-sensitive powder samples were finely ground in an agate mortar, filled into 0.3 mm diameter glass capillaries, and sealed with two-component glue. Diffraction data was collected at 296(2) K on the Panalytical Empyrean diffractometer equipped with a Ni filter using Cu *K*_α_ radiation (λ = 1.5406, 1.5443 Å). Rietveld refinement was performed using the TOPAS Academic software package (v. 4.1).^38^ Crystal structures were visualized using the Diamond and Vesta software package.^39^ Room temperature synchrotron powder XRD was performed at I11 beamline at the diamond light source (λ = 0.826 Å) for 5° ≤ 2θ ≤ 150° (Δ2θ = 0.004°).

### Neutron Diffraction

Room temperature PND experiments for structural characterization were performed on the Polaris diffractometer, at the ISIS Neutron and Muon Source, at the Rutherford Appleton Laboratory. Joint structural Rietveld refinements between the multi-bank time-of-flight (TOF) PND data and synchrotron powder XRD data (for *x* = 0.5) or laboratory powder XRD data (for *x* = 0.67) were performed with the TOPAS software package (v. 6),^38^ using a pseudo-voigt peak shape for the PND patterns and a full voigt peak shape for the XRD patterns. Atomic positions, occupancies, displacement parameters (*B*_iso_), and atomic parameters/weight percentage of a minor Li_2_O impurity phase were jointly refined for the neutron and XRD patterns, while the lattice parameters were refined independently.

### Solid-State NMR

Ball-milled mixtures of *x* Li_3_P and (1 – *x*) Li_2_S (*x* = 0.33–0.8) were ground in agate mortars and packed into 1.3 and 4.0 mm ZrO_2_ rotors with 2.5 and 50 μL internal volume, respectively. 4.0 mm rotors with ZrO_2_ top caps were used for non-ambient temperature experiments. They were packed with approximately 40 mg of sample using Teflon tape to position the sample at the center of the rotor so as to reduce temperature gradients within the sample volume.

^7^Li and ^31^P magic angle spinning (MAS) NMR experiments were performed at sample spinning speeds of 12.5 kHz (4 mm) and 50 kHz (1.3 mm) on a 16.4 T magnet with the Bruker AVANCE III console using Bruker 1.3 and 4 mm double/triple resonance MAS probes at varying temperatures between −70 and 125 °C. Low temperature experiments were performed by cooling the gas with a liquid N_2_ heat exchanger. The measured temperature at the outside of the spinning rotor was correlated to the sample temperature by measuring the chemical shift of an external KBr sample at identical spinning speeds and temperatures.^40^

The relaxometry measurements were conducted without prior annealing of the ball-milled samples. We observed an annealing effect on the line shape of the ^7^Li signal, decreasing the linewidth with time and repetition of heating and cooling. To assure consistent results, we repeatedly annealed the samples inside the NMR probe at 125 °C, followed by cooling to 30 °C, and did not start the NMR relaxometry experiments before the line shape was identical with the previous acquisition at an identical temperature. At very low temperatures, the exponential decay curves of the relaxation experiments in the rotating frame deviated from a simple mono-exponential decay, partly due to impurities with very long relaxation times. Stretched exponential functions provide reasonable fits; however, their physical meaning is debatable. These effects are well-known in the literature and have been treated similarly.^41^

^7^Li and ^31^P shifts were externally referenced using solid Li_2_CO_3_ (δ = 0.0 ppm;^42^ 99.999%, Aldrich) and NH_4_H_2_PO_4_ (δ = 0.8 ppm;^43^ 99.999%, Aldrich) as secondary chemical shift references. These compounds were used for pulse optimization as well. Unless stated otherwise, ^7^Li and ^31^P NMR signal line shapes were measured with rotor-synchronized Hahn echo pulse sequences. Bruker Topspin was used for raw data handling and processing.^44^

### Electrochemical Characterization

Powder samples were cold-pressed with a pressure of 1000 psi, obtaining pellets of 6.35 mm diameter and an approximate height of 1 mm. The pellets were sandwiched between two Li metal discs (3 mm diameter) and again between two stainless steel (SS) plates. The sandwich was moved to a hotplate, where a constant pressure of around 2 MPa was applied while heat-treated at 110 °C for 1 h. After 1 h, the pellet was flipped around to heat both sides evenly. The SS|Li|SE|Li|SS structure was then transferred into a Kapton film-lined swagelok cell. For Li_3_P (*x* = 1), and an additional pellet, sandwiched by gold blocking electrodes, was prepared for PEIS validation.

The PEIS measurements were performed with the Biologic VSP200 (and VSP300 for Li_11_P_3_S) instrument at a frequency range from 1 MHz (and 7 MHz for Li_11_P_3_S) to 1 Hz using 100 mV amplitude. For variable temperature (VT) PEIS, the swagelok cell was placed inside an oven and heated to 100 °C for 1.5 h, prior to measurement. The temperature was monitored using a thermocouple in direct contact with the Swagelok cell. Subsequently, the system was then cooled to 80, 60, 40 °C, and room temperature, and EIS spectra were recorded at each temperature after reaching equilibrium. Cooling of the samples below room temperature was achieved by blowing gas from a liquid nitrogen container into the chamber. PEIS measurements were taken at 0, −10, −20, −30, −40, and −50 °C, where possible. The spectra were recorded multiple times at each temperature to ensure that the equilibrium was reached.

### First-Principles Calculations

Plane-wave density-functional theory (DFT) electronic structure calculations were performed using the CASTEP code^45,46^ (v. 17.21), which is an implementation of periodic boundary conditions and the pseudopotential approximation. The generalized-gradient approximation (GGA) was used in the Perdew–Burke–Ernzerhof (PBE) exchange–correlation functional form.^47^ The atomic positions and lattice parameters were fully relaxed at this level of accuracy, using the LBFGS optimizer.^48,49^ Stochastic CSPs were performed with the Ab Initio Random Structure Search (AIRSS) method^50,51^ and with GA. These predicted structures were combined with those collected from various material databases and those obtained through prototyping via chemical substitutions. All the database management and phase diagram plotting tasks were done by the *MATADOR* package.^52^ All crystal structures were visualized with VESTA.^39^

Ab initio molecular dynamics (AIMD) simulations (*NVT* ensembles) were performed to estimate the Li-ion conductivity within the Li–P–S ternaries under investigation. For AIMD simulations, the Vienna Ab initio Simulation Package (VASP, v. 5.4.1) was used,^53−55^ while employing the projector augmented-wave (PAW) method jointly with GGA.

To create the models that represent two possible structural models for the Li_2_S–Li_3_P mixtures, we started from the anti-fluorite structure of Li_2_S obtained from ICSD (Collection code: 54396, *Fm*3̅*m*, *Z* = 4, containing 8 Li and 4 S sites). To reproduce the exact molar P/S ratios for different stoichiometries and prevent spurious interaction of the defect sites with their periodic images, two extended model systems were adopted as the starting structures, namely, the 2 × 2 × 2 conventional supercell (extended version of the ICSD structure, *Z* = 32) and the 3 × 3 × 3 primitive cell (*Z* = 27). Here, an extended 3 × 3 × 3 primitive cell is used, given the unpractical size of the 3 × 3 × 3 conventional supercell (*Z* = 108). One should note the large number of defect configurations to consider, resulting from the abundant S(4*a*) and interstitial (4*b*) sites in the original Li_2_S structures, wherein numerous substituents are placed. To overcome this limitation, a sequential doping approach was adopted to constrain the number of possibilities, similar to our previous report (rather than placing all defects at once).^56^ Details of this CE procedure and the system preparation can be found in Supporting Information, Section B2. Further technical details regarding the DFT calculations can be found in the Supporting Information Section B1.

Solid-state NMR parameters were obtained with the gauge-including PAW (GIPAW) approach.^57,58^ Predicted isotropic shielding values from the DFT/GIPAW calculations were corrected using the reference shift values of σ_ref_ = 89.0 and 300.0 ppm for obtaining the ^7^Li and ^31^P chemical shifts, respectively. These reference shift values were determined using the procedure detailed in Supporting Information, Section B1. A Lorentzian broadening of 2 and 7.5 ppm was used for the simulations of the ^7^Li and ^31^P spectra, respectively.

## Results and Discussion

### Exploring New Li–P–S Phases Using Stochastic Structure Searches

Motivated by the promising SE properties of sulfide-containing materials in general, we first performed a systematic search of the Li–P–S system using stochastic structure searches, to guide our exploratory synthesis. Plotting the ternary Li–P–S phase diagram comprising all known structures (Li_7_P_3_S_11_, LiPS_3_, Li_2_PS_3_, and Li_3_PS_4_, Supporting Information Figure SB2) reveals that all phases lie on (or close to) the pseudo-binary line between Li_2_S and P_2_S_5_. In contrast, the other tie-lines formed by Li_2_S and other stable P–S and Li–P binaries remain hitherto uncharted. With this in mind, we performed high-throughput stochastic DFT searches along these predefined tie-lines, using AIRSS, GA, and element substitutions, and generate over 25,000 crystal structures.

**Figure 1:**
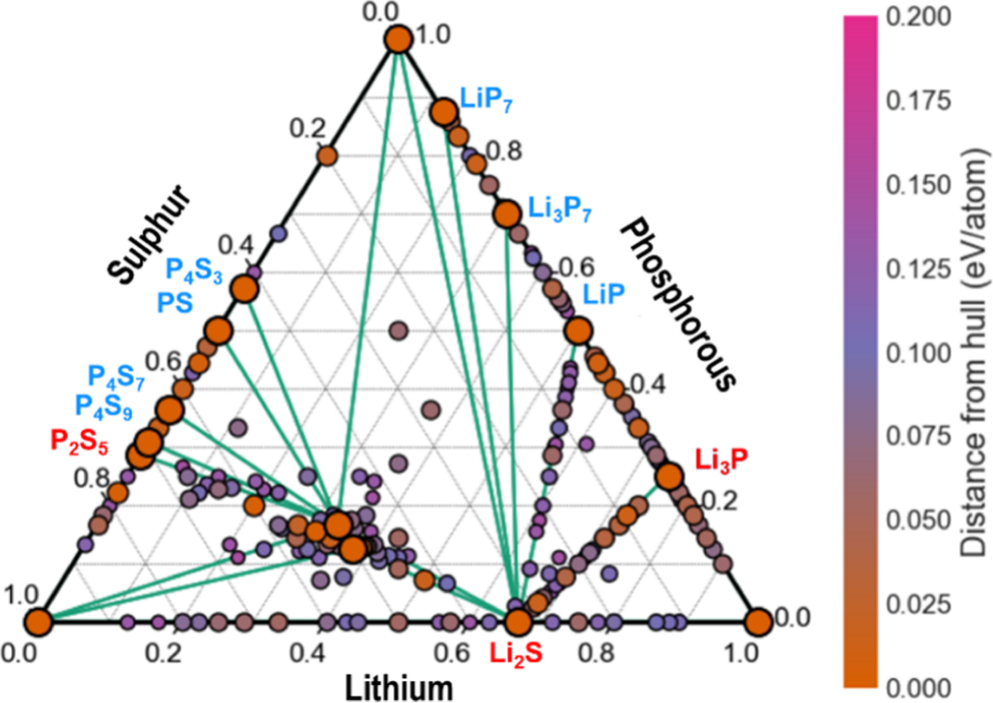
Li–P–S ternary phase diagram as computed using DFT/PBE level stochastic structure searches. The corners of the diagram correspond to the constituent pure Li/P/S phases and the edges to their binary phases. The larger the circle, the closer a given structure is to the hull. Binary phases that are stable (on the hull) are labeled. Only structures within 150 meV/atom of the hull are shown for clarity. Individual plots of these tie-lines (Li_2_S–P_2_S_5_, Li_2_S–Li_3_P, and Li_2_S–LiP pseudo binary diagrams) are given in Supporting Information Figure SB3.

The resulting phase diagram (Figure 1) shows that all new phases with low formation energies are exclusively located on the Li_2_S–P_2_S_5_, Li_2_S–Li_3_P, and Li_2_S–LiP tie-lines. In particular, our structure predictions discovered new metastable Li_2_S–P_2_S_5_ ternaries, viz. Li_5_P_3_S_10_ and Li_5_PS_5_ located 31.2 and 54.4 meV/atom above the Li–P–S hull, respectively (Table 1). Albeit being thermodynamically accessible, these two Li_2_S–P_2_S_5_ mixture ternaries are likely to decompose into the nearby stable Li_3_PS_4_ and Li_2_PS_3_ ternaries. e.g., Li_4_P_2_S_7_ has been shown to decompose to form Li_2_PS_3_ and sulphur (i.e. Li_4_P_2_S_7_ → 2Li_2_PS_3_ + S).^12^

**Table 1:** Ground-State Structures of Selected Known Phases and New Ones Discovered in the Stochastic Searches and Their Predicted Hull Distances (Δ*E*, meV/Atom, with Literature Values in Brackets) and Activation Energies (*E*_a_, meV) for Li-Ion Diffusion and Diffusivities (*D*^a^, cm^2^ s^–1^) at RT from AIMD simulations^a^

phase	Δ*E*	space group	*E*_a_^a^	*D*^a^ at RT
Li_2_S	0.0	*Fm*3̅*m*	800	1.1 × 10^–9^
			750^b^	
Li_3_P	0.0	P63/*mmc*	696	3.6 × 10^–9^
			530^c^	
Li_2_S–P_2_S_5_ Tie-Line				
Li_4_P_2_S_7_ (known)	0.9	*P*1̅	249	7.9 × 10^–6^
			300^d^	
Li_7_P_3_S_11_ (known)	16.2	*P*1̅	160	1.6 × 10^–5^
			187^e^	
Li_5_P_3_S_10_	31.2	*C*2/*c*	670	1.1 × 10^–8^
Li_5_PS_5_	54.4	*Pbcm*	280	2.4 × 10^–7^
Li_2_S–Li_3_P Tie-Line				
Li_8_P_2_S	12.4	*P*1	232	6.5 × 10^–6^
Li_21_PS_9_	16.8	*P*1	325	7.6 × 10^–8^
Li_13_P_3_S_2_	38.2	*P*1	199	3.5 × 10^–6^
Li_5_PS	38.5	*P*4̅21*m*	190	4.4 × 10^–6^
Li_9_PS_3_	43.9	*P*42*nm*	267	8.2 × 10^–7^
Li_11_P_3_S	45.5	*P*21	250	2.2 × 10^–6^
Li_7_PS_2_	76.3	*Cm*	193	5.2 × 10^–6^
Li_12_P_2_S_3_	82.4	*P*21/*m*	246	4.5 × 10^–6^
Li_2_S–LiP Tie-Line				
Li_4_P_2_S	59.9	*Cm*	`	1.0 × 10^–9^
Li_6_P_4_S	87.7	*Cm*	596	4.3 × 10^–8^
Li_3_PS	128.0	*Cm*	222	6.3 × 10^–6^

a Structures are visualized in Supporting Information Figures SB4 and SB5. Activation energies (*E*_a_) for Li^+^ diffusion were estimated from Arrhenius type plots of diffusivity against temperature (Supporting Information Figure SB6) and where possible compared with previous experimental data.

b From ref (61).

c From ref (62).

d From ref (63).

e From refs (64) and (65).

More intriguing was the prediction of a series of new low-lying metastable phases on the Li_2_S–Li_3_P and Li_2_S–LiP tie-lines, with hull distances as small as 12.4 meV/atom (Li_8_P_2_S, Table 1). The absence of any stable phases on both tie-lines again suggests that these metastable phases are again likely to decompose into their respective parent materials at higher temperatures; they may nevertheless be synthesizable using low-temperature methods.

Besides having a low formation energy (i.e., thermodynamic stability), high ionic conductivity is a determining figure of merit for an electrolyte candidate. Ionic conductivity can be assessed by using AIMD simulations, by tracking the mobility of Li ions within the lattice, along a MD trajectory (Supporting Information, Section B1). The activation energy for Li transport within an electrolyte can then be estimated, assuming an Arrhenius behavior, by comparing diffusivities at varying temperatures.

**Figure 2:**
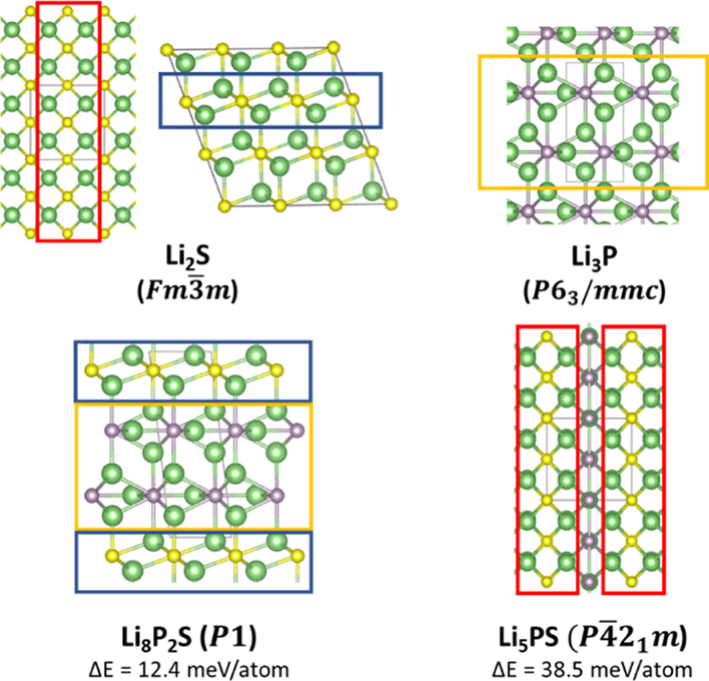
Ground state structures Li_2_S and Li_3_P and the newly predicted Li_8_P_2_S and Li_5_PS ternary phases, illustrating the structural motifs (shown as red, blue, and yellow rectangles) that are common to these phases. For Li_2_S, two different viewing angles of the same crystal structure are shown. The hull distances (Δ*E* values) of the two ternaries are also shown. Li, P, and S atoms are depicted by green, purple, and yellow balls, respectively.

Estimated activation energies for the newly found ternary phases from different tie-lines (Table 1) reveal that the Li_2_S–P_2_S_5_ ternary phases will require higher temperatures to achieve a good conductivity. By contrast, a few particular Li_2_S–Li_3_P ternaries are very promising as they display low activation energies (*E*_a_ = 190–230 meV), approaching that of the highest conducting phase within the Li–P–S ternary, Li_7_P_3_S_11_ (with predicted and experimental *E*_a_s of 160 meV and 120^59^ and 170^60^ meV, respectively). Considering the calculated high activation energies for the other members of the Li_2_S–P_2_S_5_ and Li_2_S–LiP ternaries and their likelihood to decompose into more stable ternary phases, we focused on the Li_2_S–Li_3_P tie-line. This tie-line contains many low-lying metastable phases with high room-temperature (RT) Li^+^ conductivity and low activation energies (e.g., Li_8_P_2_S; Tables 1 and SB3 and Supporting Information Figure SB6). A closer examination of phases identified in the initial search in this latter tie-line revealed that some structural motifs from the parent materials (Li_2_S and Li_3_P) are common in the predicted ground-state structures of some new ternaries. This is demonstrated for the Li_8_P_2_S and Li_5_PS cases, which are formed as the 1:2 and 1:1 mixture of Li_2_S/Li_3_P, respectively (Figure 2). This suggests that intergrowth between the two end member phases may be possible.

### Mechanochemical Synthesis

**Figure 3:**
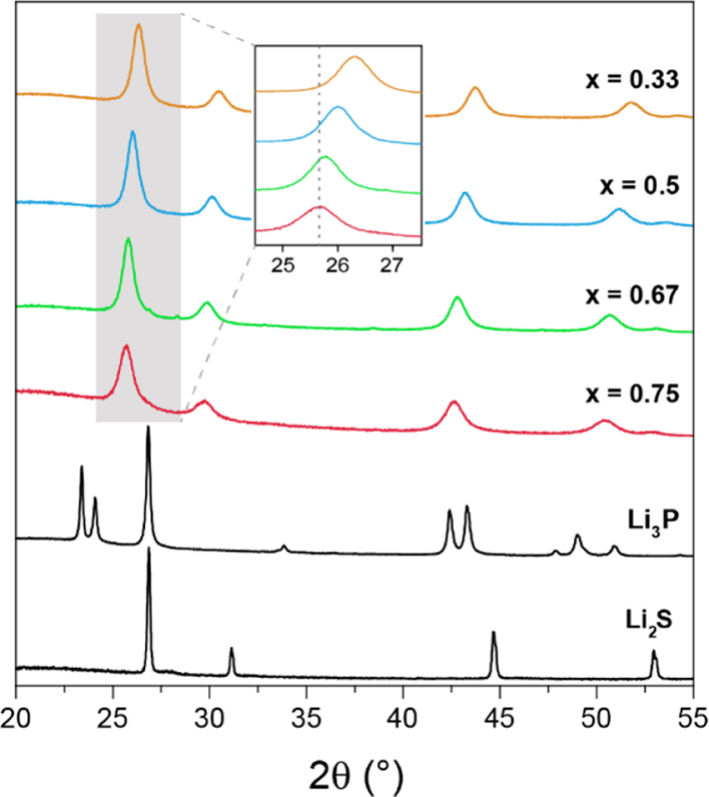
Laboratory powder X-ray diffractograms of the solid solutions in the system *x*Li_3_P–(1 – *x*)Li_2_S.

Motivated by the promising results obtained in the stochastic structure prediction searches, especially in the Li_2_S–Li_3_P pseudo-binary system, we employed different ball milling approaches to synthesize new compounds on this tie line. Solid solutions of *x* Li_3_P and (1 – *x*) Li_2_S were obtained by high-energy ball milling carried out inside an argon-filled glovebox until no Li_2_S reflections could be detected in the respective X-ray powder diffractograms of the samples periodically extracted from the ball-mill. Depending on the stoichiometry, 15–30 milling cycles of 10 min were necessary. The resulting solids feature a gradual color change (Supporting Information Figure SA2) from yellow to red with increasing amount of Li_3_P (brown), indicating a decrease of the optical band gap. The diffractograms of the solid solutions can all be indexed with a structure model based on the Li_2_S anti-fluorite crystal structure with a cubic close packing (ccp) of sulfide anions (Figure 3). Due to substitution of S^2–^ with the larger P^3–^ anion, higher amounts of Li_3_P led to gradually increasing cubic lattice parameters of 5.83(8), 5.90(6), 5.96(3), and 5.98(6) Å for *x* = 0.33, 0.50, 0.67, and 0.75, respectively. The changes in lattice parameters follow Vegard’s law, despite the different crystal structures of the two binaries at ambient pressure and temperature (Supporting Information Figure SA3). Structural compatibility can be understood by considering the hexagonal-to-cubic phase transition of Li_3_P at pressures around 4 GPa,^66,67^ a phase transition that might also be induced mechanochemically. The resulting Li_3_Bi-type structure resembles the anti-fluorite structure, but with filled octahedral voids. Additionally, known lithium phosphide ternaries with group 13 and 14 elements are similarly based on a ccp phosphide sublattice with high-valent cations situated in tetrahedral voids.^17−23,66^ As the high pressure cubic Li_3_P phase transforms rapidly into the hexagonal phase when pressure is removed, it would, however, be difficult to observe via our experimental approach.

A stability range of the *x* Li_3_P and (1 – *x*) Li_2_S anti-fluorite solid solution of approximately 0.33 < *x* ≤ 0.75 was established. The lower boundary is determined by the milling time, longer milling times being required for a complete reaction, suggesting a very weak driving force for solid solution behavior under the ball-milling conditions. The higher boundary is determined by the stability of the solid solution phase once formed: samples with *x* ≥ 0.8 decomposed quickly to form Li_3_P and a phosphide-poorer solid solution, the samples changing color within minutes after the end of the milling process from black to brown, the latter resembling the color of the starting mixture prior to milling. This color-change behavior can be reversed by re-milling. The other solid solutions with 0.33 < *x* ≤ 0.75 are stable at room temperatures and elevated temperatures up to at least 125 °C under argon. Upon annealing at even higher temperatures (e.g., between 400 and 500 °C for *x* = 0.67), the solid solutions decompose into the respective binaries, Li_3_P and Li_2_S, indicating that the ball-milled mixtures are metastable at these temperatures (Supporting Information Figure SA5). Attempts to prepare the crystalline solid solutions by annealing the binaries or pure elements were unsuccessful, further supporting the assessment that these phases are metastable.

**Figure 4:**
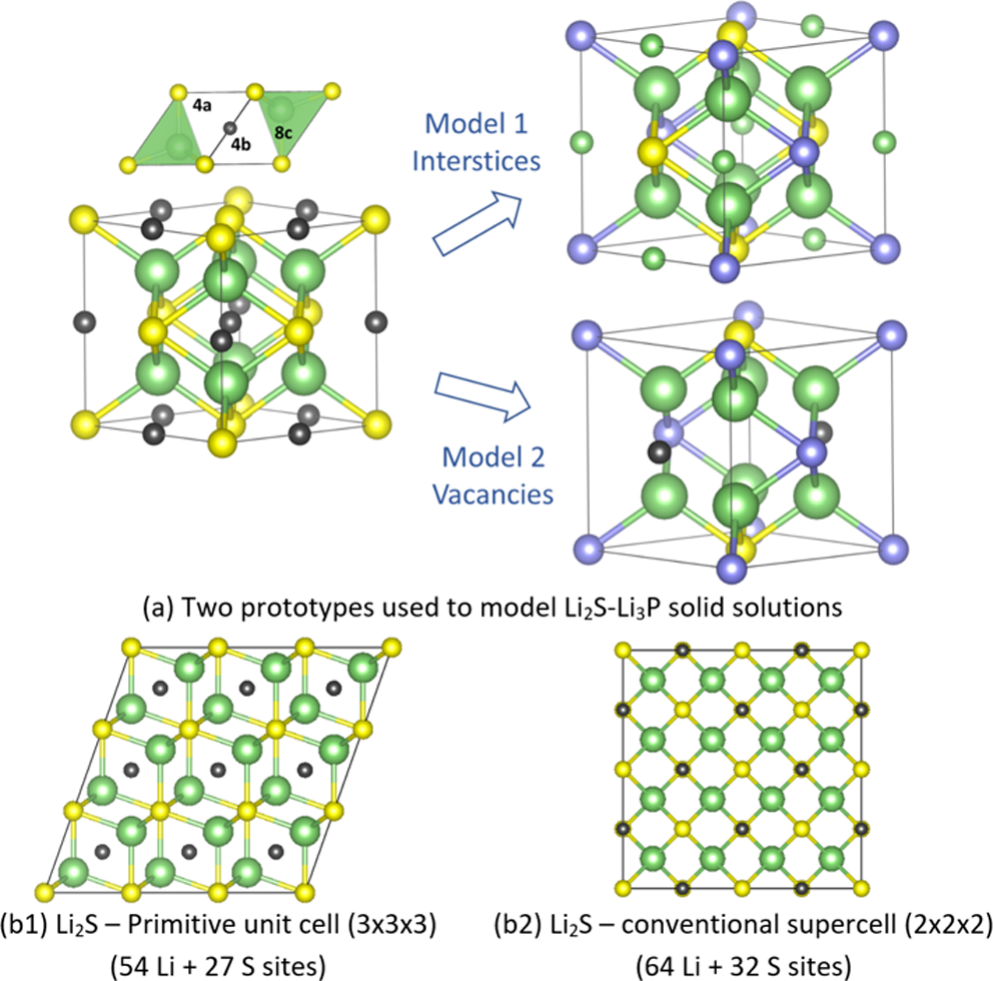
(a) Two possible structural models for the Li_2_S–Li_3_P solid solutions; (b) Li_2_S supercells used as starting points for the DFT analysis on defect configurations. Color code: Li: green, S: yellow, P: purple, and interstices: black. The two models involve P^3–^ random substitution on the S^2–^ (4a) sublattice; in Model 1, Li is inserted in the octahedral vacancies found on the edges of the Li_2_S cubic unit cell and in Model 2, vacancies are created on the S (4a) sublattice. The initially vacant Li_2_S interstitial (octahedral) sites and the vacancies created in the anion sublattice (in Model 2) are depicted by small black spheres in the Li_2_S structures (a, left-hand side). The extra Li atoms added on the interstices (in Model 1) are then indicated with small green balls.

The measured XRD pattern is consistent with two possible prototype crystal structure models (Figure 4a): in Model 1, the anti-fluorite type-derived structure has P^3–^/S^2–^ occupational disorder on the anion position (Wyckoff 4*a* sites) with additional *x* lithium cations being accommodated in the octahedral voids, Li_2+*x*_(P_*x*_S_1–*x*_) (i.e., for *x* = 0.67: “Li_2+2/3_(P_2/3_S_1/3_)_1_” or “Li_8_P_2_S”), while in Model 2, the anti-fluorite type structure maintains a stoichiometric cation sub-lattice but now has vacancies and P^3–^/S^2–^ occupational disorder on the anion lattice. Model 2 can be represented as Li_2_(P_2/3x_□_1/3x_S_1–x_) (e.g., “Li_8_P_2_S_1_□_1_” for *x* = 0.67), and additional Li sites are unnecessary as the charge neutrality remains intact. Intermediates between these two extremes can also be envisaged.

### Structural Determination Using Powder Neutron Diffraction

Since it is difficult to use standard XRD to distinguish between these structures, given the very broad XRD patterns, the similar X-ray scattering factors of the S and P ions, and the small Li scattering factor for Li atoms, powder neutron diffraction was performed for two ^7^Li-enriched samples. NPD data for *x* = 0.5 (Li_5_PS) and *x* = 0.67 (Li_8_P_2_S) was co-refined with either synchrotron (*x* = 0.5) or laboratory (*x* = 0.67) XRD data. The investigated samples each contain about 2.5 wt % Li_2_O, visible in both the neutron and synchrotron patterns. This impurity was introduced during the preparation of the ^7^Li-enriched Li_3_P. Besides the Li_2_O and the *V* peak from the PND sample holder, the PND data shows no additional peaks apart from the anti-fluorite structure, confirming the *Fm*3̅*m* symmetry.

**Figure 5:**
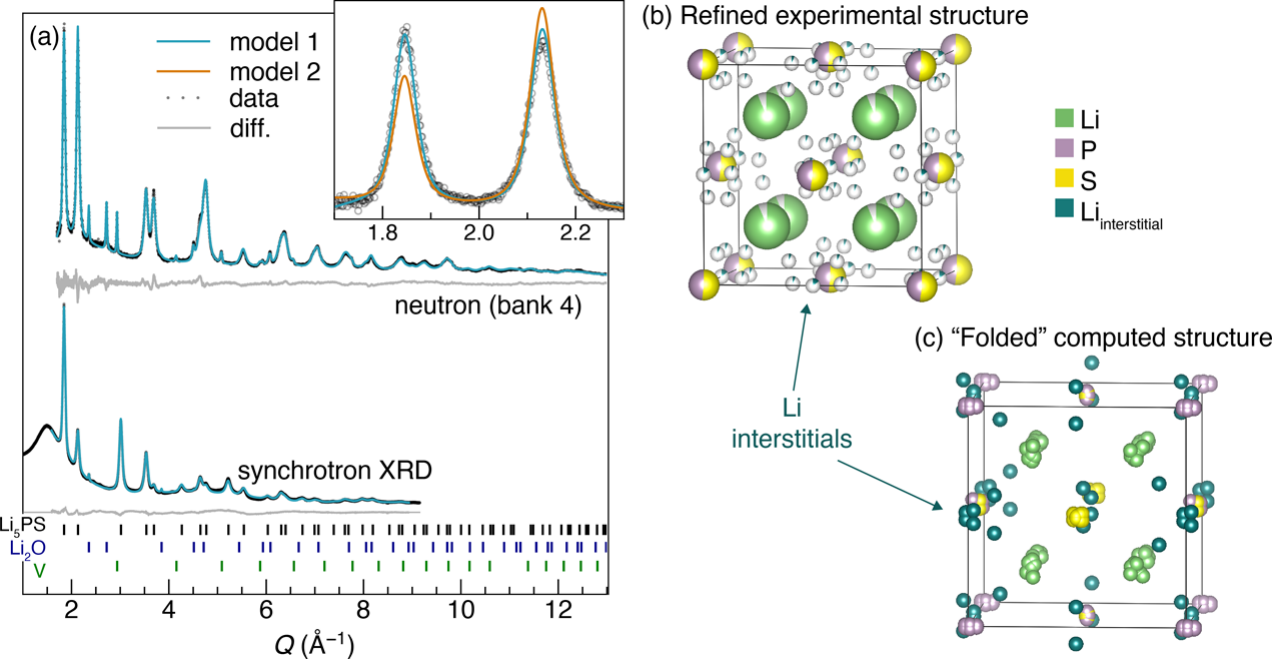
Combined synchrotron XRD and neutron powder diffraction analysis of the *x* = 0.5 sample (Li_5_PS). (a) Rietveld co-refinement of the synchrotron and neutron diffraction patterns with the structural model shown in (b), which corresponds to Model 1. For clarity, only a single bank (bank 4, 2θ = 92.6°) of the multi-bank neutron diffraction is shown, but the fitted data from the other banks can be found in Figure SA6. The inset of (a) shows a zoomed-in comparison of fits of Model 1 and Model to the neutron data, Model 1 giving a much better fit to the data (*R*_wp_ = 1.61 versus 2.34). (c) Relaxed DFT computed structure for *x* = 0.5, folded into the single conventional anti-fluorite structure cell.

Figure 5a,b shows the results of the co-refinement for the *x* = 0.5 sample. As seen in the inset of panel (a), attempts to refine using Model 2 gave poor fits to the neutron data, while fits based on Model 1 successfully modeled both the neutron and synchrotron XRD patterns. Initially, the fit to Model 1 was performed by introducing a Li interstitial site at the center of the octahedral void in the 4*b* Wykoff site. This resulted in satisfactory fits, but very large thermal parameters (*B*_eq_ ∼ 30) for lithium on the interstitial site, indicating that lithium is highly disordered within the octahedral void. In order to better account for the disordered interstitial lithium, a second off-centered lithium position within the octahedral void at the 48*i* Wyckoff site (identified by inspection of the neutron diffraction Fourier difference map) was introduced, resulting in the structure shown in Figure 5b. The *B*_iso_ of both interstitial positions (4*b* and 48*i*) was fixed to 1, and the *x* coordinate of the 48*i* site was refined. This model yielded an improved fit, and allowed for stable refinement of the Li site occupancies for all three lithium sites and the P/S ratio of the anion site, resulting in a refined composition of Li_5.09(2)_P_1.05(3)_S_0.95(3)_, in good agreement with the expected composition Li_5_PS (i.e., *x* = 0.5). The tetrahedral lithium site is found to be nearly fully occupied (94%), while the remaining lithium is distributed between centered (∼19% of interstitial Li) and off-centered (∼81% of interstitial Li) positions within the octahedral void. Similar analysis for the *x* = 0.67 sample (Supporting Information Figure SA6) also supports Model 1 and yields a refined composition of Li_8.69(3)_P_2.08(8)_S_0.93(8)_ and a corrected *x* value for the crystalline phase of *x*′ = 0.69. The refinement details and refined atomic positions, occupancies, and atomic displacement parameters are given in Supporting Information Tables SA1 and SA2.

Finally, the reflections observed in both the XRD and NPD patterns are broad, which could, in principle, be ascribed to a small crystallite size or high strain generated by the high-energy milling. The scanning electron microscopy images (Supporting Information Figure SA4) reveal that the powders consist of 0.5–1 μm fused secondary particles, formed from smaller primary particles, some as small as 200 nm. Attempts to record high magnification images were not successful and led to severe beam damage of the particles. Fits to the diffraction data assuming that small particle size led to peak broadening resulted in estimates for the particle size of only approximately 20 nm; given that this size is an order of magnitude less than the primary particle size, it should instead be interpreted as a measure of the coherence length (of the ordering). The fit is only slightly worse if only microstrain broadening is considered (caused by e.g., dislocations, grain boundaries, macroscopic variation in the composition, etc.). Thus, it is clear that these antifluorites contain considerable disorder. The presence of small quantity of amorphous/glassy phases, as seen in the Li_2_S–P_2_S_5_ tie line following ball-milling,^68,69^ cannot be readily ruled out using XRD or NPD. However, we note that no clear diffuse scattering (indicating local structure within a disordered/amorphous phase) is visible: the broad peak at 1.5 Å^–1^ is due to the borosilicate capillary but given the breadth of the Bragg reflections, further analysis is difficult. However, the composition of the crystalline phases, as determined by neutron diffraction, matches the starting stoichiometry, and thus, any amorphous or glassy phase must also have a similar stoichiometry.

### Local Structure Investigations by ^7^Li and ^31^P NMR

**Figure 6:**
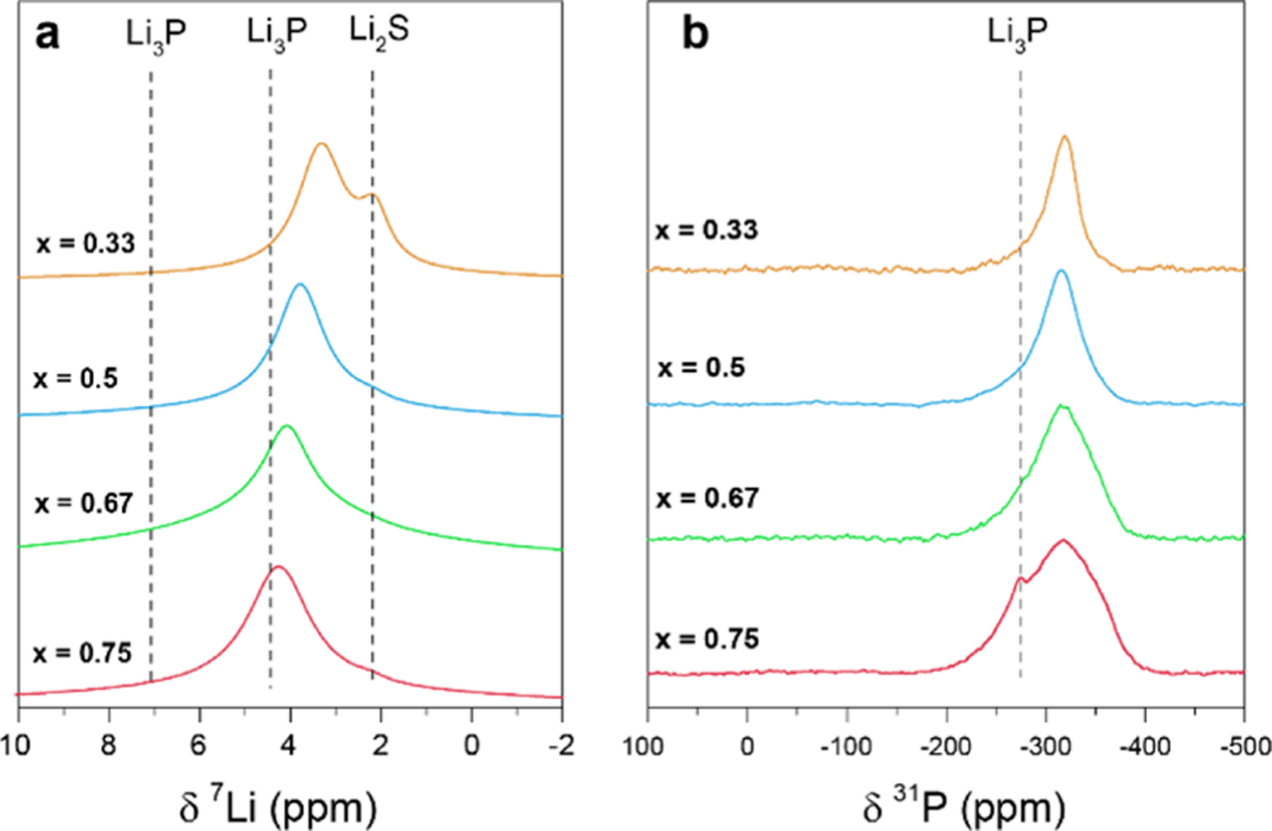
Room temperature (a) ^7^Li and (b) ^31^P MAS NMR spectra, at a 50 kHz spinning speed, of the solid solutions in the system *x*Li_3_P–(1 – *x*)Li_2_S.

The local structure of the solid solutions was investigated by ^31^P and ^6/7^Li solid state MAS NMR spectroscopy (Figure 6). The ^7^Li NMR spectra of the pure binaries contain resonances at 2.2 ppm for Li_2_S and 4.5 and 7.1 ppm for Li_3_P. The formation of the single-phase solid solution results in only one signal in between these two extrema. The exact shift is governed by the Li_2_S to Li_3_P ratio, further supporting the interpretation that they are continuous solid solutions of the Li_2_S structure type (Figure 6a). For *x* = 0.33, ^7^Li NMR revealed a significant amount of excess Li_2_S that was invisible in the PXRD patterns due to extreme peak broadening. This confirms that the (kinetic) limit of the solid solution range is *x* > 0.33 but also shows the value in using solid state NMR to investigate ball-milled samples in general to check for phase-purity (since minor components of Li_2_S are present in all the samples). ^7^Li NMR spectra after 16 and 30 milling cycles were virtually identical, suggesting that *x* = 0.33 exceeded the lower limit of the solid solution for finite milling times. Quantitative evaluation of the Li_2_S signals yields Li_2_S contributions of 13.0, 1.9, 1.5, and 1.0 ± 0.2% for samples with compositions corresponding to *x* = 0.33, 0.50, 0.67, and 0.75 (Supporting Information Figure SA7). From this (and the results from the ^31^P NMR spectra below) apparent solid solution compositions of *x*′ = 0.39, 0.51, 0.68, and 0.75 can be calculated, the slightly higher values of *x*′ for the antifluorite component than the original stoichiometry of the Li_2_S-rich members agreeing with the neutron diffraction results.

The ^7^Li spectra of thermally decomposed solid solutions of *x* = 0.67 (see XRD in Supporting Information Figure SA5) resemble those predicted for a simple mixture of the starting materials without any additional signals, consistent with the metastability of the solid solutions.

Both proposed structural models will generate several different local environments for the probed ^7^Li nuclei. In Model 1, filling both tetrahedral and octahedral voids of the anti-fluorite structure type should lead to two signals instead of only one (i.e., the pure Li_2_S case). Furthermore, the substitution of sulphur ions by phosphorus ions in the anion sublattice results in a series of different local environments that differ in the number of nearest-neighbor phosphorus atoms, leading, in principle, to several signals. Thus, the occurrence of only one rather narrow Li signal at room temperature suggests fast Li ion exchange between the different Li^+^ local environments. At lower temperatures, ionic motion is reduced and if sufficiently slow, it should be possible to resolve NMR signals with different chemical shifts arising from different local environments. Due to its small quadrupolar moment and lower natural abundance (reducing homonuclear coupling), ^6^Li NMR can be exploited to enhance spectral resolution. A ^6^Li NMR spectrum at −50 °C of the *x* = 0.50 sample reveals more than one signal (Supporting Information Figure SA8). A three-component fit provides a good fit to the spectrum, with the peaks being tentatively ascribed to tetrahedrally and octahedrally coordinated sites plus the Li_2_S residue, based on the agreement of the integral ratio and typical chemical shift ranges.

In contrast to the lithium spectra, ^31^P NMR shows a similar shift for all solid solutions (−317 ppm) at more negative ppm values compared to pure Li_3_P at −275 ppm (Figure 6b). The same chemical shift has been recorded for the isolated P^3–^ ion in Li_14_SiP_6_,^17^ a compound where the P^3–^ ion is surrounded by eight Li ions in a cubic arrangement, resembling the coordination sphere of the anion position in the anti-fluorite structure type proposed here. Residues of Li_3_P were found for the *x* = 0.75 sample with an estimated intensity contribution of 2.7% (Supporting Information Figure SA7). The apparent solid solution composition is invariant due to compensation with Li_2_S residues. A correlation between the ratio of the starting binaries and the linewidth was observed; a broader peak is seen as the phosphorus content increases. As the number of milling cycles among the different compositions was largely randomly distributed, we exclude line broadening effects due to milling. Thus, the linewidth characterizes an intrinsic property of the solid solution, the increase being ascribed to more diverse phosphorus environments (e.g., additional Li ions and second shell effects) with increasing Li_3_P amount; this is also supported by the change in the line shape from more Lorentzian to Gaussian. The shifts are explored further below via GIPAW (DFT) calculations. Finally, while both the ^6,7^Li and ^31^P NMR resonances are broad, no discrete peaks are seen, indicating the presence of very different local environments beyond those found in the solid solution and the end member phases.

### Measurements of Li^+^ Motion by NMR Relaxometry

**Figure 7:**
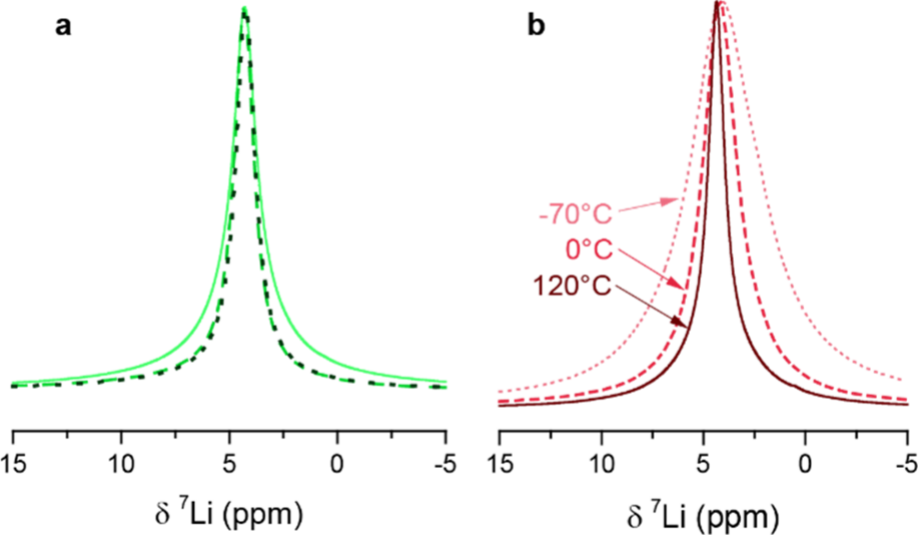
^7^Li MAS NMR spectra of (Li_3_P)_*x*_(Li_2_S)_1-*x*_ at a 12.5 kHz spinning speed. (a) Room temperature spectra of the *x* = 0.67 sample directly after ball-milling (full line), after annealing at 125 °C for 1 (dashed) and 2 h (dotted). (b) Temperature dependence of the NMR signal of the *x* = 0.75 sample. Spectra were recorded after annealing the sample at 125 °C.

Prior to the VT measurements, we first evaluated the stability of the samples above room temperature. Upon heating the samples to 125 °C within the spectrometer, the ^7^Li NMR linewidth of the signal decreased. The linewidth continued to decrease as the sample was held at this temperature for approx. 1 h (Figure 7a), likely due to sintering of the sample and annealing out of some of the defects. Additional cooling and heating of the samples did not result in additional irreversible line-narrowing, and no additional signals from, for example, Li_2_S were observed. Thus, for consistency, every sample was heated for 1 h before performing more detailed analysis on the effect of temperature on linewidths and relaxation times. As expected for a mobile system, the linewidth of the ^7^Li signals of the solid solutions decreased reversibly with increasing temperature (Figure 7b).

**Figure 8:**
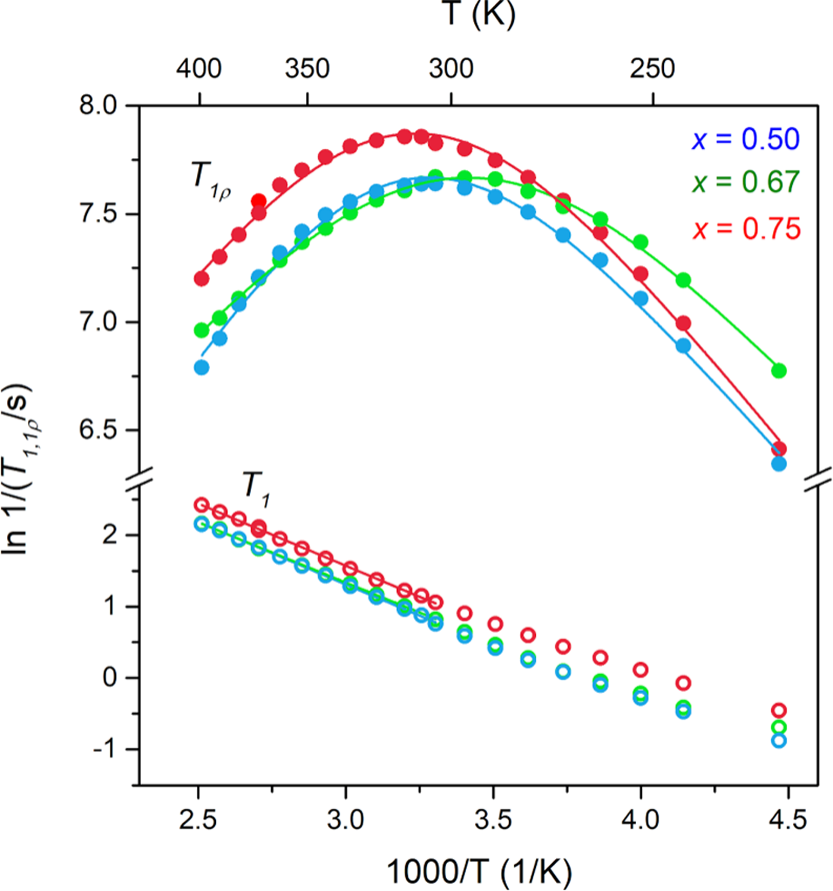
^7^Li relaxometry of *x*Li_3_P-(1 – *x*)Li_2_S in the temperature range from −50 to 125 °C. Linear (*T*_1_) and BPP-type (*T*_1ρ_) fits (solid lines) allow the extraction of activation energies summarized in Table 2.

In order to probe the local Li ion mobility of the solid solutions, ^7^Li NMR relaxometry measurements were performed between −50 and 125 °C. Spin lattice relaxation times in both the laboratory (*T*_1_ = 1/*R*_1_) and the rotating frame (*T*_1ρ_ = 1/*R*_1ρ_) were measured, which are sensitive to the motion of the observed nuclei on the ns and μs time scale, respectively. The motion-induced fluctuations of dipolar and quadrupolar fields result in a minimum of the relaxation time for a given temperature.^70^ At the minimum, the correlation rate (τ_C_^–1^) and Larmor frequency ω_0_ (270 MHz for ^7^Li at 16.4 T; for *T*_1_) or frequency in the locking field ω_1_ (for *T*_1ρ_ measurements) are approximately equal τ_C_^–1^ ≈ ω_0/1_. Above and below this point, the correlation time is proportional to the relaxation time and shows Arrhenius behavior for uncorrelated three-dimensional motion, allowing the activation energies of the motion to be extracted (Figure 8 and Table 2). For solid-state systems, the relaxation behavior can also exhibit dimensionality and correlation effects of the motion, leading to different apparent activation energies for the high and low temperature sides of the relaxation curves.^71^

**Table 2:** Activation Energies (*E*_a_ in eV) from NMR Relaxometry (*E*_a,T1_^LT^, *E*_a,T1ρ_) Compared with Ea^EIS^, Obtained from Arrhenius Plots of VT (−50 to 100 °C) EIS Data for xLi_3_P-(1 – *x*)Li_2_S Samples^a^

*x*	*E*_a,T1_^LT^	*E*_a,T1ρ_	*E*_a_^EIS^
0.33			0.20
0.5	0.15	0.18	0.17
0.67	0.15	0.13	0.15
0.75	0.15	0.15	0.16
1.00			0.19

a LT denotes the low temperature flank with respect to τ_C ×_ ω_0_ ≈ 1 in the NMR relaxometry plot (Figure 11).

Samples with *x* = 0.50, 0.67, and 0.75 were investigated, and similar activation energies were extracted from both the *T*_1_ and *T*_1ρ_ measurements. The *T*_1_ measurements yielded only the region for τ_C_ ω_0_ ≫ 1. A plot of the natural log of the spin–lattice relaxation rate (*R*_1_) against the reciprocal temperature yields the low temperature flank. The extracted activation energy of *E*_a,T1_^LT^ = 0.15 eV for the fastest probed motion is among the lowest values that have been determined for common SEs.^20,70,72^ Higher measurement temperatures to record the entire parabola and particularly the maximum were avoided due to possible decomposition of the metastable solid solutions. At temperatures below room temperature, the observed relaxation behavior deviates significantly from a simple exponential dependence proposed by the Purcell–Pound theory (BPP theory),^73^ resulting from additional effects seen in solids such as lattice vibrations or coupling with paramagnetic impurities.^74^

Maxima of the temperature-dependent relaxation rates were observed for all samples when measuring the relaxation times in the rotating frame, corresponding to a timescale in the order of 31 kHz [= ω_1_(^7^Li) in the locking field *B*_1_]. The well-defined maxima indicate a homogenous structure with defined diffusion pathways, in contrast to other mechanochemically synthesized SEs that can feature broad maxima extending over up to 100 °C.^72,75^ Due to the resolved maxima, a BPP-type *T*_1ρ_ relaxation expression, which is dependent on the spectral densities at 2ω_1_, ω_0_, and 2ω_0_ (see Supporting Information),^71,76^ can be used to extract not only the activation energy but also the Arrhenius-type pre-exponential factor τ_c,0_^–1^, defining τ_c_^–1^ via
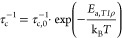
1where *k*_B_ denotes Boltzmann’s constant, and *T* is the temperature (Supporting Information, Section A3). The corresponding fits are shown as solid line parabolas, as shown in Figure 8. Derived activation energies are 0.18, 0.13, and 0.15 ± 0.01 eV for samples with *x* = 0.50, 0.67, and 0.75, respectively (Supporting Information Table SA5). The similarity between the activation energies measured for the two different timescales (*T*_1ρ_ versus *T*_1_ measurements) suggests jumps between connected sites, contributing to long-range diffusion instead of just a localized motion such as hopping between a finite number of nearby sites. To estimate Li-ion mobility at finite temperatures, we calculate values for the Li-ion jump rate, τ_c_^–1^, at 25 °C, leading to 2.8, 4.1, and 3.1 ± 0.5 × 10^5^ s^–1^ for samples with *x* = 0.50, 0.67, and 0.75, respectively. The highest τ_c_^–1^ and the lowest activation energy are found for the *x* = 0.67 composition. In the model used here, a slightly lower decrease of *R*_1ρ_ at the low temperature flank was accounted for by using a stretched exponential correlation function, indicating correlated motion. This may be attributed to a vacancy diffusion mechanism and/or structural disorder introduced by ball milling, which can cause (apparent) correlation effects such that the data can no longer be fit using a single exponential NMR correlation function.^71,77,78^

### Electrochemical Impedance Spectroscopy

**Figure 9:**
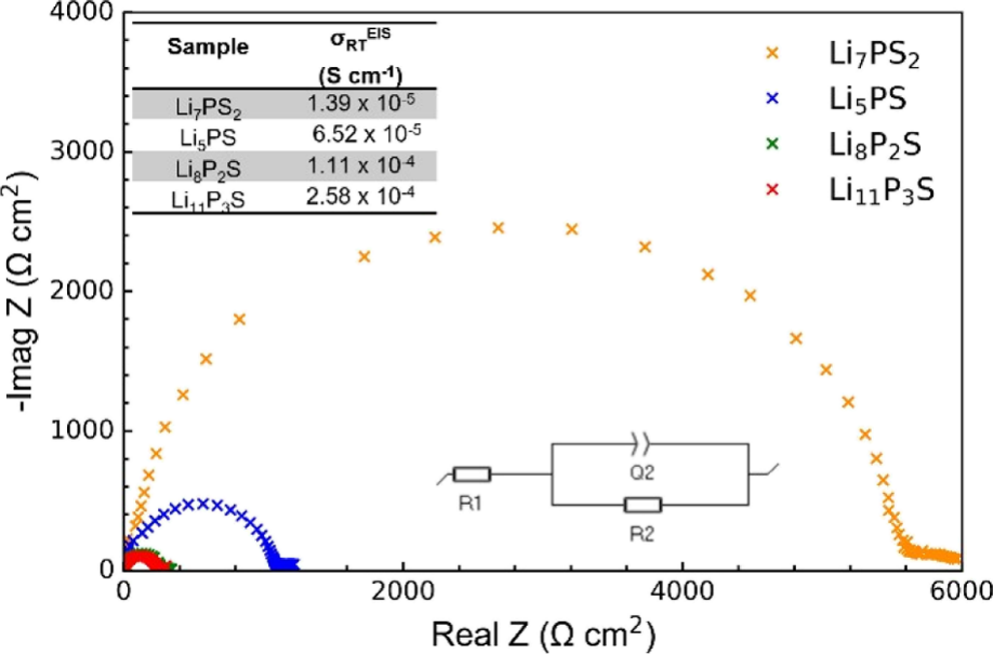
Room temperature EIS plots of Li|SE|Li cells for different compositions of *x*Li_3_P-(1 – *x*)Li_2_S. The used equivalent circuit model is indicated. The inset at the top shows the obtained room temperature ionic conductivity of each composition. The obtained *R*1, *R*2, and *Q*2 values after fitting the data with this equivalent circuit can be found in Supporting Information Table SA3.

Since NMR relaxometry inherently measures more local ion movements, we also used VT EIS to probe the Li ion mobility further. The bulk conductivity of each of the SE was studied using Li|SE|Li symmetric cells. Room temperature impedance plots of all four samples are shown in Figure 9. Since a single semicircle is observed in EIS plots for all samples, grain (bulk) and grain boundary contributions (if any) cannot be differentiated. Note that small deviations from a perfect semi-circle may indicate unresolved contributions from grains and grain-boundaries. In some cases, an additional depressed small semicircle at lower frequency is observed, which we assign to the interface between Li metal and the SE surface. A simple equivalent circuit (Figure 9, bottom inset) was used to fit the main semi-circle and calculate the impedance parameters (*R*_1_, *R*_2_, *Q*_2_, and *a*_2_) and ionic conductivity, and the values of the parameters obtained from the fit are listed in Supporting Information Table SA3. A better fit was achieved using a constant phase element *Q* (with the measure of non-ideality, *a* ∼ 0.93), which describes an imperfect capacitor; this is tentatively attributed to the inherent disorder in the bulk or porosity, leading to electrode surface heterogeneity. The total conductivity (Figure 9, upper inset) was calculated using *R*_2_ values and taking the geometrical area of Li metal into account, resulting in Li-ion conductivities from around 10^–5^ to 10^–4^ S cm^–1^ at RT. A noticeable increase in Li ion conductivity with increasing Li_3_P content is observed. The extracted solid solution conductivities are significantly higher than those of the pure endmember phase Li_2_S, which is known to be a poor conductor (10^–14^ to 10^–10^ S cm^–1^, Supporting Information Table SB3),^61,79^ and Li_3_P, which was verified under identical experimental conditions (1.81 × 10^–7^ S cm^–1^ for Li electrodes and around 7 × 10^–8^ S cm^–1^ for Au blocking electrodes, Supporting Information Figure SA10). We note that reported conductivities of lithium binaries or ternaries can range over 1–4 orders of magnitude.^79^ The ionic conductivity of Li_3_P is still under debate, with reported values ranging from around 10^–4^ to 10^–8^ S cm^–1^ (see below),^62,80^ requiring further investigation on the origin of this discrepancy.

**Figure 10:**
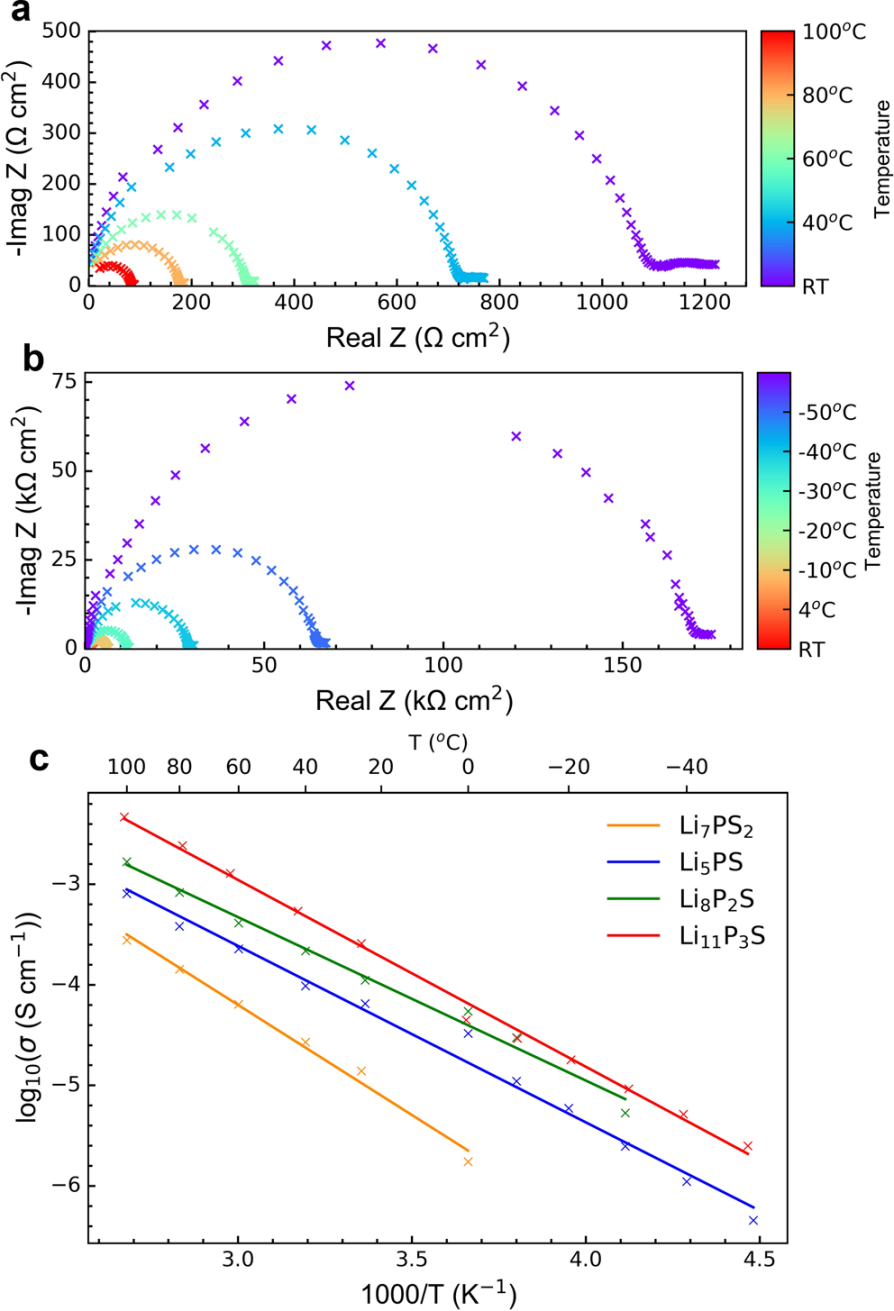
Representative EIS plots from Li|SE|Li cells for the *x* = 0.5 sample at (a) 100 °C to RT and (b) RT to −50 °C. (c) EIS Arrhenius plots showing the temperature dependence of the total ionic conductivity for different *x*Li_3_P-(1 – *x*)Li_2_S compositions.

Representative VT EIS plots for temperatures from 100 °C to RT and RT to −50 °C for the sample with *x* = 0.5 are shown in Figure 10a,b, respectively. A linear dependence of conductivity with temperature is observed for all samples. The VT EIS data were fit using the same approach as used for the RT EIS data. Representative plots are shown in Supporting Information Figure SA9 with impedance parameters for all the samples at different temperatures tabulated in Supporting Information Table SA3. The activation energies *E*_a_ derived from the Arrhenius plots are in the range of 0.2–0.15 eV (Figure 10c and Table 2). These values agree with the values obtained from the NMR relaxometry measurements where the sample with *x* = 0.67 shows the lowest *E*_a_. The increased ionic conductivity of the *x* = 0.75 sample, despite having a similar activation energy as the *x* = 0.67 sample, is ascribed to the increase in charge carriers (i.e., Li interstitial).

### First-Principles Simulations of Li_2_S–Li_3_P Mixtures and Comparison with the Experiment

Given that the original structure search had not included solid solutions of the two end members of the tie line, new structure searches were required so as to compare the experiment and theory. The *x* = 0.67 composition was initially chosen (yielding the “Li_8_P_2_S” stoichiometry), given the computational feasibility (i.e.*,* size) of the structural supercell generated with this composition. For Model 1 (occupied octahedral voids, Li_2+2/3_(P_2/3_S_1/3_)_1_), a 3 × 3 × 3 cell is needed to preserve the molar P/S = 2:1 ratio (Figure 4b1), whereas for Model 2 (empty octahedral voids, Li_2_(P_0.5_S_0.25_□_0.25_)_1_), a 2 × 2 × 2 cell can account for the Li/S = 8:1 ratio (Figure 4b2). The lowest-energy relaxed geometries corresponding to the models and their formation energies are shown in Supporting Information Figure SB7. Significantly higher formation energies for the phases resulting from Model 2 (149.1 versus 39.1 meV/atom for minimum-energy configurations/structures) are seen. Thus, vacancies in the anion lattice will likely yield phases that are significantly more energetically unfavorable, residing well above the hull, disfavoring structure Model 2. The presence of additional Li ions in the octahedral voids in Model 1 leads to only very minor changes in the intensities of the reflections in the XRD pattern, compared to the parent structure Li_2_S (Supporting Information Figure SB8).

From the electronic density of states (Supporting Information Figure SB9), structures following Model 1 are predicted to exhibit a wide band gap (*E*_g_ ≥ 1.6 eV), while structures following Model 2 are predicted to be metallic. Metallic conductivity is not supported by the yellow to red color of the synthesized samples.

**Figure 11:**
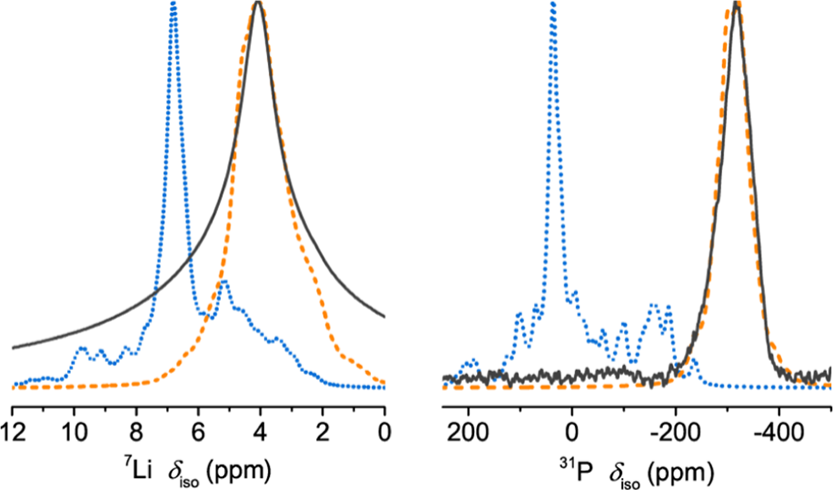
Predicted ^7^Li and ^31^P NMR spectra of the Li_8_P_2_S ternary, generated with the lowest energy DFT structures resulting from the two solid-solution models: Model 1, Li_2+2/3_(P_2/3_S_1/3_)_1_ (orange, dashed) and Model 2, Li_2_(P_0.5_S_0.25_□_0.25_)_1_ (blue, dotted), compared to the experimental spectra (black).

Next, the ^7^Li and ^31^P NMR shifts computed by DFT/GIPAW for the *x* = 0.67 structure were compared to the experimental spectra (Figure 11). For generating these NMR spectra, the individual contributions from the low-lying defect configurations from either structure model were summed together using their Boltzmann weights at 298 K as scaling factors. The sum of Model 1 configurations reproduces the experimentally observed features well for both ^7^Li and ^31^P NMR spectra, whereas the Model 2 structures lead to spectral features and shifted peak positions that bear very little resemblance to the experimental spectra, again favoring Model 1 over Model 2.

Thus, based on the combined findings of X-ray and neutron diffraction, NMR experimental data, and quantum-chemical calculations, we propose an anti-fluorite-based structure model with a fully occupied mixed S^2–^/P^3–^ anion lattice with lithium ions (partially) occupying the tetrahedral and octahedral sites, and the vacancies allowing Li ion hopping. This assignment is supported by known isostructural phosphide ternaries exhibiting a ccp P^3–^ lattice with cations occupying 8*c* sites completely and 4*b* sites partially.^17−23,66^

**Figure 12:**
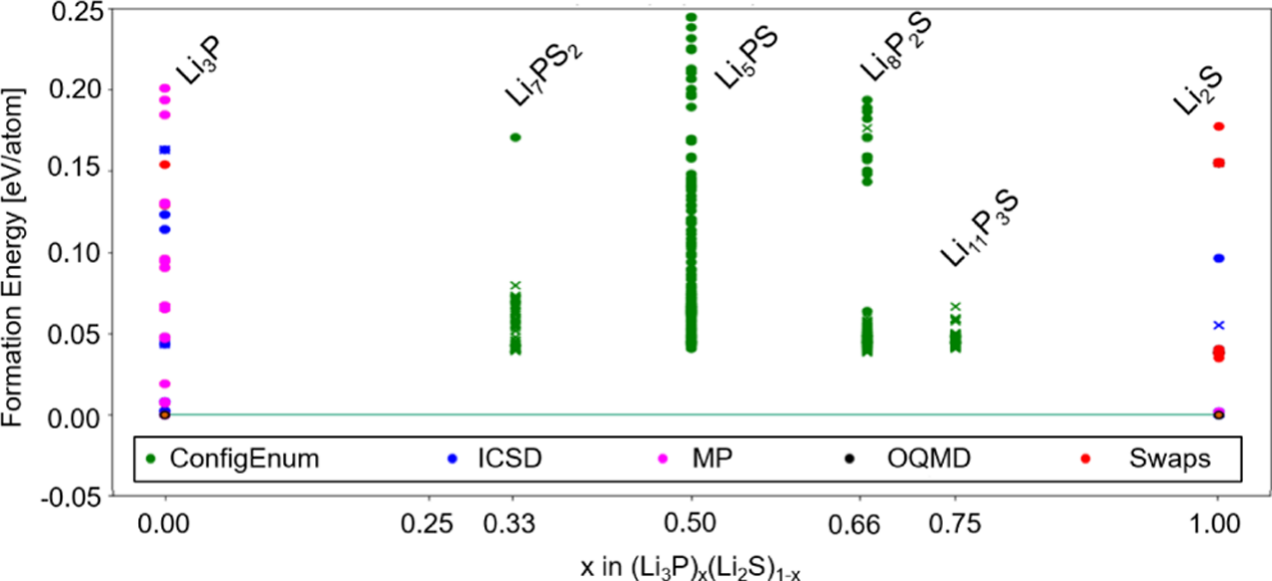
Predicted Li_3_P–Li_2_S pseudo-binary tie-line (at 0 K) focusing only on the end members and four ternaries considered in this study. For Li_2_S and Li_3_P, structures from different sources viz. ICSD,^81^ materials project,^82^ open quantum materials (OQMD)^83^ databases, elemental swaps with MATADOR,^52^ and configuration enumeration (CE) (ConfigEnum) are included and colored accordingly. For the ternaries, only the configuration-enumerated (CE) structures are available. Structures with the P1 symmetry are marked as cross, and others as filled circle.

Similar CE analyses for all compositions were then performed to yield a Li_2_S–Li_3_P pseudo-binary phase diagram, containing the low-lying configurations within 250 meV/atom from the hull (Figure 12). The minimum-energy configurations (i.e.*,* lowest points in Figure 12) generated for each ternary are visualized in Supporting Information Figure SB10. The structural features and the corresponding energetics of these configurations are summarized in Table 3. Inspection of the relaxed DFT structures revealed that some lithium atoms relaxed to off-center positions within the octahedral voids. A direct comparison between the DFT structure and that obtained from NPD was made for *x* = 0.5. (Note that the DFT calculation was performed within a 2 × 2 × 2 conventional unit cell, but the variety of atomic positions can be visualized by “folding” the atoms from all eight unit cells back into a single unit cell, as shown in Figure 5c). Both experiment and theory show considerable disorder of Li^+^ on and close to the interstitial site.

**Table 3:** Structural Features and Energetics of the Starting Binaries and the Resulting Lowest-Lying Ternary Mixtures

*x* in (Li_3_P)_*x*_(Li_2_S)_1-*x*_	chemical formula	hull distance [meV/atom]	unit cell volume [A^3^]
0.00	Li_2_S	0.0	185.6
0.33	Li_7_PS_2_	41.0	200.5
0.50	Li_5_PS	39.8	205.6
0.67	Li_8_P_2_S	39.1	209.6
0.75	Li_11_P_3_S	31.4	211.1
1.00	Li_3_P	0.0	117.2

The electronic conductivity in the new ternaries were again analyzed by computing the electronic band structures and DOS. Each ternary shows a wide, direct band gap (*E*_g_ > 1.4 eV), a property desired for a battery electrolyte (Supporting Information Figure SB11). Considering the known tendency of DFT to underestimate the band gaps, we expect the synthesized compounds to have even larger band gaps.

All ternary compounds are metastable at 0 K, against the parent materials, Li_2_S and Li_3_P (Table 2). However, they lie close to the hull (i.e., in the 31–41 meV/atom range), potentially rendering them thermally accessible at finite temperatures (e.g., 298 K). Furthermore, the presence of defect disorder involving the symmetrically equivalent crystallographic sites in the cubic anti-fluorite structure introduces configurational entropy that further stabilizes the ternary compounds. From the computed configurational entropy values (Supporting Information Table SB2), it can be predicted that the defect disorder stabilizes the ternaries by 1.2–10.5 meV/atom at 298 K. Finally, as shown for the samples with *x* = 0.67 (Supporting Information Figure SB10), the ternary structures correspond to local minima (i.e.*,* shallow potential wells or thermodynamic traps) on the corresponding potential energy surfaces, and they will thus be metastable over a finite temperature range.

The DFT-optimized lattice parameters show an increase in the unit cell volume with increasing amount of Li_3_P in the solid solution (Table 3), resembling the experimental XRD observations (Figure 3). The XRD patterns computed from the DFT-predicted minimum-energy configurations (Supporting Information Figure SB12) provide a good match with the corresponding experimental data.

**Figure 13:**
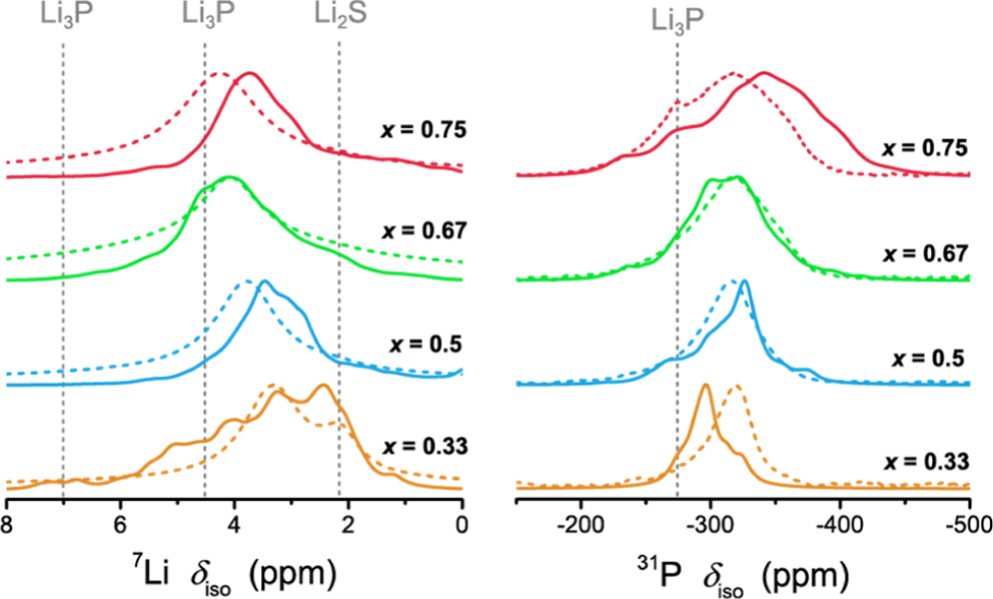
Comparison of the predicted ^7^Li and ^31^P NMR spectra (solid) and experimental spectra (dashed) for the new Li–P–S ternaries. Multiple defect configurations were summed and weighted based on their Boltzmann weights at 298 K to produce the final spectra. Note the Li_2_S impurity for the experimental spectra of the sample with *x* = 0.33 (Li_7_PS_2_).

To validate the predicted Li–P–S ternary structures further, we compare the computed ^7^Li and ^31^P NMR spectra to the experimental data (Figure 13). The calculated ^7^Li spectra represent a superposition of all the local configurations and do not account for any Li^+^ ion mobility. A good overall agreement with experiments can be noted in terms of the spectral shape, peak positions, and relative heights for both ^7^Li and ^31^P spectra of all ternaries, but the ^7^Li-calculated spectra contain broader resonances than the experimental spectra and multiple peaks. Again, this is consistent with rapid Li^+^ ion motion in the experimental samples, the calculations correctly predicting the changing ^7^Li shift with the composition. In addition, the position of the shoulder at lower shifts in the calculated spectrum of the sample with *x* = 0.5 resembles the second signal in the measured low temperature ^6^Li spectrum that we attributed to the occupied octahedral voids (Supporting Information Figure SA8). The calculated ^31^P spectra resemble the experimental ones and, of note, predict the increased linewidths for increasing phosphorus content, indicating the wider distribution of chemical environments of the phosphide ions. The predicted change of the center of gravity of the shift distribution with increasing phosphorus content is not, however, found in the experimental spectra, possibly because the calculations do not account for the mobility of the Li sublattice, which may affect the average local environment “seen” by the ^31^P nuclei.

### Li-Ion Transport Simulations

**Figure 14:**
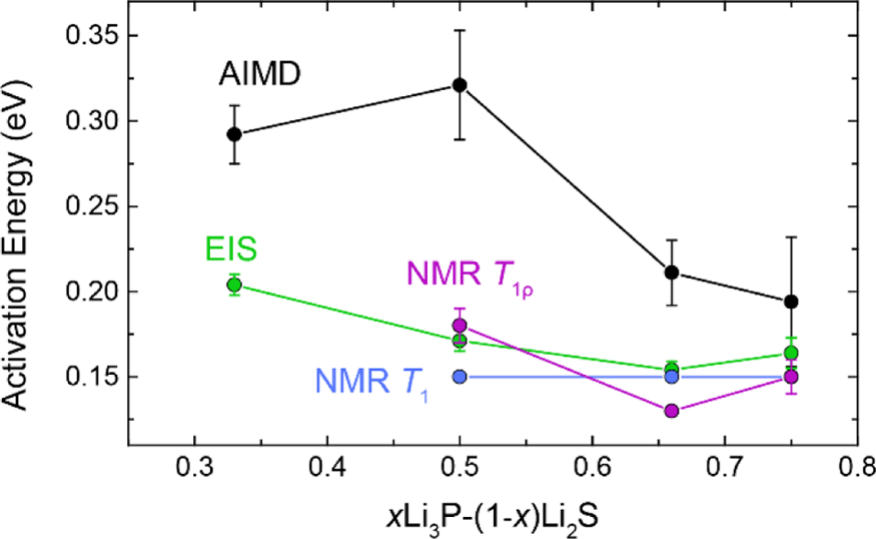
Predicted and experimental activation energies (*E*_a_) for Li_2_S–Li_3_P solid solutions. AIMD error bars show the standard deviation computed from the set of AIMD simulations of various input structures corresponding to a specific stoichiometry (Supporting Information Figure SB13). Experimental error bars show the standard deviation of the Arrhenius-type fit.

Finally, we compared the ionic conductivity () of the new ternaries, and the corresponding activation energies (*E*_a_) were computed using AIMD simulations and compared the results to well-known ternaries in the Li–P–S system with high conductivity, for example*,* Li_7_P_3_S_11_ and Li_3_PS_4_. The results are compiled in Supporting Information Table SB3 and Supporting Information Figure SB14 and reveal a reasonable agreement between our  and *E*_a_ values computed for the reference materials (Li_7_P_3_S_11_, Li_3_PS_4_, Li_2_S and Li_3_P) and the previous computational and experimental reports. While the values converge at higher phosphorus contents, the AIMD-derived activation energies are higher than those found by NMR and EIS (Supporting Information Table SB3; Figure 14). The finite sizes of the supercells used in the DFT simulations may also play a role, since they do not capture the random nature of the solid solutions synthesized experimentally, possibly explaining why the local transport measurements from NMR are even lower.

The presented solid solutions all show enhanced Li ion conduction compared to the endmembers Li_2_S (Supporting Information Table SB3) and Li_3_P (Supporting Information Figure SA10). The constituents with highest Li_3_P contents, that is, “Li_8_P_2_S” (*x* = 0.67) and “Li_11_P_3_S” (*x* = 0.75), display the highest total conductivities and lowest activation energies, comparable to those of the known superionic conductors Li_7_P_3_S_11_ (*E*_a_ = 160 meV) and β-Li_3_PS_4_ (*E*_a_ = 337 meV) (see Supporting Information Table SB3 for direct comparisons). The experimentally determined Li ion conductivity of Li_3_P (1.81 × 10^–7^ S cm^–1^) is similar to the recently reported conductivity of 3.0 × 10^–8^ S cm^–1^ at 50 °C. However, this study also reported a much higher activation energy of 0.53 eV.^62^ By contrast, in an early study of Li_3_P conductivities of 6.6 × 10^–4^ S cm^–1^ at close to room temperature were reported, now with a much lower activation energy of 0.18 eV that is close to the value measured in our work.^80^ The large discrepancies between the two studies suggest that defects and/or non-stoichiometry may enhance the transport properties within the Li_3_P structure type. Consistent with this, AIMD simulations of the stoichiometric phase give an even higher activation barrier of approximately 700 meV with such low Li^+^ conductivities that higher values could only be estimated above 500 K. These studies motivate further studies of samples with the composition beyond *x* = 0.75 and on the role of non-stoichiometry on the transport properties of Li_3_P.

The enhanced conductivity in the new Li–P–S ternaries (compared to the parent Li_2_S material) even at low temperatures can be ascribed to a knock-on mechanism, facilitated by the interstitial Li defects, as demonstrated for similar solid ion-conducting systems.^84−86^ As determined for other lithium phosphide ternaries with ccp phosphide lattices,^17,18,22,23^ lithium hopping is expected to occur via face-sharing octahedral and tetrahedral sites. For higher *x*, increased lattice parameters lower the hopping activation barrier, and phosphide anions stabilize higher energy octahedral sites through Coulomb interactions.

## Conclusions

The synthesis, structural elucidation, and ionic conductivity measurements, supported by quantum-chemical calculations, of a new *x*Li_3_P–(1 – *x*)Li_2_S solid solution, have been reported in this work. Upon high-energy ball-milling of the two binary compounds, a solid solution of the Li_2_S anti-fluorite structure type is formed within the compositional range of 0.39 ≤ *x* ≤ 0.75, which features a disordered anion lattice with tetrahedral and octahedral voids (partially) filled by mobile Li ions. These materials combine high conductivity with inherent redox stability toward Li metal and can thus be regarded as potential SEs for Li metal batteries.

The work illustrates that Li_3_P solubility in Li_2_S can occur to form phases that are close to the thermodynamic ground state; these phases may be accessible under electrochemical conditions where metastable phases are often formed if they are more kinetically accessible. Furthermore, since Li_2_S and Li_3_P are known to form as degradation products at the interphase between Li metal and P- and S-containing SEs, these solid solutions may form as part of the SEI/degradation process, with implications for the conductivity of the SEI.^87^ The formation of these phases has particular importance when evaluating potential degradation products of different Li–P–S ternaries and quaternaries involving other metal ions (e.g., in Ge and Si) in the various solid-state conductors used for LIBs. Indeed, ^31^P NMR spectra exhibiting similar chemical shifts and line shape were recently measured for interphase products between the SE Li_7_SiPS_8_ and Li metal, indicating that the metastable solid solutions reported here might intrinsically arise at thiophosphate–Li interfaces.^88^ Finally, we note that Li_2_S–Li_3_P intergrowths, as identified via high-throughput CSPs, suggest potential intergrowths or grain boundaries between the Li_2_S and Li_3_P phases that should be considered when analyzing the highly heterogeneous SEIs that form in these systems.

Future work on the solid solutions will investigate the electrochemical reactions at the interface of Li metal, decomposition at high potentials, and the overall performances of these solid solutions in SSBs. While further studies are needed to explore this, the conductivity bottleneck and contact issues between sulfide SEs and lithium metal anodes may potentially be reduced by using the Li_3_P–Li_2_S solid solutions, either as part of a composite structure or when formed electrochemically. We anticipate that this class of materials with a fully reduced anion lattice opens a new pathway toward stable, high-capacity solid-state batteries based on lithium metal anodes.

## Supporting Information Available

Additional experimental and computational methodology details, solid solution photographs, neutron diffraction data, electrochemical impedance data, NMR relaxometry calculations, and results from first-principles calculations (PDF)
