# Supplementary material for: Forced Disorder in the Solid Solution Li3P–Li2S: A New Class of Fully Reduced Solid Electrolytes for Lithium Metal Anodes
Source: J Am Chem Soc. 2022 Aug 30;144(36):16350–65. doi: 10.1021/jacs.2c01913 (PMC9479069; doi:10.1021/jacs.2c01913)
Supplement: Supplementary file 1 — ja2c01913_si_001.pdf [file ja2c01913_si_001.pdf]

# Supporting Information

## Forced Disorder in the Solid Solution $Li_3P$–$Li_2S$: A New Class of Fully Reduced Solid Electrolytes for Lithium Metal Anodes

Conrad Szczuka,[‡,1,2,3] Bora Karasulu,[‡1,4] Matthias F. Groh,[‡,1] Farheen N. Sayed,[1,5] Timothy J. Sherman,[1] Joshua D. Bocarsly,[1,5] Sundeep Vema,[1,5] Svetlana Menkin,[1,5] Steffen P. Emge,[1] Andrew J. Morris[6] and Clare P. Grey*[,1]

[1] Yusuf Hamied Department of Chemistry, University of Cambridge, Lensfield Road, Cambridge CB2 1EW, United Kingdom

[2] Institute of Energy and Climate Research (IEK-9), Forschungszentrum Jülich GmbH, 52425 Jülich, Germany

[3] Institute of Physical Chemistry, RWTH Aachen University, 52056 Aachen, Germany

[4] Department of Chemistry, University of Warwick, Gibbet Hill Road, Coventry, CV4 7AL, United Kingdom

[5] The Faraday Institution, Quad One, Harwell Campus, Didcot, OX11 0RA, United Kingdom

[6] School of Metallurgy and Materials, University of Birmingham, Birmingham, B15 2TT, United Kingdom

**Table of contents**

Part A. Additional Experimental Methodology Details and Results ..... 2
1. Structural Characterisation ..... 2
2. Electrochemical Impedance Spectroscopy ..... 10
3. NMR Relaxometry Analysis ..... 12

Part B. Additional Computational Methodology Details and Results ..... 15
1. Computational Methods ..... 15
2. Preparation of the simulation models for the $Li_2S$:$Li_3P$ solid mixtures ..... 19
3. Discovering New Li-P-S Phases with Stochastic Structure Predictions (AIRSS) ..... 20
4. Supporting Tables and Figures ..... 21
5. Li-ion transport simulations using AIMD ..... 36

References ..... 39

# Part A. Additional Experimental Methodology Details and Results

## 1. Structural Characterisation

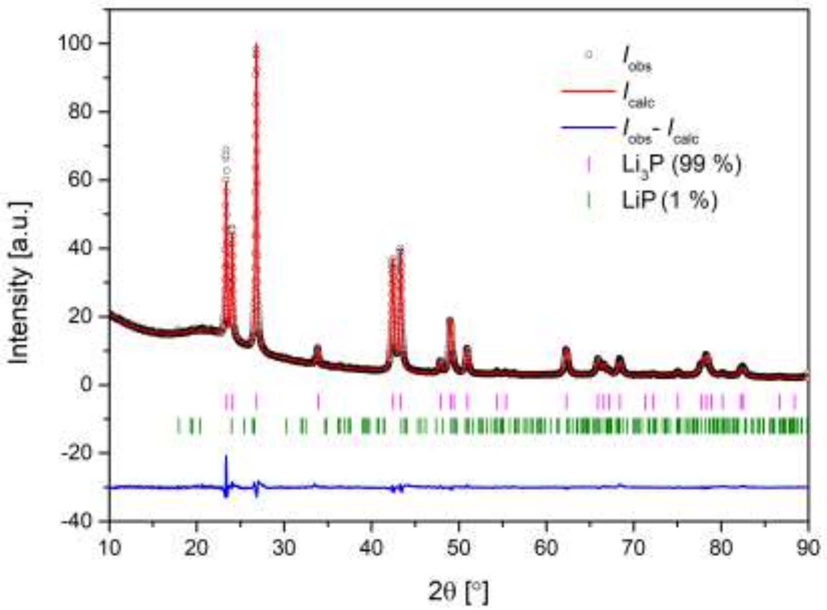


**Supporting Figure SA1.** Rietveld refinement of laboratory powder pXRD pattern of synthesised $Li_3P$.

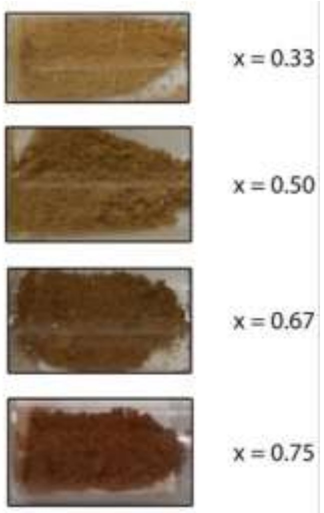


**Supporting Figure SA2.** Photographs of ball-milled samples.

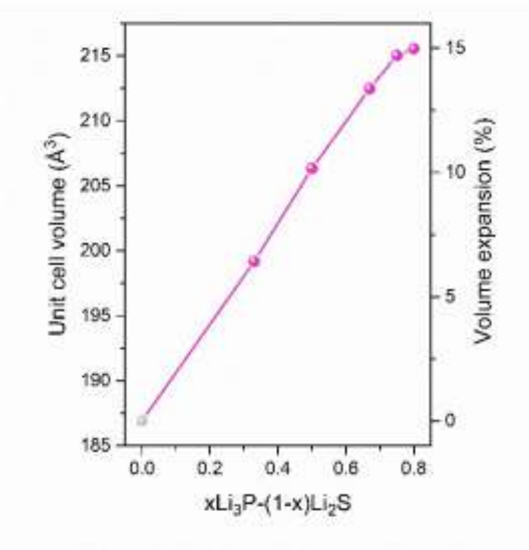


**Supporting Figure SA3.** Increase of unit cell volume for the solid solutions with increasing $Li_3P$ content showing Vegard's law.  Note that the x = 0.8 sample is outside the limits of the solid solution.

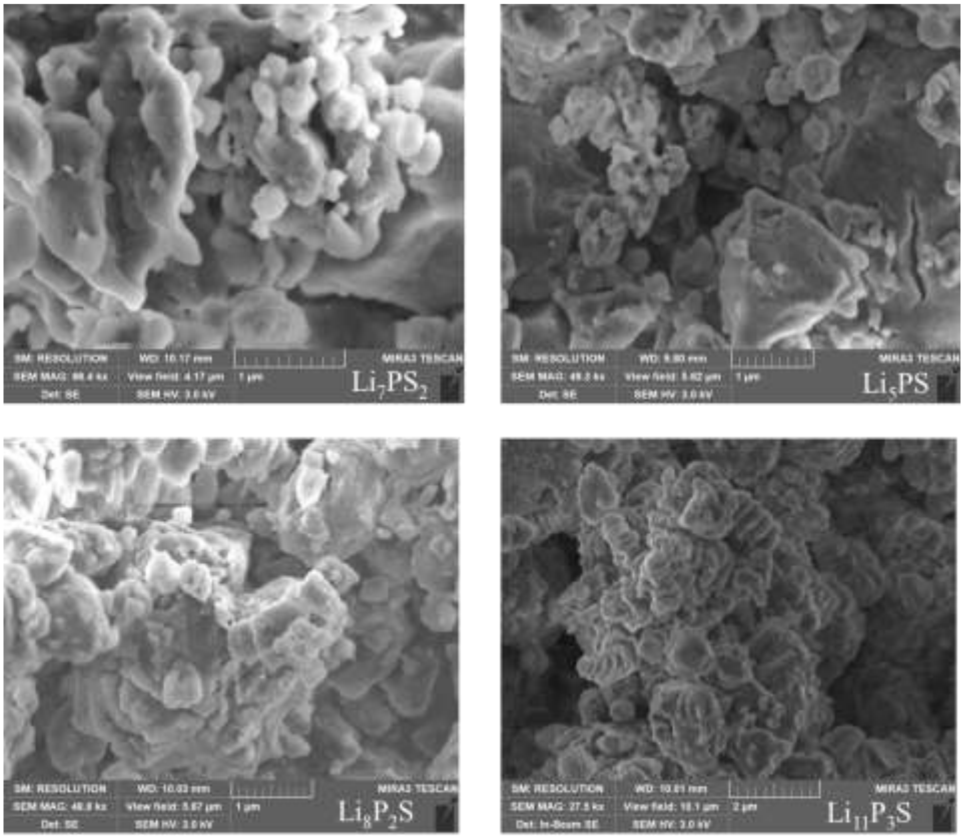


**Supporting Figure SA4**. SEM images of all four powder samples. For microscopy, powder samples were carefully sprinkled over carbon conductive tape placed on an aluminum sample stub. The samples were transferred into the SEM chamber in a transfer module (Kammrath & Weiss, type CT0)) under argon. SEM

images were taken with a Tescan MIRA3 FEG-SEM instrument at an acceleration voltage of 3.0 kV using the secondary electron mode.

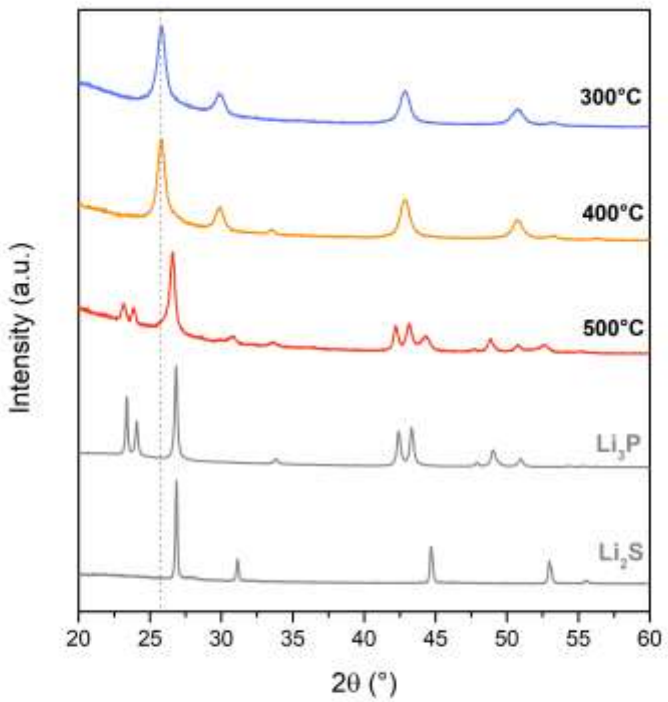


**Supporting Figure SA5.** Room-temperature laboratory powder XRD patterns of ball milled and subsequently annealed samples (annealing temperatures indicated) with $x = 0.67$. For comparison, $Li_3P$ and $Li_2S$ patterns are appended, depicted in grey. Note the decomposition of the solid solution into $Li_2S$ and $Li_3P$ at elevated temperatures.

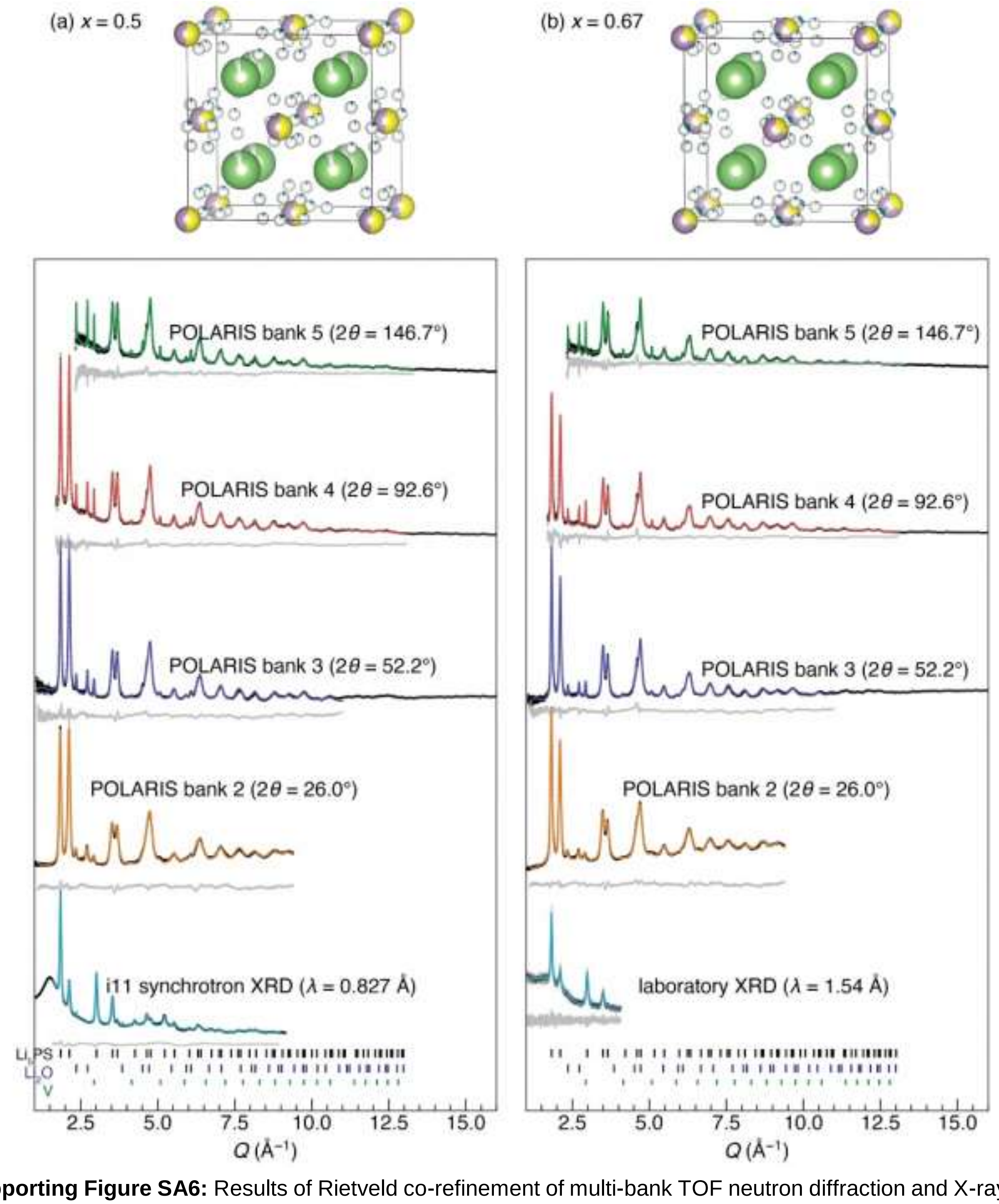


**Supporting Figure SA6:** Results of Rietveld co-refinement of multi-bank TOF neutron diffraction and X-ray diffraction for two samples. For the $x$ = 0.5 sample (a), synchrotron diffraction is used for the X-ray component, but for the $x$ = 0.67 sample, synchrotron diffraction was not available and a laboratory XRD pattern is used instead. Both patterns are refined within “model 1” (as defined in the main text), consisting

of the antifluorite structure with additional lithium at disordered positions within the octahedral voids (see tables SA1 and SA2).

**Supporting Table SA1:** Results of joint refinement of synchrotron and time-of-flight neutron powder diffraction data for sample with $x$ = 0.5 (nominal composition $Li_5PS$). For refined parameters, the values in parentheses indicate the refinement uncertainty in the last reported digit(s).

| | |
|---|---|
| **temperature** | ambient |
| **space group** | *Fm*-3*m* (225) |
| ***a* (Å), X-ray** | 5.8991(3) Å |
| ***a* (Å), neutron** | 5.8919(4) Å |
| **refined composition** | $Li_{5.09(2)}P_{1.05(3)}S_{0.95(3)}$ |

| **Refined pattern** | | **refined range (2θ/° or tof/µs)** | $R_{wp}$ **(%)** | $R_{exp}$ **(%)** |
|---|---|---|---|---|
| X-ray (DLS i11, mythen) | $\lambda$ = 0.827 Å | 12° – 72° | 3.100 | 1.238 |
| neutron, POLARIS bank 2 | 2θ = 26.0° | 700 µs – 10000 µs | 1.166 | 0.447 |
| neutron, POLARIS bank 3 | 2θ = 52.2° | 1930 µs – 19000 µs | 1.411 | 0.490 |
| neutron, POLARIS bank 4 | 2θ = 92.6° | 2600 µs – 20000 µs | 0.898 | 0.342 |
| neutron, POLARIS bank 5 | 2θ = 146.7° | 3500 µs – 22000 µs | 0.865 | 0.578 |
| overall | | | 1.612 | 0.660 |

| **site** | **occupancy** | **Wyckoff label** | ***x*** | ***y*** | ***z*** | $B_{iso}$ |
|---|---|---|---|---|---|---|
| Li1 | 0.941(3) Li | 8c | 0.25 | 0.25 | 0.25 | 1.53(3) |
| S1 | 0.527(16) S / 0.473(16) P | 4a | 0 | 0 | 0 | 0.81(1) |
| Li2 | 0.126(5) Li | 4b | 0.5 | 0.5 | 0.5 | 1 |
| Li3 | Li | 48i | 0.6111(7) | 0.6111(7) | 0.5 | 1 |

| | |
|---|---|
| **impurity** | $Li_2O$ (2.54(5) wt-%) [*Fm*-3*m*, *a* = 4.6174(6) Å] |
| | Li (8c), $B_{iso}$ = 1.00(9) |
| | O (4a) $B_{iso}$ = 0.68(5) |

**Supporting Table SA2:** Results of joint refinement of synchrotron and time-of-flight neutron powder diffraction data for sample with *x* = 0.67 (nominal composition $Li_8P_2S$). For refined parameters, the values in parentheses indicate the refinement uncertainty in the last reported digit(s).

| | |
|---|---|
| **temperature** | ambient |
| **space group** | *Fm*-3*m* (225) |
| ***a* (Å), X-ray** | 5.953(4)  Å |
| ***a* (Å), neutron** | 5.9531(3) Å |
| **refined composition** | $Li_{8.69(3)}P_{2.08(8)}S_{0.93(8)}$ |

| **Refined pattern** | | **refined range (2θ/° or tof/μs)** | $R_{wp}$ **(%)** | $R_{exp}$ **(%)** |
|---|---|---|---|---|
| X-ray (laboratory) | *λ* = 1.54 Å | 5° – 60° | 7.051 | 6.345 |
| neutron, POLARIS bank 2 | 2θ = 26.0° | 700 μs – 10000 μs | 1.057 | 0.429 |
| neutron, POLARIS bank 3 | 2θ = 52.2° | 1930 μs – 19000 μs | 1.446 | 0.477 |
| neutron, POLARIS bank 4 | 2θ = 92.6° | 2600 μs – 20000 μs | 1.025 | 0.341 |
| neutron, POLARIS bank 5 | 2θ = 146.7° | 3500 μs – 22000 μs | 0.880 | 0.574 |
| overall | | | 1.139 | 0.517 |

| **site** | **occupancy** | **Wycoff label** | ***x*** | ***y*** | ***z*** | $B_{iso}$ |
|---|---|---|---|---|---|---|
| Li1 | 1.0 Li | 8c | 0.25 | 0.25 | 0.25 | 0.59(3) |
| S1 | 0.31(3) S / 0.69(3) P | 4a | 0 | 0 | 0 | 1.14(2) |
| Li2 | 0.289(8) Li | 4b | 0.5 | 0.5 | 0.5 | 1.0 |
| Li3 | 0.0505(6) Li | 48i | 0.6034(7) | 0.6034(7) | 0.5 | 1.0 |

| | |
|---|---|
| **impurity** | $Li_2O$ (2.8(5) wt-%)  [*Fm*-3*m*, *a* = 4.6142(5) Å] |
| | Li (8c), $B_{iso}$ = 1.61(15) |
| | O (4a) $B_{iso}$ = 0.79(7) |

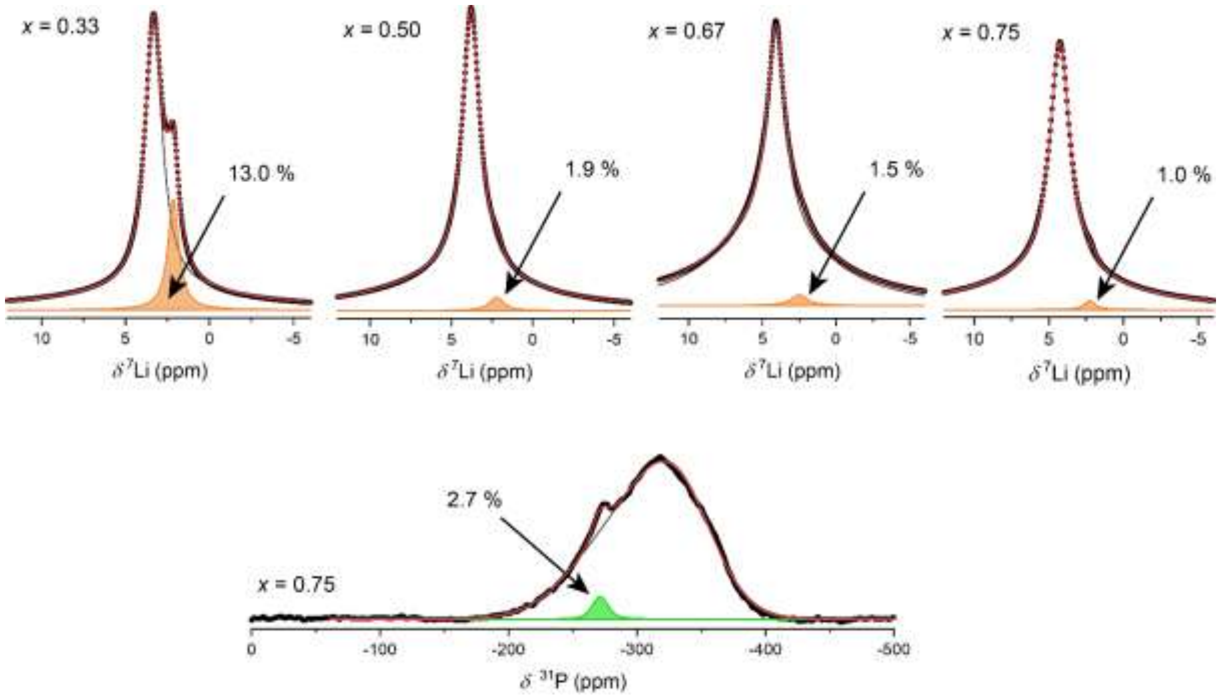


**Supporting Figure SA7.** Determination of residual $Li_2S$ and $Li_3P$ in synthesised solid solutions. The intensity contributions from $Li_2S$ (orange) to the $^7$Li NMR spectrum and from $Li_3P$ (green) to the $^{31}$P NMR spectrum are indicated with respect to the signal from the solid solutions, approximately fitted with two Lorentzian lines (grey lines). The experimental data (dots) and the overall fit (red line) are given.

Due to residual $Li_2S$ in the synthesised solid solutions, the *x* values that correspond to the composition of the solid solution need to be corrected. To calculate $x_{corr}$, we consider the following chemical equation

$$x\mathrm{Li_3P} - (1-x)\mathrm{Li_2S} \rightarrow a\,\mathrm{Li_2S} + (1-a)\,[x_{corr}\mathrm{Li_3P} - (1-x_{corr})\mathrm{Li_2S}] \qquad \text{(SA1)}$$

with the Li mass balance given by

$$3x + 2(1-x) = 2a + (1-a)(3x_{corr} + 2(1-x_{corr})) \qquad \text{(SA2)}$$

and the lithium site ratio as obtained from $^7$Li NMR given by

$$\frac{Integral(\mathrm{Li_2S})}{Integral(\text{Solid Solution})} = \frac{2a}{(1-a)(3x_{corr}+2(1-x_{corr}))} \qquad \text{(SA3)}$$

leading to corrections from $x$ = 0.33 to $x_{corr}$ = 0.39, $x$ = 0.50 to $x_{corr}$ = 0.51, and $x$ = 0.67 to $x_{corr}$ = 0.68. Taking into account $Li_2S$ but also $Li_3P$ residues from $^{31}$P NMR, the *x*-value of the sample with $x$ = 0.75 remains invariant.

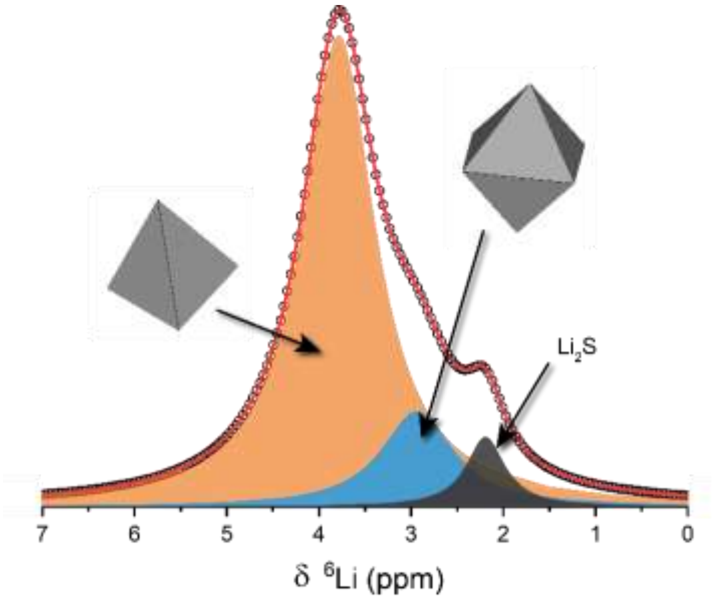


**Supporting Figure SA8.** $^6$Li MAS NMR spectrum (black circles) of $Li_{2+x}(P_xS_{1-x})$ for $x$ = 0.5 at −50 °C and 12.5 kHz spinning speed. The three-component Lorentzian fit (red line) comprises peak positions at 3.8 ppm and 3.0 ppm with an integral ratio of 5:1 (*i.e.* close to the ideal ratio of 4:1 for occupied tetrahedral and octahedral voids in the $Li_{2+0.5}(P_{0.5}S_{0.5})$ model) and trace amounts of $Li_2S$ at 2.2 ppm. Accordingly, the signal at 3.0 ppm can be tentatively assigned to Li ions in the octahedral void, since Li ions in octahedral sites generally resonate at lower frequencies than Li ions in tetrahedral sites, as observed for oxides[1,2], sulphides,[3–5] and phosphides.[6] Small deviations of the expected integral ratio can originate from a distribution of chemical shifts due to the disordered $S^{2-}$/$P^{3-}$ anion sublattice or residual Li motion.

## 2. Electrochemical Impedance Spectroscopy

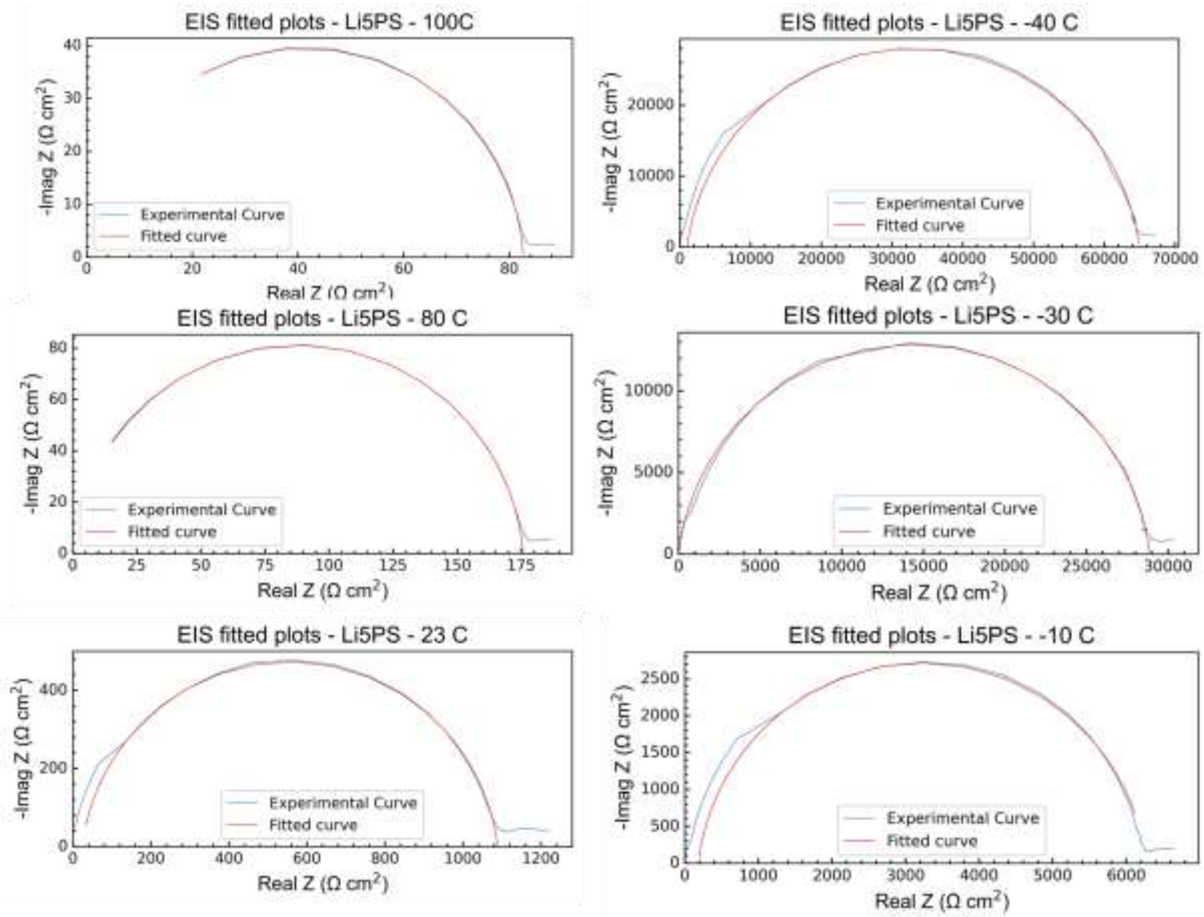


**Supporting Figure SA9.** Representative fitting of EIS plots at different temperature for *x* = 0.5 sample ($Li_5PS$). The data was noisy at higher frequencies due to the instrument limitations. The effect was more pronounced at lower temperatures (< 0 °C). Thus, the $R_1$ values have slightly higher uncertainty, whereas the $R_2$ values could be fitted with higher certainty due to the larger number of data points.

**Supporting Table SA3.** Experimental EIS parameters ($R_1$, $R_2$, $Q_2$, $a_2$) obtained for all $x Li_3P$-$(1-x)Li_2S$ samples. Conductivity (σ, S $cm^{-1}$) is calculated using $R_2$ (Ω, total resistance) of samples at each temperature. The values of $\chi^2/|Z|$ reflects the goodness of fit from EC Lab fitting program.

| ***x*=0.33** | **101 ºC** | **81ºC** | **60 ºC** | **40 ºC** | **RT** | **0 ºC** | **-10 ºC** | **-20 ºC** | **-30 ºC** | **-40 ºC** | **-50 ºC** |
|---|---|---|---|---|---|---|---|---|---|---|---|
| **$R_1$ (dev.)** | $1.55x10^{-12}$<br>(0) | 66.28<br>(1) | 34.46<br>(0.63) | 38.33<br>(0.74) | 75.21<br>(0.52) | 71.8<br>(0.30) | 15.66<br>(0.28) | | | | |
| **$R_2$ $x10^{+3}$ (dev.)** | 5.384<br>(1.04) | 10.501<br>(0.73) | 23.445<br>(3.00) | 55.596<br>(6.04) | 107.376<br>(3.93) | 861.643<br>(1.73) | 3814<br>(10.59) | | | | |
| **σ $x10^{-4}$** | 2.78 | 1.43 | 0.64 | 0.27 | 0.14 | 0.02 | 0.004 | | | | |
| **$Q_2$ $x10^{-10}$** | 1.90 | 1.32 | 1.81 | 1.88 | 1.63 | 1.58 | 2.21 | | | | |
| **$a_2$** | 0.98 | 1.00 | 0.98 | 0.97 | 0.98 | 0.98 | 0.96 | | | | |
| **$\chi^2$/\|Z\|** | $0.244x10^{-3}$ | 0.0354 | 0.055 | 0.102 | 0.219 | 0.385 | 0.491 | | | | |
| ***x*=0.5** | **100 ºC** | **80 ºC** | **60 ºC** | **40 ºC** | **RT** | **4 ºC** | **-10 ºC** | **-20 ºC** | **-30 ºC** | **-40 ºC** | **-50 ºC** |
| **$R_1$(dev.)** | $1.85x10^{-12}$<br>(0) | $1.92x10^{-12}$<br>(0) | 26.98<br>(2.27) | 31.75<br>(0.35) | 119<br>(1.2) | 37.93<br>(0.53) | 34.06<br>(0.41) | $11.88x10^{-12}$<br>(0) | 31.11<br>(0.32) | 18.76<br>(0.27) | 37.7<br>(0.25) |
| **$R_2$ $x10^{+3}$ (dev.)** | 1.752<br>(2.01) | 3.700<br>(0.50) | 6.194<br>(2.50) | 14.518<br>(1.70) | 21.680<br>(1.61) | 43.000<br>(2.41) | 128.400<br>(2.17) | 238.750<br>(0.79) | 570.095<br>(3.02) | 1277<br>(2.07) | 3104<br>(2.90) |
| **σ $x10^{-4}$** | 8.07 | 3.825 | 2.28 | 0.97 | 0.65 | 0.33 | 0.11 | 0.060 | 0.024 | 0.011 | 0.004 |
| **$Q_2$ $x10^{-10}$** | 2.40 | 3.31 | 2.77 | 3.07 | 2.52 | 3.27 | 3.70 | 3.33 | 2.31 | 2.255 | 1.92 |
| **$a_2$** | 0.97 | 0.95 | 0.96 | 0.95 | 0.96 | 0.96 | 0.94 | 0.94 | 0.96 | 0.96 | 0.97 |
| **$\chi^2$/\|Z\|** | $0.181x10^{-3}$ | $0.209x10^{-3}$ | 0.013 | 0.044 | 0.058 | 0.078 | 0.097 | 0.121 | 0.236 | 0.245 | 0.477 |
| ***x*=0.67** | **100 ºC** | **80 ºC** | **60 ºC** | **41 ºC** | **RT** | **0 ºC** | **-10 ºC** | **-20 ºC** | **-30 ºC** | **-40 ºC** | **-50 ºC** |
| **$R_1$(dev.)** | 38.01<br>(1) | $1.98x10^{-12}$<br>(0) | $1.05x10^{-12}$<br>(0) | 28.62<br>(1.68) | 29.84<br>(0.26) | 768<br>(4.04) | 46.9<br>(0.57) | | 55.6<br>(0.34) | | |
| **$R_2$ $x10^{+3}$ (dev.)** | 0.839<br>(0.41) | 1.707<br>(0.96) | 3.439<br>(0.86) | 6.484<br>(2.04) | 12.687<br>(1.50) | 25.832<br>(3.05) | 47.345<br>(3.203) | | 265.658<br>(2.07) | | |
| **σ $x10^{-4}$** | 16.87 | 8.29 | 4.11 | 2.18 | 1.11 | 0.54 | 0.30 | | 0.05 | | |
| **$Q_2$ $x10^{-10}$** | 2.04 | 3.14 | 3.90 | 3.14 | 3.23 | 5.30 | 2.75 | | 2.71 | | |
| **$a_2$** | 1.00 | 0.96 | 0.95 | 0.96 | 0.95 | 0.91 | 0.96 | | 0.96 | | |
| **$\chi^2$/\|Z\|** | $5.452x10^{-3}$ | $0.469x10^{-3}$ | $1.525x10^{-3}$ | 0.039 | 0.087 | $2.665x10^{-3}$ | 0.226 | | 0.452 | | |
| ***x*=0.75** | **100.6 ºC** | **79 ºC** | **62.7 ºC** | **42.1 ºC** | **RT** | **0.4 ºC** | **-10.2 ºC** | **-20.4 ºC** | **-30.6 ºC** | **-39.5 ºC** | **-49.2 ºC** |
| **$R_1$(dev.)** | 31.45<br>(0) | 22<br>(1) | 17.98<br>(1) | 12.12<br>(1) | $2.12x10^{-12}$<br>(0) | 11.55<br>(0.40) | 10.75<br>(0.37) | 10.33<br>(0.34) | 11.11<br>(0.31) | 10.17<br>(0.28) | 9.60<br>(0.25) |
| **$R_2$ $x10^{+3}$ (dev.)** | 0.232<br>(0.34) | 0.448<br>(0.54) | 0.844<br>(34) | 2.009<br>(0.76) | 4.209<br>(1.01) | 24.280<br>(2.33) | 37.027<br>(2.78) | 60.575<br>(2.79) | 117.843<br>(3.2) | 210.417<br>(2.85) | 433.708<br>(2.88) |
| **σ $x10^{-4}$** | 46.97 | 24.32 | 12.91 | 5.42 | 2.58 | 0.45 | 0.30 | 0.18 | 0.092 | 0.051 | 0.025 |
| **$Q_2$ $x10^{-10}$** | 1.92 | 1.59 | 1.56 | 1.39 | 0.323 | 1.49 | 1.50 | 1.50 | 1.43 | 1.45 | 1.46x |
| **$a_2$** | 1.00 | 1.00 | 1.00 | 1.00 | 0.94 | 1.00 | 1.00 | 1.00 | 1.00 | 1.00 | 1.000 |
| **$\chi^2$/\|Z\|** | $5.62x10^{-3}$ | 0.011 | 0.025 | 0.061 | $7.089x10^{-3}$ | 0.095 | 0.100 | 0.112 | 0.113 | 0.122 | 0.145 |

The impedance results for a solid electrode/electrolyte interface often reveal a frequency dispersion that cannot be described by simple elements such as a resistor, capacitor, or inductor or via convective diffusion (e.g., Warburg) impedance. The frequency dispersion is generally attributed to a "capacitance dispersion" expressed in terms of a constant-phase element (CPE).[7] The CPE behavior is generally attributed to surface/bulk heterogeneity and electrode porosity. In this work, the capacitance dispersion, represented by the CPE element, is attributed to the inherent heterogeneity/porosity in the samples of the Li-P-S electrolytes.

A simple model consisting of a resistor ($R_2$) in parallel to a CPE (Q) and in series to another resistor ($R_1$) was used for equivalent circuit fitting (the equivalent circuit is shown in Figure 9, insert). The capacitance of the CPE was used to determine the assignment of the components according to the assignment suggested by Irvine et al.[8] The Q-$R_2$ unit mainly represents the ion transport across the grain (bulk) and grain boundaries due to the $10^{-10}$ F capacitance typical for grain boundaries.[8] Interestingly, the SEI component (which is expected to result in CPE capacitance of $10^{-6}$-$10^{-7}$ F) does not contribute significantly to the impedance curve in this system.

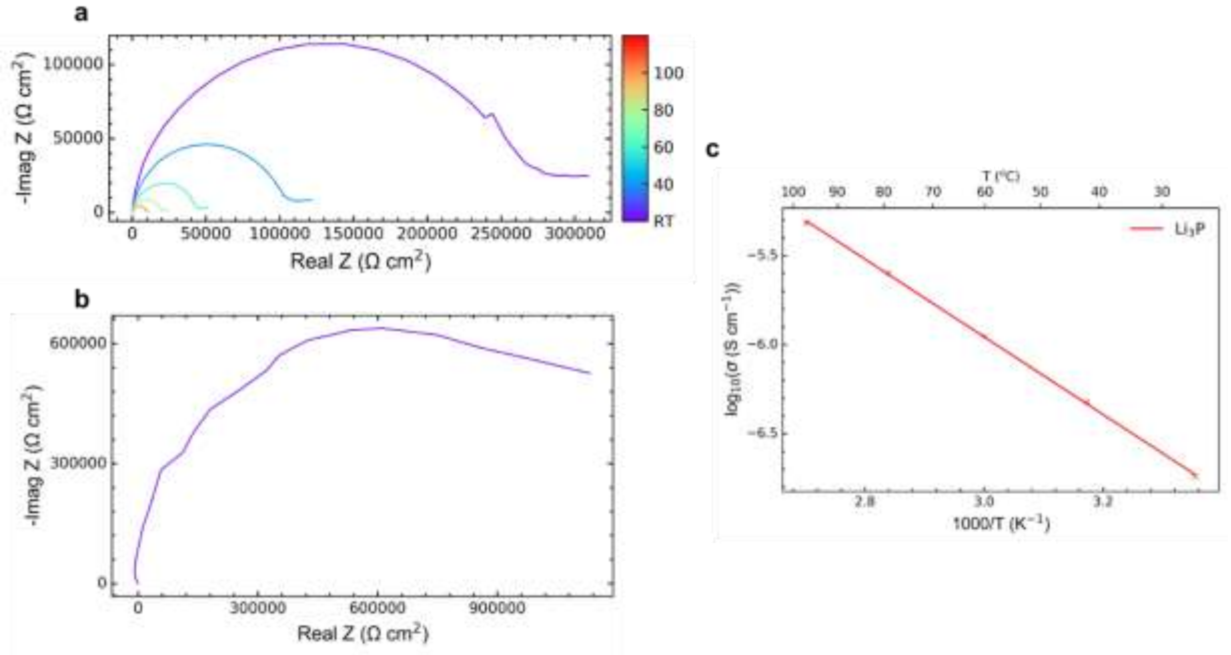


**Supporting Figure SA10.** (a) Representative EIS plots at different temperatures ranging from 100 °C to RT for synthesized $Li_3P$ ($x$ = 1). At RT, a conductivity of 1.81 x $10^{-7}$ S $cm^{-1}$ was extracted when using Li blocking electrodes. (b) Arrhenius plot of the extracted Li-ion conductivities from the data in (a) with a corresponding activation energy of 0.19 eV. We note that some samples appeared to react with both Li and Au blocking electrodes particularly after heating for prolonged periods at elevated temperature. This is illustrated in (c) which shows the RT EIS data of $Li_3P$ sandwiched between Au blocking electrodes. A Li-ion conductivity of 7.3 x $10^{-8}$ S $cm^{-1}$ was estimated from the higher frequency part of the curve. The reactivity

requires further investigation, but it may, at least in part, be the source of the wide variety of conductivities seen in the literature.

## 3. NMR Relaxometry Analysis

Relaxation processes in NMR are governed by fluctuating spin interactions, *e.g.* homo- and hetero-nuclear dipolar couplings and/or nuclear quadrupolar interactions for spins with spin quantum number I > 1/2. For the $x Li_3P$-(1−$x$)$Li_2S$ solid solution, Li-ions are the mobile species. Therefore, Li-ion mobility can be extracted from the relaxation times which were probed in the laboratory and rotating frame. For $T_{1\rho}$ relaxation, the temperature window containing the relaxation rate maximum allows analysis based on the Bloembergen-Purcell-Pound model.[9] The relaxation time in the rotating frame is given by[10–12]

$$\frac{1}{T_{1\rho}} = C[6\,J(2\omega_1) + 10\,J(\omega_0) + 4\,J(2\omega_0)] \qquad \text{(SA1)}$$

where $C$ is a proportionality constant reflecting the involved interaction strength(s) and $J$ is the spectral density of the motional process at the locking field frequency $\omega_1$ and Larmor frequency $\omega_0$. The spectral density function $J(\omega)$ originating from random three-dimensional jump processes can be expressed by

$$J(\omega) = \frac{\tau_c}{1+(\omega\tau_c)^{1+\alpha}} \qquad \text{(SA2)}$$

where $\alpha$ is the exponent of the underlying exponential correlation function and ranges from 0 to 1. Alternatively, $\beta = 1 + \alpha$ is used interchangably. $\alpha = 1$ reflects a Lorentzian-shaped $J(\omega)$ and is generally ascribed to uncorrelated three-dimensional motion, $\alpha < 1$ accounts for asymmetry of $J(\omega)$ and often indicates correlated motions when found on the low temperature side.[12,13] The correlation rate $\tau_c^{-1}$ is assumed to follow the Arrhenius relation

$$\tau_c^{-1} = \tau_{c,0}^{-1} \cdot \exp(-\,E_{a,T1\rho}/k_{\mathrm{B}}T) \qquad \text{(SA3)}$$

where $\tau_{c,0}^{-1}$ is the pre-exponential factor, $E_{a,T1\rho}$ is the activation energy (which can also be estimated from the high or low temperature flank, as shown for $T_1$ in the main text), $k_{\mathrm{B}}$ is Boltzmann's constant, and $T$ is the temperature.

By combining all three equations, an expression of the relaxation time $T_{1\rho}$ depending on the temperature $T$ can be extracted with adjustable parameters $C$, $\tau_{c,0}^{-1}$, $E_{a,T1\rho}$, and $\alpha$ or $\beta$. Corresponding fits to the experimental data with the following fit parameters are shown in Figure 10.

**Supporting Table SA4.** Parameters from modified BPP analysis by fitting the $T_{1\rho}$ data.

| $x$ | $\boldsymbol{E_{a,T1\rho}}$ (eV) | $\boldsymbol{\tau_{c,0}^{-1}}$ (s$^{-1}$) | $\boldsymbol{C}$ (s$^{-2}$) | $\boldsymbol{\beta}$ |
|---|---|---|---|---|
| **0.50** | $0.18 \pm 0.01$ | $3.0 \pm 0.7 \times 10^{8}$ | $2.80 \pm 0.04 \times 10^{8}$ | $1.74 \pm 0.07$ |
| **0.67** | $0.13 \pm 0.01$ | $6.5 \pm 0.7 \times 10^{7}$ | $2.80 \pm 0.01 \times 10^{8}$ | $1.95 \pm 0.05$ |
| **0.75** | $0.15 \pm 0.01$ | $1.2 \pm 0.2 \times 10^{8}$ | $3.40 \pm 0.04 \times 10^{8}$ | $1.95 \pm 0.09$ |

Using equation 3, the correlation time for any temperature can be calculated, which is assumed to be equal within a factor of unity to the residence time $\tau$, the inverse of the average jump rate $\tau^{-1}$. From these and the

jump distance *l* (given in Table SA5), the Einstein-Smoluchowski equation (equation SA4) can be used to calculate the diffusion coefficient and subsequently the conductivity.[14]

$$D = f\frac{l^2}{6\tau} \qquad \text{(SA4)}$$

For the correlation factor we assume $f = 1$ as approximation even if there is evidence of minor correlated motion due to $\beta$ <2. With these diffusion coefficients, with the Nernst-Einstein equation (equation AS5), the appropriate conductivity value can be calculated.

$$\sigma = D\frac{e^2 N}{H_R k_B T} \qquad \text{(SA5)}$$

with *N* as the charge carrier density, i.e. Li-ions per unit cell volume, the elemental charge *e* and the Haven ratio, for which the approximation $H_R = f = 1$ is used.

**Supporting Table SA5.** Parameters and constants used to calculate diffusion coefficients and conductivity values from NMR measurements. For *l* the shorter tetrahedral-octahedral distance was used. *N* was calculated from the unit cell volume (see Table 2) and the number of Li ions per unit cell = 4(2+x). *D* and $\sigma$ values were exemplarily calculated for $T$ = 300 K.

| x | $\boldsymbol{l}$ (Å) | $\boldsymbol{N}$ ($m^{-3}$) | $\boldsymbol{D}$ ($cm^2\ s^{-1}$) | $\boldsymbol{\sigma}$ ($S\ cm^{-1}$) |
|---|---|---|---|---|
| **0.50** | 2.557 | 4.85 x $10^{28}$ | 3.22 x $10^{-11}$ | 0.97 x $10^{-5}$ |
| **0.67** | 2.582 | 5.04 x $10^{28}$ | 4.73 x $10^{-11}$ | 1.5 x $10^{-5}$ |
| **0.75** | 2.592 | 5.13 x $10^{28}$ | 3.61 x $10^{-11}$ | 1.2 x $10^{-5}$ |

# Part B. Additional Computational Methodology Details and Results

## 1. Computational Methods

*Density Functional Theory Calculations*

Plane-wave density-functional theory (DFT) electronic structure calculations were performed using the CASTEP code[15,16] (v. 17.21), which is an implementation of periodic boundary conditions and the pseudopotential approximation. The generalised-gradient approximation (GGA) was used of the Perdew–Burke–Ernzerhof (PBE) exchange-correlation functional form.[17] The Brillouin zone was sampled using a Monkhorst–Pack (MP) grid[18] with a **k**-point spacing finer than $2\pi \times 0.05$ Å$^{-1}$. Plane-wave basis sets were truncated at a cut-off energy of 800 eV and a convergence criterion of $1 \times 10^{-8}$ eV/atom was used in the self-consistent optimisation of the electronic wavefunction. The atomic positions and lattice parameters were fully relaxed at this level of accuracy using LBFGS optimizer[19,20] until all forces were smaller than 0.05 eV/Å. All *ab initio* calculations employ the ultra-soft pseudopotentials generated by the CASTEP on-the-fly generator (v.17), using the following strings: Li:1|1.0|14|16|18|10U:20(qc=7); P: 3|1.8|4|4|5|30:31:32; S: 3|1.8|5|6|7|30:31:32.

All the structures reported here were obtained using the high-accuracy DFT settings as described above. However, during the preliminary configuration enumeration and structure prediction procedures (described below), which were computationally intensive, as required for the relaxation of thousands of structures, we had to start from lower-accuracy settings so as to limit the computational efforts. For those calculations, we used a lower cut-off energy of 300 eV along with Vanderbilt 'ultrasoft' pseudopotentials (with softer cut-off requirements), while other DFT settings were not altered. For a selection of low-energy structures, we then performed the 'refinement' calculations with the final level of accuracy.

*Generating Phase Diagrams*

Maxwell constructions can be used to compare the relative stabilities of the Li-P-S ternaries.[21] As per the second law of thermodynamics, the (free) energy per atom is a convex function of the relative concentrations of the atoms. The relative stability of each ternary with respect to the constituent elements (solid Li, P and S) can thus be expressed in terms of its formation energy per atom (*$E_f$/atom*) against its fractional/molar content of the atomic species, often referred to as a phase diagram. Here, we define the formation energy per atom of a compound $Li_xP_yS_z$ as

$$Ef/atom = \frac{E\{LixPySz\} - x\mu\{Li\} - y\mu\{P\} - z\mu\{S\}}{x+y+z} \quad \text{(SB1)}$$

where x, y and z are the molar amounts of Li, P and S in the compound, respectively. In addition, $E(Li_xP_yS_z)$ is the DFT energy of a given ternary structure, while $\mu_{Li}$, $\mu_P$ and $\mu_S$ are the chemical potentials of the atomic species in their ground-state structure (DFT energy per fu), i.e., Li is in the $Im\overline{3}m$ space group, P in the $Cmca$ and S in the $P2/c$. The fractional concentration of each atomic species in each $Li_xP_yS_z$ compound is defined as

$C_{Li} = \frac{x}{x+y+z}; C_P = \frac{y}{x+y+z}; C_S = \frac{z}{x+y+z}$ (SB2)

where $C_{Li} + C_P + C_S = 1$. Then, starting at ($C_{Li/P/S}$, $E_f/atom$) = (0,0) and ending at (1,0), we join together the lowest possible points with tie-lines, while maintaining a convex function (i.e., each tie-line has a gradient equal to or larger than the last). Hence, we obtain a "convex hull" between the chemical potentials, which reveals the stable 0K structures at the vertices of the tie-lines. Further discussions of convex hulls and their application to diverse systems can be found in our previous reports.[22–24]

This concept can be extended to the use of the thermodynamically stable binary phases (placed on the hull, e.g. $Li_3P$ and $Li_2S$) as references when forming the convex hull. This way, one can work with a pseudo-binary (two-dimensional, 2D) convex hull —useful for concentrating on specific tie-lines— rather than a ternary (3D) hull. For lithium thiophosphides, several stable (and metastable) Li-P, Li-S and P-S phases have already been identified, leading to diverse pseudo-binary tie-lines. These tie-lines are used as a guideline for the mixing ratios of the starting binary species, whereby the most investigated one is $Li_2S$-$P_2S_5$. Of these tie-lines, we are particularly interested in $Li_2S$-$Li_3P$ in this study, based on our high-throughput structure prediction endeavours. We thus formed a 2D convex hull for the ternaries on the $Li_2S$-$Li_3P$ tie-line by plotting the $E_f$ / *atom* against the fractional concentration of $Li_3P$ ($x$) in the $[(Li_3P)_x(Li_2S)_{1-x}]$ compound, which is defined as

$x = C_{Li_3P} = \frac{n}{n+m}$ (SB3)

where n and m are the molar amounts of $Li_3P$ and $Li_2S$, respectively, needed to form the ternary compound. Data management for the structures and energies of different Li-P-S ternaries, $Li_2S$ and $Li_3P$ binaries and Li, P and S atomic species, as well as the plotting of the corresponding pseudo-binary phase diagrams were done using our group's *MATADOR* software.[25] XRD patterns were generated from the relaxed geometries using the PyMatgen Python Package.[26]

*Accounting for the Configurational Entropy*

The contribution of structural disorder to the entropy of the system (i.e., configurational entropy, $S_c$) is computed using the following relation

$S_c = k_B T ln(\frac{N!}{n!\,(N-n)!})$ (SB4a)

where $k_B$ is the Boltzmann constant, $T$ temperature, $n$ the number of defect sites to be introduced and $N$ the number of all available sites, at which these $n$ defects can sit. It should be noted that this formulation ($S_c$) does not account for the reduction based on symmetry equivalence, instead considers all possibilities, therefore gives an upper limit for the configurational entropy. One can consider the symmetry operations to filter out the symmetrically-equivalent defect configurations, and thus reduce the configurational space to an irreducible set. Using such an irreducible subset and the degeneracies of each representative configuration one can compute the reduced (degeneracy) entropy ($S_r$) by

$S_r = k_B T ln\, \Omega_m$ (SB4b)

where the degeneracy $\Omega_m$ is the number of symmetrically equivalent defect configurations with identical energy. As we utilise large supercells[27] (as explained below in further details), the total number of possibilities for placing all defects on numerous available sites, e.g. $\binom{27}{9}$ $or$ $\binom{32}{24}$ quickly exceeds the feasible limits. Therefore, we have applied a sequential doping approach to limit the number of possible defect configurations. In such case, total degeneracy entropy can be computed from the individual contributions from each subsequent doping step using $S_r = \sum_i S_i$.

*Ab initio solid-state NMR calculations*

Solid-state $^7$Li, and $^{31}$P NMR chemical shifts were computed using *ab initio* methods for the selected Li-P-S ternary structures. The magnetic response calculations were carried out by applying the gauge-including projector augmented wave (GIPAW) approach.[28,29] GIPAW yields the magnetic shielding tensor ($\sigma$) at each lattice site/nuclear position. However, one needs to convert absolute shielding values into isotropic chemical shifts ($\delta_{iso}$), which are directly relevant to the experiment. This can be done using a reference shielding ($\sigma_{ref}$), as given in Eqn. S5

$\delta_{iso} = \sigma_{ref} + m\sigma_{iso}$   (SB5)

expressed in a general form, where *m* is the gradient, normally assumed equal to −1. To relate the experimental NMR peaks to the predicted shifts, an approach similar to Middlemiss et al.[30] was utilised. In detail, the experimental $\delta_{iso}$ were plotted against the computed $\sigma_{iso}$ values and the *δ-axis* intercept of the best-fit line gives the refence shielding ($\sigma_{ref}$), whereas the slope can be assigned as *m* in **Eqn. SB5**.

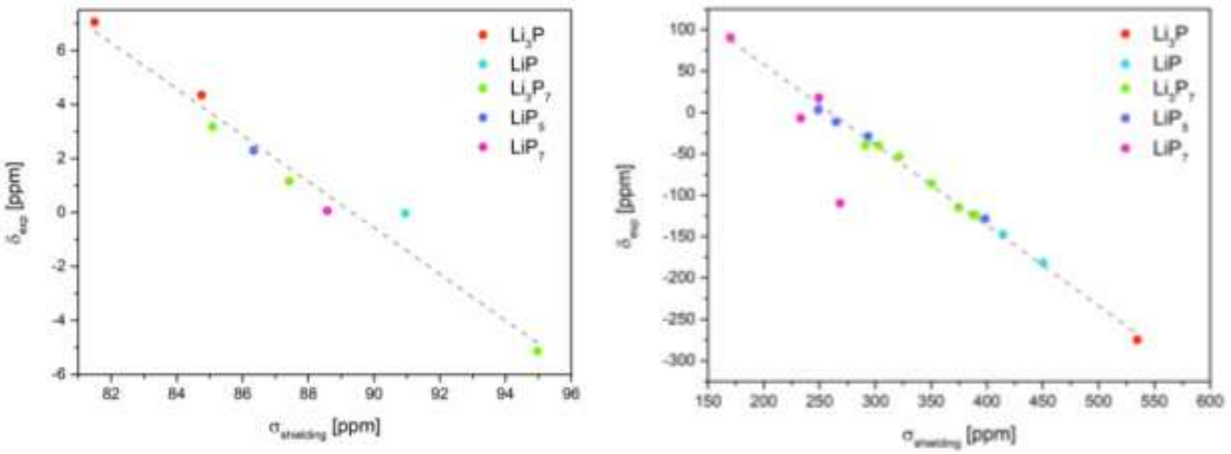


**Supporting Figure SB1.** Predicted isotropic shielding (from DFT/GIPAW calculations) vs. the measured chemical shifts for a selection of related lithium phosphide stoichiometries. The y-intersects of their best fit lines give the reference shifts ($\sigma_{ref}$) for (left) $^7$Li and (right) $^{31}$P NMR spectra, as described in **Eqn SB5**.

Quadrupolar properties were calculated with the GIPAW method utilizing the electric field gradient that is derived directly from the lattice structure and final electron density distribution.[31] GIPAW yields the quadrupolar constant ($C_Q$) and the asymmetry parameters ($\eta_Q$), which are used jointly with the $\delta_{iso}$ values to construct the final $^7$Li, and $^{31}$P NMR spectra with the SOPRANO software,[32] including the 1$^{st}$ and 2$^{nd}$-order quadrupolar effects (e.g. shifts and anisotropy). A Larmor frequency of 700.14 MHz (corresponding to 16.44 T magnetic field) was assumed for $^1$H and the frequencies for the other nuclei were computed from this reference, i.e. 272.12 MHz for $^7$Li and 283.68 MHz for $^{31}$P in all NMR calculations including quadrupolar interactions.

Presented NMR predictions result from DFT calculations involving a finer k-point grid (2π × 0.03 $Å^{-1}$), a higher plane-wave cut-off energy of 1200 eV, and a tighter electronic energy tolerance of 1 x $10^{-11}$ eV to ensure the numerical convergence in the magnetic response. The other DFT parameters/settings were kept the same as described above.

For computing the convoluted NMR spectra, we included the contributions from all defect configurations considered (**see section B2 for details**), by setting their NMR-peak scaling factors to their Boltzmann weights at 298 K (to capture a room temperature weighted low-energy configurations). Configuration thermodynamics/entropy was also taken into account in determining the relative energies for the Boltzmann weights, following the formalism outlined in R Grau-Crespo et al.[27] (which is partly covered in **Eqn. SB4**)

*Predicting the Li-ion Transport Properties using Ab initio Molecular Dynamics (AIMD)*

The ionic bulk conductivity of an electrolyte, i.e. Li-P-S ternary, can be estimated using the Nernst-Einstein relation,[33] defined as

$$\sigma = \frac{ne^2z^2D\,H_R}{kT} \quad \text{(SB6)}$$

where $n$ is the diffusing particle density, $e$ the elementary electron charge, $z$ the ionic charge, $k$ the Boltzmann constant, $T$ the temperature and $D$ the ion diffusivity, and assuming the Haven Ratio ($H_R$) to be unity (i.e. the ionic motion is non-correlated). Self-diffusivity can be calculated from the mean square displacement (MSD) of the Li ions during a molecular dynamics (MD) simulation. This is also known as the tracer diffusivity ($D^*$) and given as

$$D^* \approx \lim_{t\to\infty} \frac{\langle |r_i(t)| \rangle^2}{2dt} = \frac{1}{2dN}\sum_{i=1}^{N}\left(\frac{dr_i(t)}{dt}\right)^2 \quad \text{(SB7)}$$

Where $d$ is the dimensionality of the diffusion (1D, 2D or 3D) and $N$ the number of migrating ions. The MSD of the Li ions in the bulk structure of an electrolyte is given as

$$\langle r_i^2(t) \rangle = \frac{1}{N}\sum_{i=1}^{N}[r_i(t) - r_i(t=0)]^2 \quad \text{(SB8)}$$

Moreover, we estimated the activation energy for a given electrolyte from an Arrhenius plot, i.e. the exponential fit of log(D*) vs. inverse temperature, over a range of temperatures.

To probe the Li-ion conductivity in the Li-P-S ternaries under investigation, we performed *ab initio* molecular dynamics (AIMD) simulations using the Vienna Ab Initio Simulation Package (v. 5.4.4).[34–36] We employed the projector-augmented wave (PAW)[37,38] method jointly with the Perdew-Wang (PW91) version of the Generalised Gradient Approximation (GGA) exchange–correlation potentials.[39] A cut-off-energy for the planewave basis functions of 520 eV was adopted for the cell and geometry relaxations, while the cut-off was lowered to 400 eV for the MD simulations. The atomic positions are fully relaxed to minimize the total energy until it converges within an accuracy of better than 5 meV per cell. A Γ-centred MP grid/mesh with a $2\pi \times 0.05$ Å$^{-1}$ spacing was used to integrate the Brillouin zone for the cell/geometry optimisations, while the MD simulations used only the origin of the reciprocal space (i.e. Γ point). For the MD simulations, we benefitted from the faster implementation in VASP designed for the Γ-only calculations, which proved useful for obtaining numerous adequately long MD trajectories that yield converged MSD and hence diffusivity values. For both cell/geometry optimisations and MD simulations, the normal accuracy setting (*PREC=Normal*) along with an electronic convergence criterion of $10^{-8}$ eV and a Gaussian smearing factor of 0.1 eV were adopted. To allow for unbiased ionic migration dynamics, no symmetry constraints were applied during the MD simulations.

## 2. Preparation of the simulation models for the $Li_2S$:$Li_3P$ solid mixtures (Enumerating the Defect Configurations)

To create the actual models that represent the two solid mixing routes, (Model 1 and Model 2, **Figure 4a**) we started from the cubic anti-fluorite structure of $Li_2S$ obtained from ICSD (Collection code: 54396, $Fm\bar{3}m$, Z=4, containing 8 Li and 4 S sites), relaxed using CASTEP and the DFT settings described earlier (**Part B1**). To reproduce the exact molar P:S ratios in different stoichiometries considered, we adopted two model systems as the starting structures, namely the 2x2x2 conventional supercell (extended version of the ICSD structure, Z=32) and 3x3x3 primitive cell (with Z=27) (main text **Figure 4b**). Here, an extended 3x3x3 primitive cell is used, given the unpractical size of the 3x3x3 conventional supercell (Z=108). In contrast, the model systems are kept sufficiently large in all three directions to minimise any spurious interactions between a dopant atom and its periodic images.

To minimise the computational efforts in the enumeration of different defect configurations, we used only the symmetrically irreducible sets of configurations. Even so, the high number of S sites (27 and 32) that are considered for P substitution give rise to an unmanageable number of different configurations (i.e. $10^4$-$10^7$ possibilities, depending on the template structure) when all defects are placed at once. We therefore had to resort to a sequential doping approach, similar to our previous report.[40] In this automated approach, a pair of P atoms (Models 1 and 2) or vacancies (only Model 2) are introduced on the available S (4a) sites at each doping step, using the three minimum-energy geometries (or the higher-symmetry alternatives, when possible) as the starting point for the consecutive step, and repeated until the desired P/S ratio is reached. Picking multiple configurations at each step increases the diversity in the configuration sampling. The initial $Li_2S$ host structure is used as the template for determining the symmetry equivalence of the sites

in the new geometries obtained at each doping step. It should also be noted that the structures generated through this doping procedure may not be unique (so they are only representative) as other symmetry-equivalent configurations with comparable formation energies usually co-exist.

Once the target molar P/S ratio is achieved through consecutive doping steps, depending on the mixing route choice (viz. only for Model 1), one needs to insert additional Li atoms to the unit cell to obtain the actual stoichiometry as well as to counterbalance the negative charge accumulated in the system. For adding Li atoms at the interstitial (4b) sites, we used the same sequential doping approach, with a pair of Li added at each step. To increase the diversity in our sampling, we also used the *Ab Initio Random Structure Search (AIRSS)* method,[41,42] providing a stochastic approach to determine the Li positions while applying some interatomic distance constraints based on covalent boding radii of Li, P and S atoms. For adequately sampling the configuration space, we considered at least 300 defect configurations for each ternary.

## 3. Discovering New Li-P-S Phases with Stochastic Structure Predictions (AIRSS)

To generate the Li-P-S phase diagram given in the main text (Figure 1), we adopted an approach that combines data mining, prototyping and crystal structure predictions (CSP). To this end, we started by collating the known Li-P-S unary/binary/ternary phase structures from different sources, viz. ICSD,[43] Materials Project (MP),[44] Open Quantum materials (OQMD)[45] databases (plotted in Supporting Figure SB2). As the next step, chemical substitutions (via element swaps) were performed on the relevant structures with similar chemical compositions, again obtained from these sources. This simple structure prototyping is indeed an efficient way of finding new stable and low-energy metastable phases.

To expand our structure collection by identifying novel phases of known stoichiometries as well as new ones, we performed structure searches using the AIRSS method. In our extensive CSP endeavors, we generated around 25000 new structures, primarily focused on the pseudo-binary tie-lines connecting the stable binary systems (i.e. $Li_2S$-$P_2S_5$, $Li_2S$-$Li_3P$, $Li_2S$-LiP, $Li_2S$-$Li_3P_7$, and $Li_2S$-$LiP_7$, as depicted in Supporting Figure SB2). We also performed more directed searches targeting specific stoichiometries if a low-lying phase had been found in the earlier searches.

In a (rather incomplete) attempt to cover the remaining chemical space of the Li-P-S ternary system, we also considered random structures of other stoichiometries ($Li_xP_yS_z$, with {x,y,z} ranging from 1 to 20). One should, however, note that it is computationally very demanding to cover the vast chemical space that is off the boundaries of the stable pseudo-binary tie-lines. More directed structure prediction and/or global optimisation approaches, like USPEX, CALYPSO, GMIN, would possibly be useful to accelerate searches in these compositional ranges. We also note that, considering the limited synthesis possibilities for the resulting phases, we devote only less than 10% of the structure search efforts into these 'off-stable-tie-line' searches, while most of the efforts have gone into exploring the aforementioned stable tie-lines.

## 4. Supporting Tables and Figures

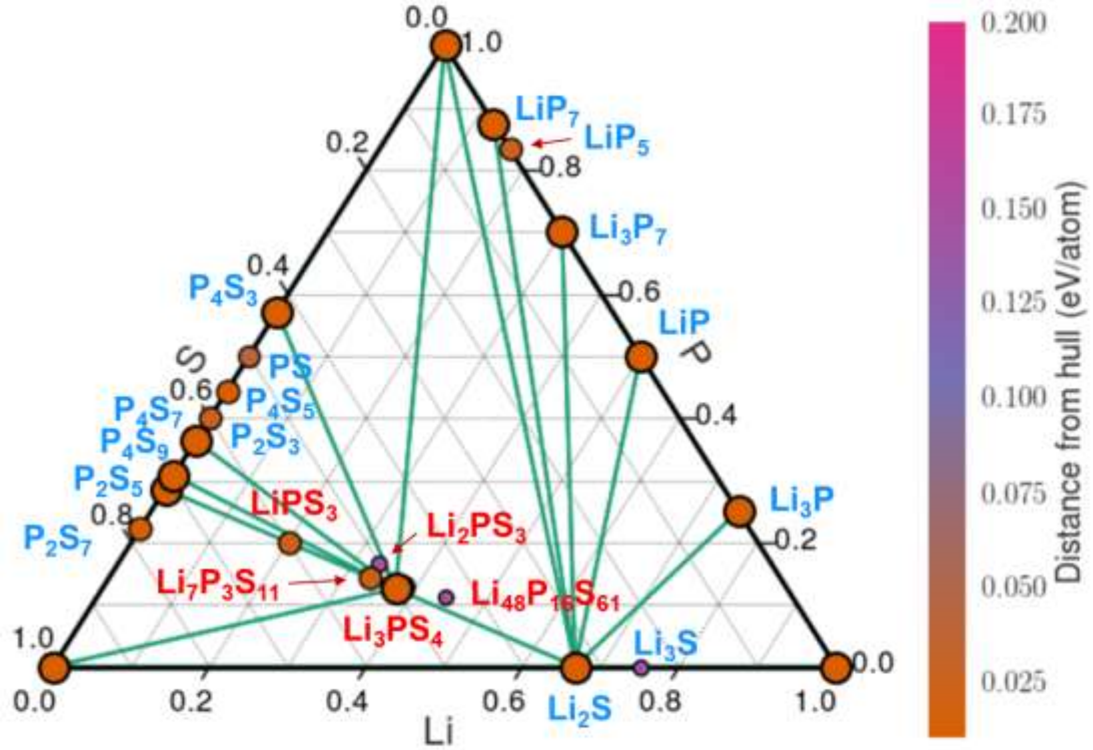


**Supporting Figure SB2.** Computed Li-P-S phase diagram comprising only the structures already available in the literature (using the structures from different sources, viz. ICSD,[43] Materials Project (MP),[44] Open Quantum materials (OQMD)[45] databases. Phase diagram was plotted using MATADOR software[25]

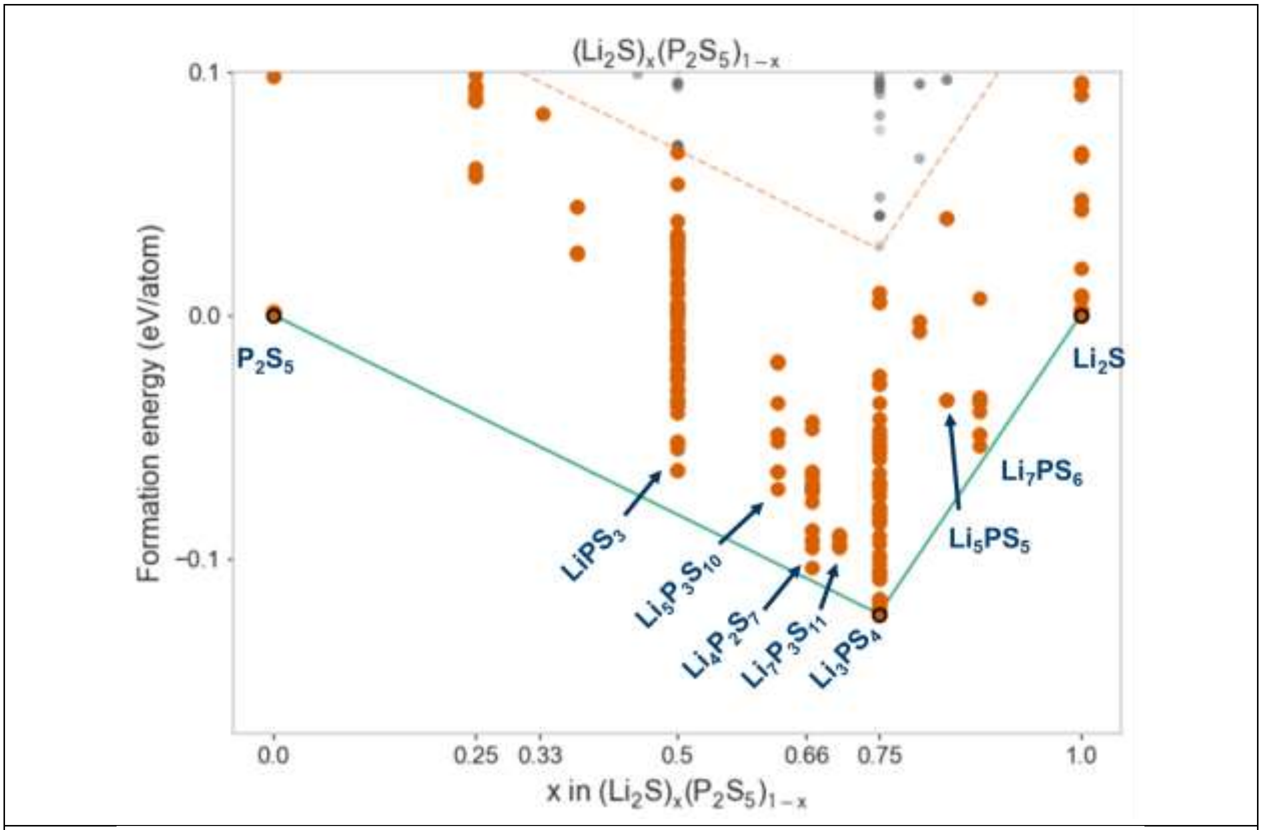

$(Li_2S)_x(P_2S_5)_{1-x}$
Formation energy (eV/atom)
0.1
0.0
−0.1
$P_2S_5$
$Li_2S$
$LiPS_3$
$Li_5P_3S_{10}$
$Li_4P_2S_7$
$Li_7P_3S_{11}$
$Li_3PS_4$
$Li_7PS_6$
$Li_5PS_5$
0.0
0.25
0.33
0.5
0.66
0.75
1.0
x in $(Li_2S)_x(P_2S_5)_{1-x}$


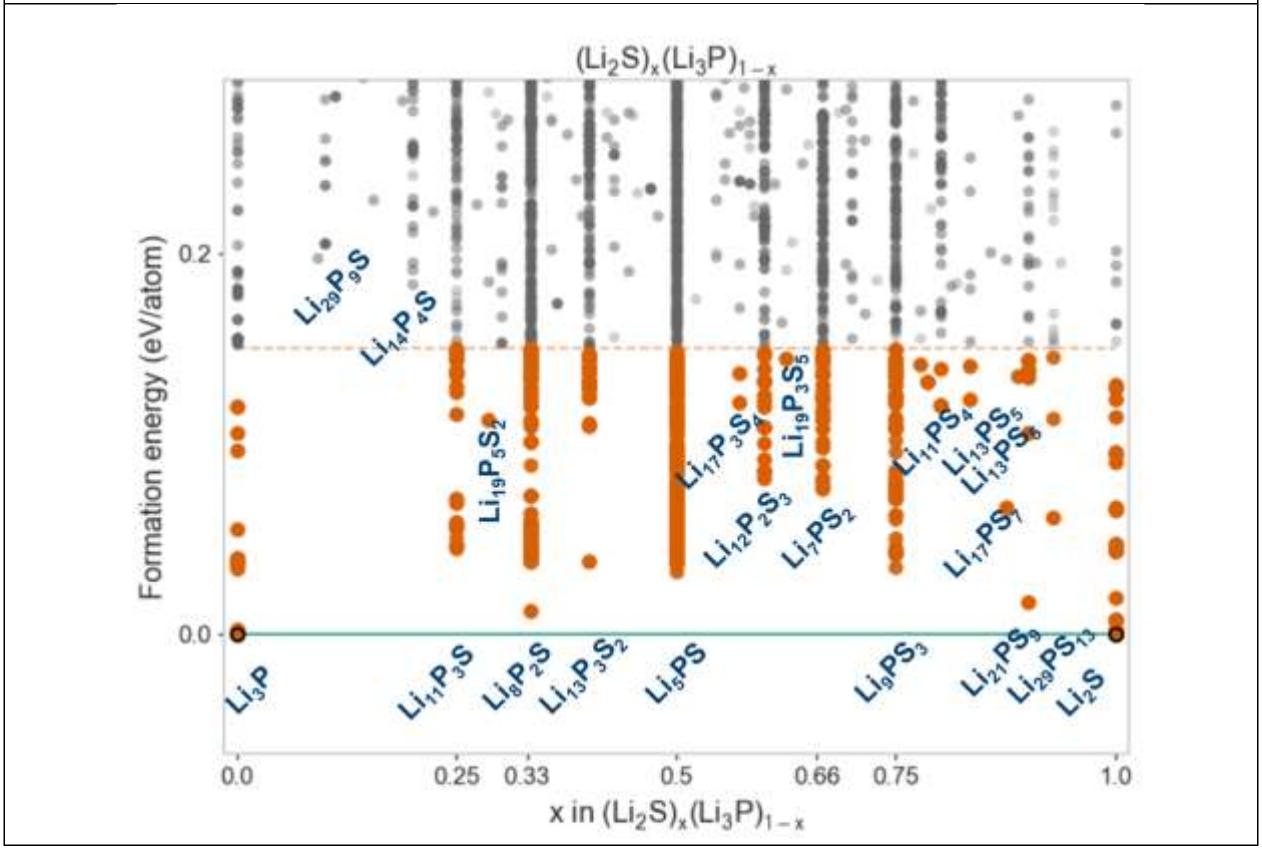

$(Li_2S)_x(Li_3P)_{1-x}$
Formation energy (eV/atom)
0.2
0.0
$Li_{29}P_9S$
$Li_{14}P_4S$
$Li_{19}P_5S_2$
$Li_{17}P_3S_4$
$Li_{19}P_3S_5$
$Li_{12}P_2S_3$
$Li_7PS_2$
$Li_{11}PS_4$
$Li_{13}PS_5$
$Li_{13}PS_6$
$Li_{17}PS_7$
$Li_3P$
$Li_{11}P_3S$
$Li_8P_2S$
$Li_{13}P_3S_2$
$Li_5PS$
$Li_9PS_3$
$Li_{21}PS_9$
$Li_{29}PS_{13}$
$Li_2S$
0.0
0.25
0.33
0.5
0.66
0.75
1.0
x in $(Li_2S)_x(Li_3P)_{1-x}$

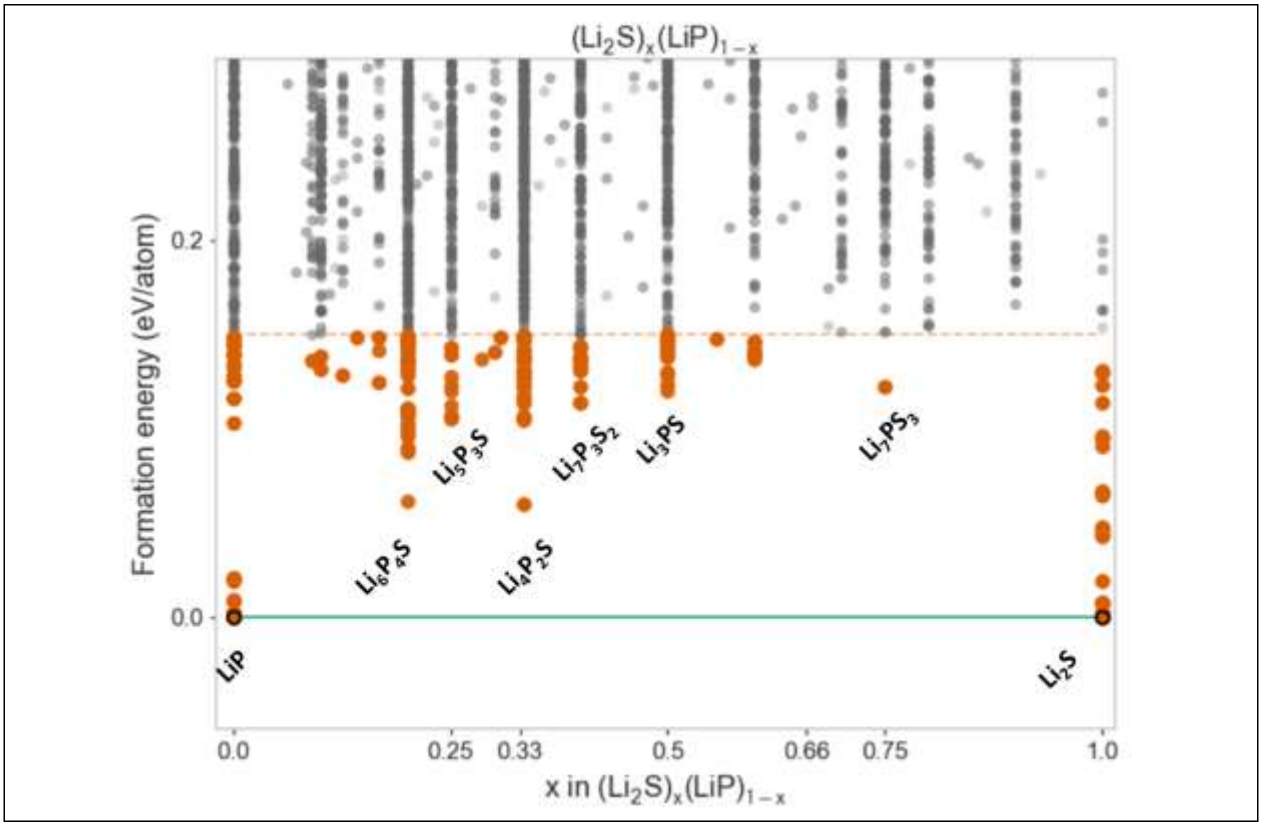


**Supporting Figure SB3.** Pseudo-binary phase diagrams for the $Li_2S$-$P_2S_5$, $Li_2S$-$Li_3P$ and $Li_2S$-LiP tie-lines, as computed using the DFT/PBE stochastic structure searches. The orange dashed line depicts the 150 meV/atom border (which gives an indication as to which phases are thermodynamically accessible at ambient temperature).

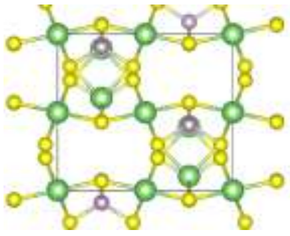

γ-$Li_3PS_4$ ($Pmn2_1$)
SWAPS and ICSD 180318
ΔE=0 meV/atom

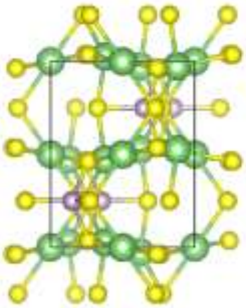

β-$Li_3PS_4$ (Pnma)
ICSD 180319
ΔE=2.6 meV/atom

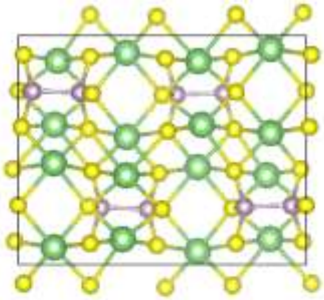

$Li_2PS_3$ ($P2_12_12_1$)
SWAPS / AgPS 35628
ΔE=0.0 meV/atom

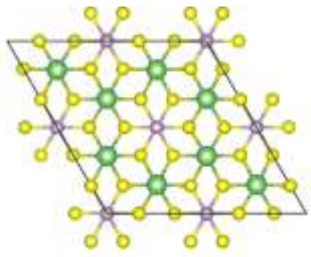

$Li_2PS_3$ (Cm)
ICSD 33506
ΔE=2.7 meV/atom

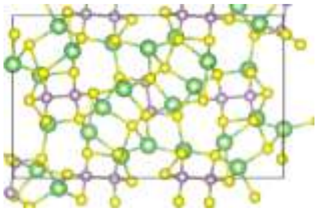

$Li_2PS_3$ (P21/c)
ICSD 33506
ΔE=18.2 meV/atom

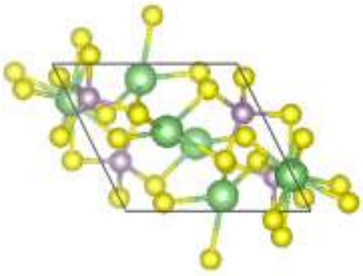

$Li_4P_2S_7$ (P-1)
MP / (ICSD 655288)
ΔE= 0.9 meV/atom

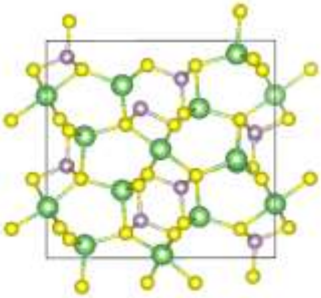

$Li_{48}P_{16}S_{61}$ (Pm)
AFlow
ΔE=21 meV/atom

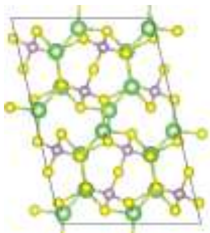

$Li_4P_2S_7$ (C2/c)
SWAPS / AgPS 949
ΔE= 11.7 meV/atom

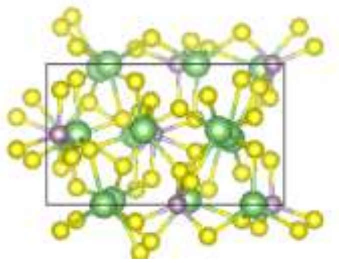

$Li_4P_2S_7$ ($P2_12_12_1$)
SWAPS / NaPO 10370
ΔE= 15.9 meV/atom

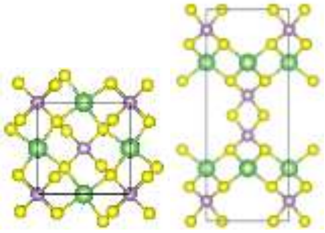

$LiPS_3$(P42/mnm)
SWAPS / CuPS 430200
ΔE=4.3 meV/atom

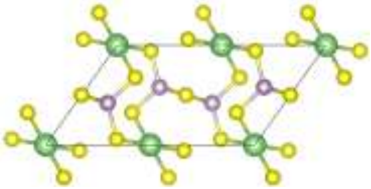

$LiPS_3$(C2/m)
Janek et al.[46]
ΔE=13.2 meV/atom

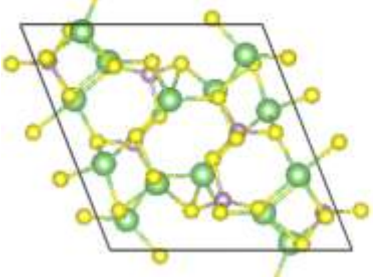

$Li_7P_3S_{11}$(P-1)
ICSD 157654
ΔE=16.4 meV/atom

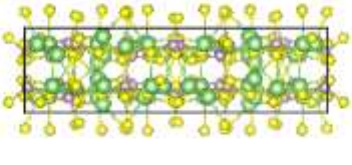

$Li_7P_3S_{11}$(C2/c)
SWAPS / AgPS 414335
ΔE=20.3 meV/atom

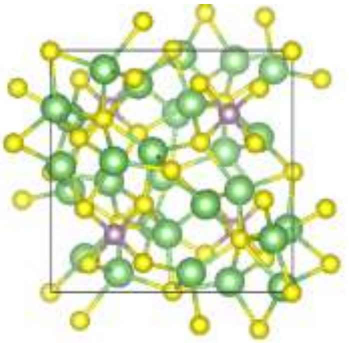

β-$Li_7PS_6$ ($P2_13$)
SWAPS / CuPSe 628648
ΔE=16.7 meV/atom

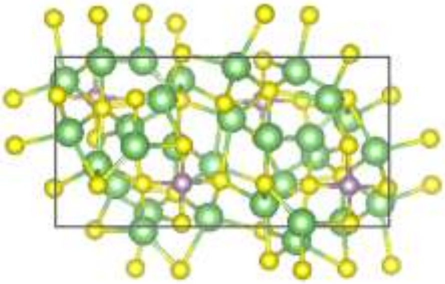

α-$Li_7PS_6$ ($Pna2_1$)
SWAPS / CuPSe 280411
ΔE=36.0 meV/atom

**Supporting Figure SB4.** Visualisation of the known crystal structures obtained from publications, as well as  and AFlow, Materials Project and ICSD databases Distance to hull (ΔE, in meV/atom) and space group symmetry information are also reported for each phase. The provenance of the structures are also indicated, whereby 'SWAPS' corresponds to the structure obtained by prototyping (i.e. element swapping).

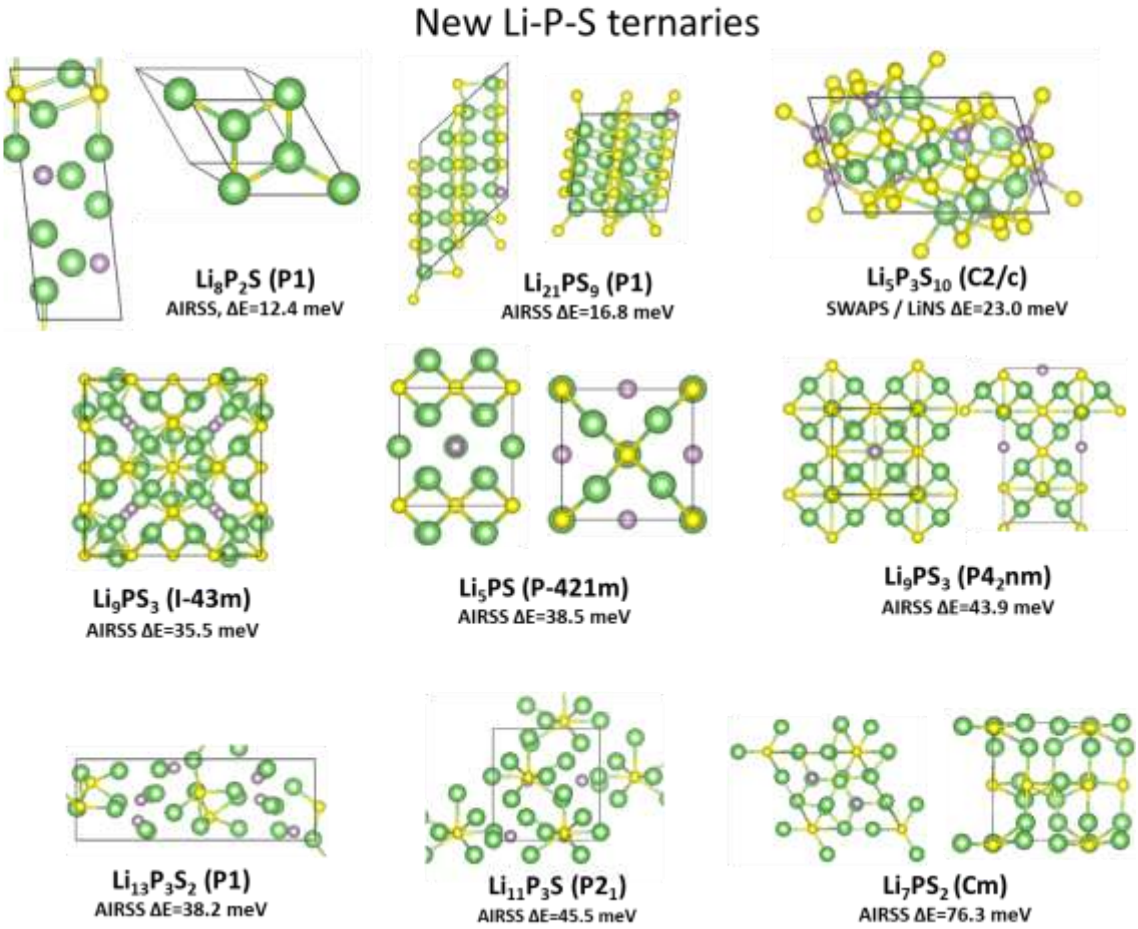


**Supporting Figure SB5.** Visualisation of the new crystal structures discovered during the stochastic structure searches. Source of the structures (i.e., AIRSS or atomic swaps), distance to hull (ΔE, in meV/atom) and space group symmetry information is also reported for each phase.

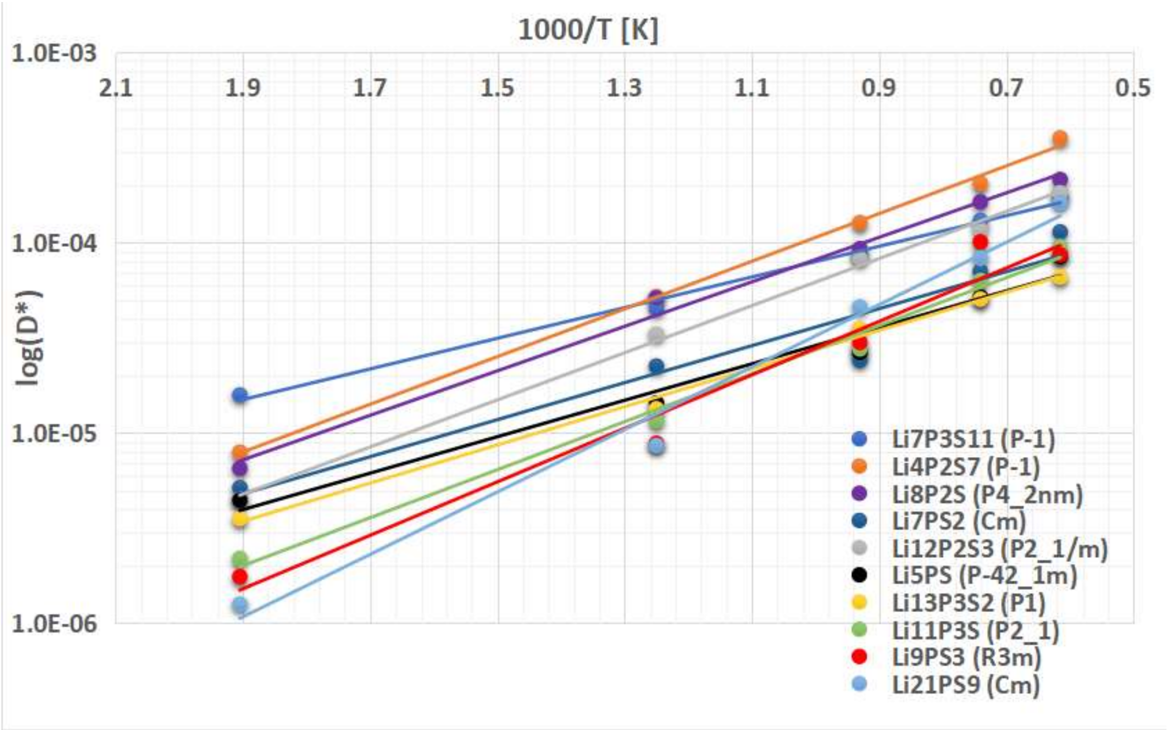


**Supporting Figure SB6.** Arrhenius-type plots (logarithm of computed diffusivity vs. inverse temperature) of various Li-P-S ternary phases computed using AIMD simulations at a temperature range of 525-1625K with 275K steps. Activation energies can be extracted from the Arrhenius equation using the exponential term of the best fit slope. The highest room-temperature conductivity is predicted for the $Li_7P_3S_{11}$ (P-1) phase. The newly found $Li_8P_2S$ is also predicted to have a good RT conductivity.

**Supporting Table SB1.** Selected stable and low-energy metastable Li-P-S phases lying within 150 meV/atom of the ternary hull (with respect to the pure Li/P/S phases), as computed at the DFT/PBE level. Both the known phases from different sources (literature or ICSD/OQMD/AFLOW/Material Project (MP)) and the newly identified ones (from AIRSS and elemental substitutions, SWAPS) are reported here.

| Stoichiometry | Hull dist [meV/atom] | Space group | Volume/fu | Structure origin | Description/comments |
|---|---|---|---|---|---|
| **Known ternaries** | | | | | |
| γ-$Li_3PS_4$ | 0 | $Pmn2_1$ | 160.8 | SWAPS/CuPSe 95412<br>SWAPS/ AgPS 656978<br>SWAPS/LiVO 257863<br>SWAPS/CaSiO 24452 | All the elemental swaps starting from different structures with different compositions (of the same space group symmetry) give the same $Li_3PS_4$ structure. |
| β-$Li_3PS_4$ | 2.6 | Pnma | 167.4 | ICSD 180319 | modified, Li3 sites were removed. |
| $Li_2PS_3$ | 0 | $P2_12_12_1$ | 112.9 | SWAPS/ AgPS 35628 | |

| | | | | | |
|---|---|---|---|---|---|
| | 2.7 | Cm | 107.0 | ICSD 33506 | Experimental reference is P63/mcm (33506) which has higher phosphorous content (i.e. $Li_2P_2S_3$). So two P sites were deleted. |
| | 18.2 | $P2_1/c$ | 122.8 | SWAPS / AgPS 24782 | |
| $Li_4P_2S_7$ | 0.9 | P-1 | 322.1 | MP (ICSD 655288) | |
| | 8.9 | P-1 | 269.5 | MP / SWAPS / AgPS 949 | |
| | 11.7 | C2/c | 271.1 | SWAPS / AgPS 949 | |
| | 15.9 | $P2_12_12_1$ | 270.6 | SWAPS / NaPO 10370 | |
| | 27.6 | $P2_1/c$ | 281.9 | SWAPS / LiPO 248415 | |
| | 31.6 | P-1 | 296.4 | SWAPS / LiPO 246859 | |
| $LiPS_3$ / $Li_2P_2S_6$ | 4.3 | $P4_2/mnm$ | 127.7 | SWAPS / CuPS 430200 | A new more stable phase was found by swapping from a CuPS structure. |
| | 13.2 | C2/m | 109.3 | From Janek et al.[46] | $Li_2P_2S_6$ structure from Janek et al. |
| $Li_7P_3S_{11}$ | 16.2 | P-1 | 437.0 | ICSD 157654 | |
| | 20.3 | C2/c | 429.3 | SWAPS/ AgPS 414335 | |
| β-$Li_7PS_6$ | 16.7 | $P2_13$ | 250.3 | SWAPS/ CuPSe / 628648 | |
| | 21.3 | $P2_13$ | 249.5 | SWAPS/ AgPSe / 54055 | |
| α-$Li_7PS_6$ | 36.0 | Pna21 | 257.6 | SWAPS/ CuPSe 280411 | |
| $Li_{48}P_{16}S_{61}$ | 22.6 | Pm | 2667.1 | AFLOW | Li48P16S61, a computational structure |
| **<u>New Ternaries</u>** | | | | | |
| $Li_8P_2S$ | 12.4 | P1 | 166.5 | AIRSS | **On $Li_2S$-$Li_3P$ line.** |
| | 63.4 | P1 | 173.3 | AIRSS | |
| | 72.7 | Cm | 155.9 | AIRSS | |
| | 101.1 | Cm | 185.4 | AIRSS | |
| | 109.0 | $Pmc2_1$ | 207.9 | AIRSS | |
| $Li_{21}PS_9$ | 16.8 | P1 | 482.8 | AIRSS | **On $Li_2S$-$Li_3P$ line.** |
| | 134.8 | Cm | 529.7 | AIRSS | |
| $Li_5P_3S_{10}$ | 23.0 | C2/c | 372.1 | SWAPS / CaSiO 16654 | **On $Li_2S$-$P_2S_5$ line.** |
| | 58.0 | $P2_12_12_1$ | 405.3 | AIRSS | |

| | | | | | |
|---|---|---|---|---|---|
| $Li_{13}P_3S_2$ | 38.2 | P1 | 264.3 | AIRSS | **On $Li_2S$-$Li_3P$ line.** |
| | 124.1 | Cmc2_1 | 341.0 | AIRSS | |
| $Li_5PS$ | 33.0 | P2 | 104.4 | AIRSS | **On $Li_2S$-$Li_3P$ line.** |
| | 37.0 | Pmn2_1 | 102.0 | AIRSS | |
| | 38.5 | P-42$_1$m | 103.1 | AIRSS | |
| | 40.0 | P4bm | 102.9 | AIRSS | |
| | 42.5 | P4/nmm | 102.2 | AIRSS | |
| $Li_9PS_3$ | 35.5 | I-43m | 207.9 | AIRSS | **On $Li_2S$-$Li_3P$ line.** |
| | 43.9 | P4$_2$nm | 197.4 | AIRSS | |
| | 78.9 | R3m | 203.9 | SWAPS / LiNS 240749 | |
| $Li_{11}P_3S$ | 45.5 | P2$_1$ | 232.7 | AIRSS | **On $Li_2S$-$Li_3P$ line.** |
| | 46.7 | P4$_2$cm | 215.7 | AIRSS | |
| | 47.2 | P4$_2$nm | 213.6 | AIRSS | |
| | 50.4 | Fmm2 | 210.9 | AIRSS | |
| and several more | | | | | |
| $Li_5PS_5$ | 54.4 | Pbcm | 208.7 | SWAPS / NaAsO 411721 | **On $Li_2S$-$P_2S_5$ line.** |
| | 129.2 | Cm | 239.0 | AIRSS | |
| $Li_2PS_4$ | 52.3 | P2$_1$/c | 151.2 | SWAPS / CaSiO 421708 | **Not on any line. Close to $Li_2S$-$P_2S_5$** |
| | 56.7 | P1 | 149.0 | AIRSS | |
| | 57.5 | P2$_1$/c | 150.7 | SWAPS / CaSiO 245078 | |
| $Li_3PS_3$ | 57.4 | Pna2$_1$ | 131.5 | SWAPS / LiAsS 424835 | **Not on any line (close to $Li_2S$-$P_2S_5$)** |
| | 57.9 | P1 | 131.7 | SWAPS / LiAsS 59381 | |
| $Li_4P_2S$ | 59.9 | Cm | 128.7 | AIRSS | **On $Li_2S$-LiP line.** |
| $Li_{29}PS_{13}$ | 61.7 | P1 | 686.6 | AIRSS | **On $Li_2S$-$Li_3P$ line.** |
| $Li_4P_4S_3$ | 61.9 | P2$_1$/c | 216.0 | SWAPS/ CuPSe 430941 | **Not on any line.** |
| $Li_{12}P_4S_{15}$ | 62.6 | P1 | 639 | AIRSS | **Not on any line (close to $Li_2S$-$P_2S_5$)** |
| $Li_5P_2S_8$ | 67.1 | P1 | 322.4 | SWAPS / LiPO 31797 | **Not on any line (close to $Li_2S$-$P_2S_5$)** |
| $LiP_2S$ | 66.4 | Pbca | 73.6 | SWAPS/ CuPSe 430942 | **Not on any line.** |
| $Li_{11}P_4S_{16}$ | 70.6 | P-1 | 651 | AIRSS | **Not on any line (close to $Li_2S$-$P_2S_5$)** |
| $Li_3P_5S_{14}$ | 71.3 | P1 | 590.6 | SWAPS/KVO 420851 | **On $Li_2S$-$P_2S_5$ line.** |
| $Li_2P_4S_{13}$ | 77.6 | P1 | 566.6 | AIRSS | **Not on any line. Close to $Li_2S$-$P_2S_5$** |
| $LiP_3S_5$ | 77.8 | P1 | 230.7 | AIRSS | **Not on any line.** |

| | | | | | |
|---|---|---|---|---|---|
| $Li_4P_3S_4$ | 79.3 | P-1 | 231.3 | SWAPS/ CuPSe 430940 | **Not on any line.** |
| $Li_7PS_2$ | 76.3 | Cm | 167.9 | AIRSS | **On $Li_2S$-$Li_3P$ line.** |
| | 79.6 | Cm | 167.1 | AIRSS | |
| $Li_9P_4S_{12}$ | 82.9 | P-1 | 532.9 | SWAPS/ CuPSe 420939 | **Not on any line (close to $Li_2S$-$P_2S_5$)** |
| $Li_{12}P_2S_3$ | 82.4 | Cm | 313.4 | AIRSS | **On $Li_2S$-$Li_3P$ line.** |
| | 85.9 | P1 | 311.9 | | |
| | 119.6 | $P2_1$ | 306.2 | AIRSS | |
| $Li_8P_5S_{18}$ | 92.9 | Pbcn | 645.1 | SWAPS / CaSiO 10039 | **Not on any line. Close to $Li_2S$-$P_2S_5$** |
| $Li_3P_2S_7$ | 83.1 | Ima2 | 249.8 | SWAPS / CaSiO 424472 | **Not on any line. Close to $Li_2S$-$P_2S_5$** |
| | 98.7 | $P2_1/c$ | 252.7 | SWAPS / CaSiO 2282 | |
| $LiPS_2$ | 94.7 | Cc | 76.3 | SWAPS / LiAsSe 248116 | **Not on any line. Close to $Li_2S$-$P_2S_5$** |
| $Li_6P_4S$ | 61.9 | P1 | 204.4 | AIRSS | **On $Li_2S$-LiP line.** |
| | 87.7 | Cm | 189.7 | AIRSS | |
| | 99.3 | $Cmc2_1$ | 183.5 | AIRSS | |
| $Li_8P_2S_9$ | 100.9 | $P2_1/c$ | 473.3 | SWAPS / HPO 2096 | **On $Li_2S$-$P_2S_5$ line.** |
| $Li_3PS$ | 119.9 | C2 | 88.1 | AIRSS | **On $Li_2S$-LiP line.** |
| | 127.7 | Cm | 89.6 | | |

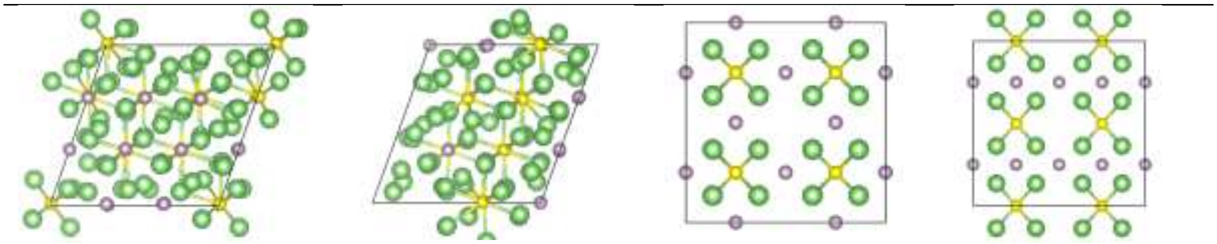

| top view | side view | top view | side view |
|---|---|---|---|
| Model 1 – $E_f$ =39.1 meV/atom (P1) | | Model 2 – $E_f$ = 149.1 meV/atom (P4/mmm) | |
| $Li_{2+2/3}(P_{2/3}S_{1/3})_1$ | | $Li_2(P_{0.5}S_{0.25}\square_{0.25})_1$ | |

**Supporting Figure SB7.** Comparison of the lowest-energy structures from the two solid-mixing Models considered for obtaining the $Li_8P_2S$ ternary (2$Li_3P$ + 1$Li_2S$ mixing). Colour code: Li: light green, P: purple, S: yellow.

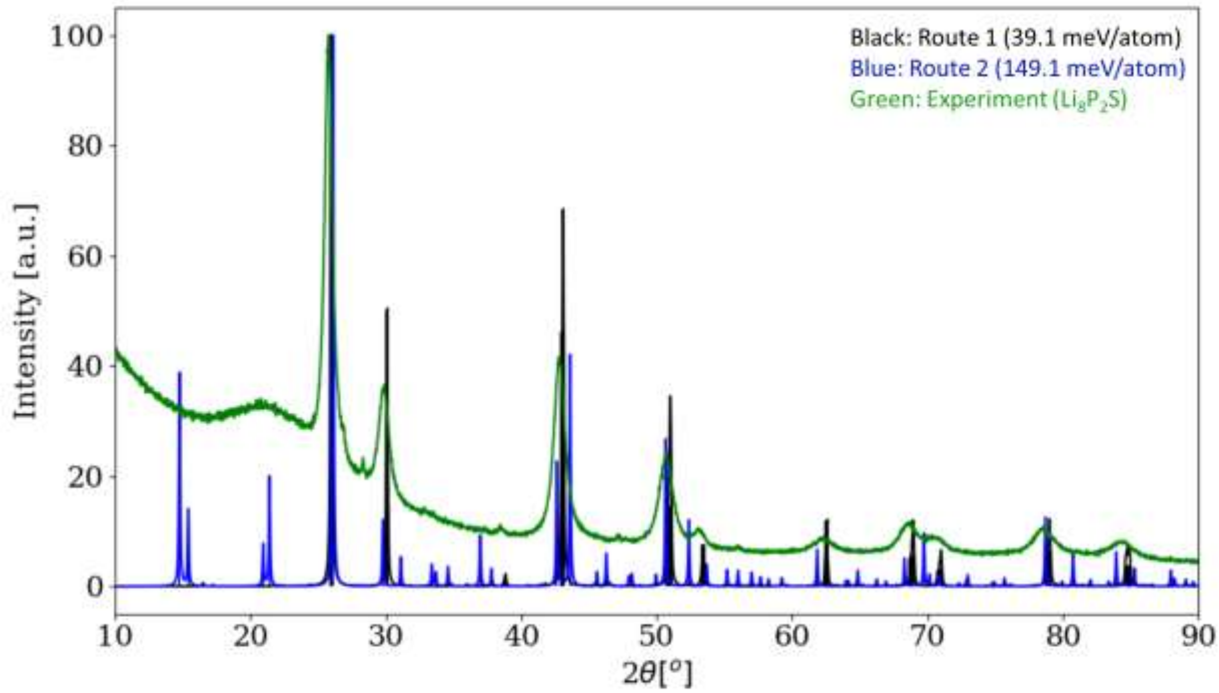


**Supporting Figure SB8.** $Li_8P_2S$ XRD patterns computed using the lowest-energy structures from the two solid-mixing routes/models (Route 1: black, Route 2, blue) compared with the experimental reference (green).

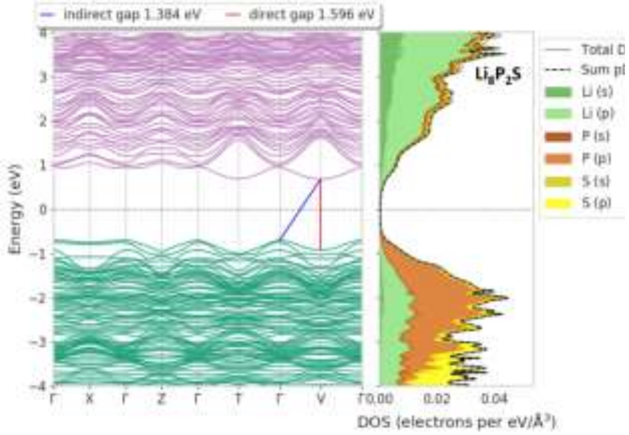


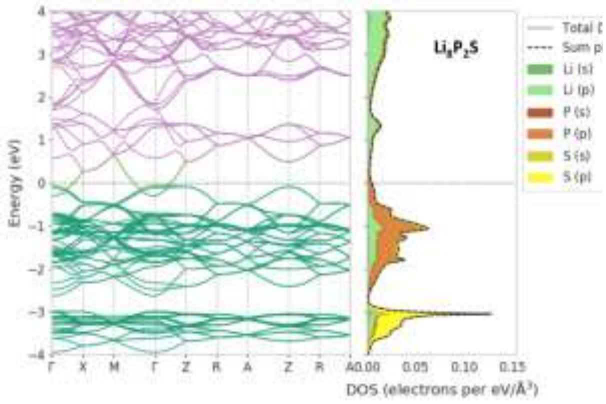


| | |
|---|---|
| Model 1 – $E_f$ =39.1 meV/atom (P1) | Model 2 – $E_f$ = 149.1 meV/atom (P4/mmm) |
| $Li_{2+2/3}(P_{2/3}S_{1/3})_1$ | $Li_2(P_{0.5}S_{0.25}\square_{0.25})_1$ |

**Supporting Figure SB9**. Electronic density of states (DOS) and band structures of $Li_8P_2S$ from Models 1 and 2. Even though the DOS and band structures are only shown for the lowest-energy configurations, these characteristic findings are also valid for other low-lying configurations from Models 1 and 2.

**Supporting Table SB2.** The computations for the configuration entropy for the new Li-P-S ternaries.

| Ternary | System size | N, n | $S_c$ [eV/K/atom][a] | $S_r$ [eV/K/atom][b] |
|---|---|---|---|---|
| $Li_7PS_2$ (Model 1) | 3x3x3 prim. cell (81 atoms) | 27 S(4a) sites, 9 P to add 27 vacant (4b) site, 9 Li to add | 2 x 1.63 x $10^{-5}$ | 7.83 x $10^{-6}$ |
| $Li_8P_2S$ (Model 1) | 3x3x3 prim. cell (81 atoms) | 27 S(4a) sites, 18 P to add 27 interstices (4b), 18 Li to add | 2 x 1.63 x $10^{-5}$ | 7.87 x $10^{-6}$ |
| $Li_8P_2S$ (Model 2) | 2x2x2 super cell (96 atoms) | 32 S(4a) sites, 16 P and 8 vacancy sites to add | 1.45 x $10^{-5}$ (all at once) or 1.81 x $10^{-5}$ + 1.70 x $10^{-5}$ (sequential) | 6.36 x $10^{-6}$ |
| $Li_{11}P_3S$ (Model 1) | 2x2x2 super cell (96 atoms) | 32 S(4a) site, 24 P sites to add 32 interstices (4b), 24 Li to add | 2 x 1.45 x $10^{-5}$ | 6.75 x $10^{-6}$ |
| $Li_5PS$ (Model 1) | 2x2x2 super cell (96 atoms) | 32 S(4a) site, 16 P sites to add | 1.81 x $10^{-5}$ | 6.85 x $10^{-6}$ |

(a) Using Eqn. 4a; (b) Using Eqn. 4b;

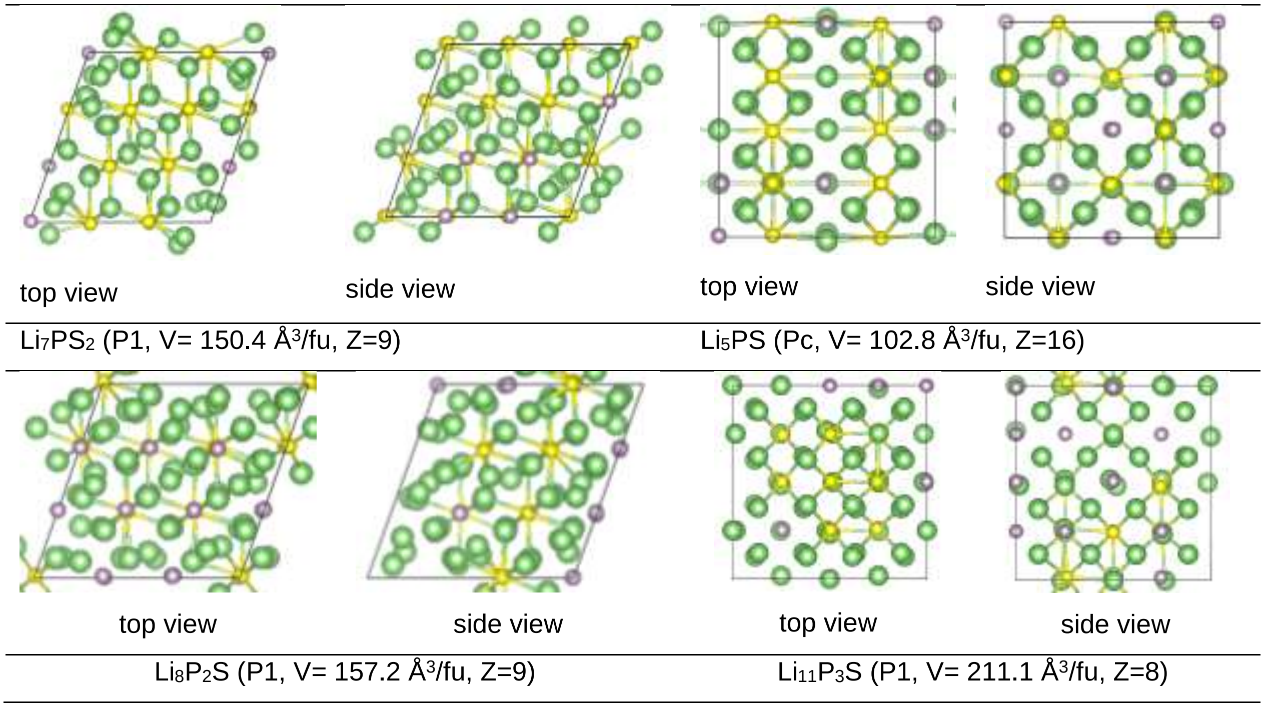


**Supporting Figure SB10.** DFT-optimised structures for the lowest-energy configurations for the solid Li-P-S ternary solutions obtained from the mixing Model 1.

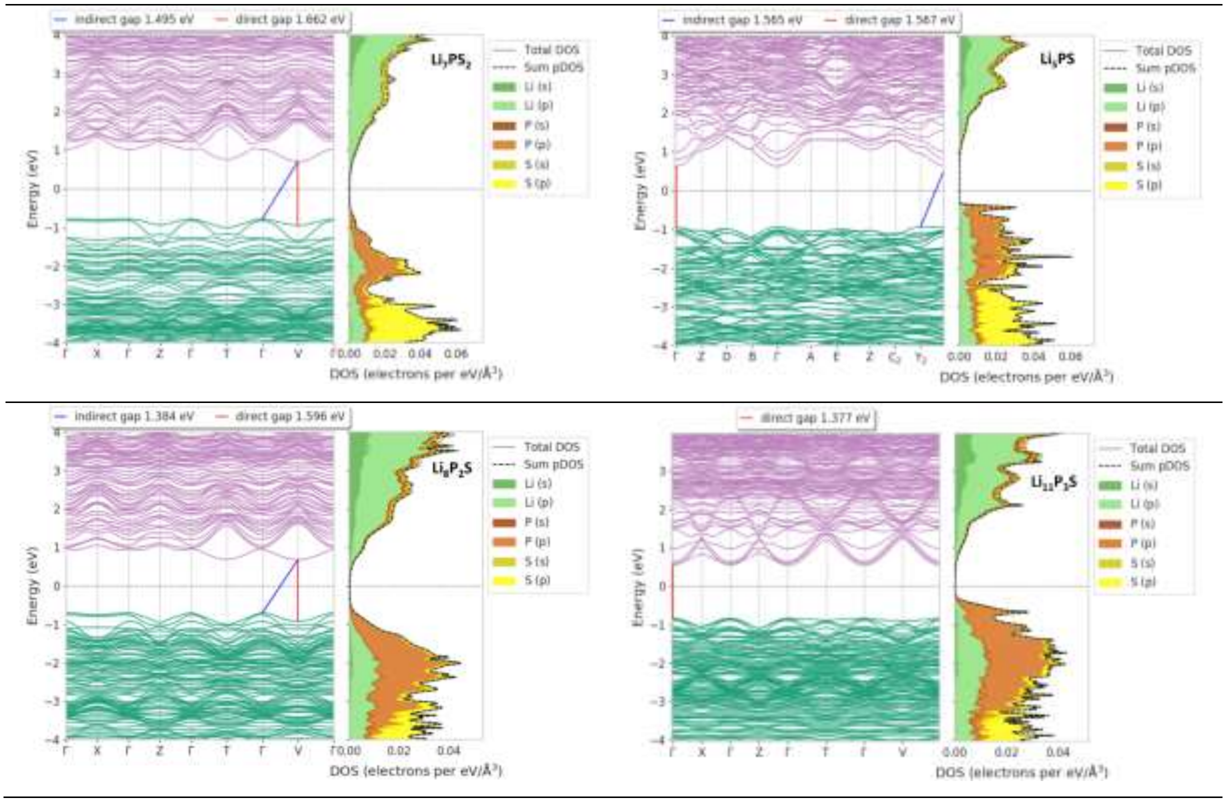


**Supporting Figure SB11.** Electronic density of states (eDOS) and band structures of the lowest-energy configurations for each Li-P-S ternary and overlay of the eDOS of all four ternaries. All new electrolytes are clearly electronic insulators given their large direct bandgaps.

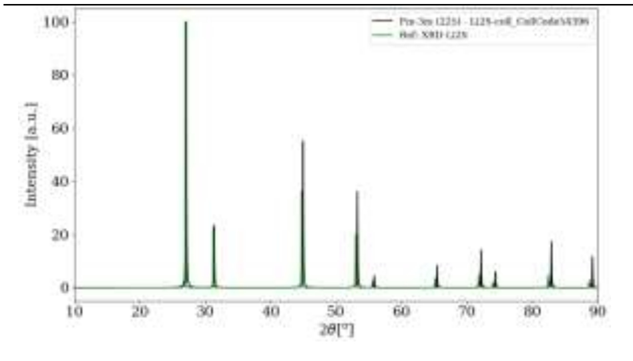

$Li_2S$ ($Fm\bar{3}m$)

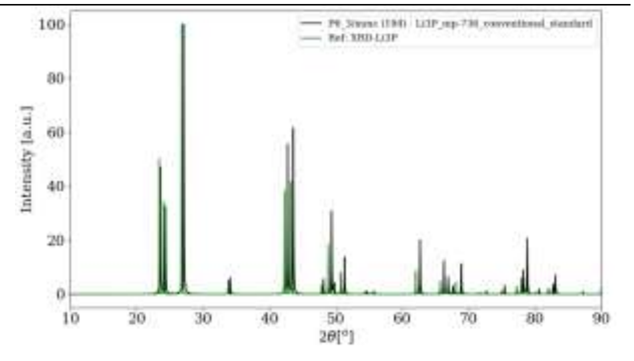

$Li_3P$ ($P6_3/mmc$)

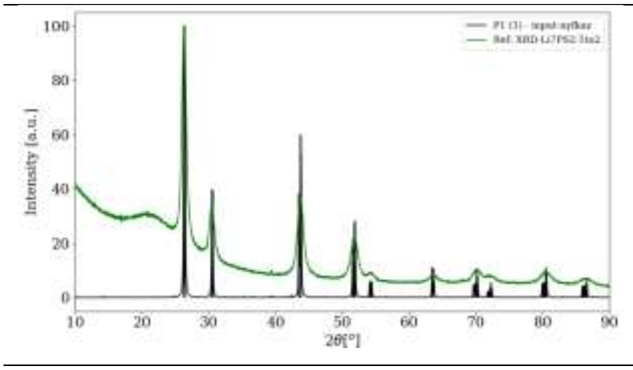

$Li_7PS_2$ (P1)

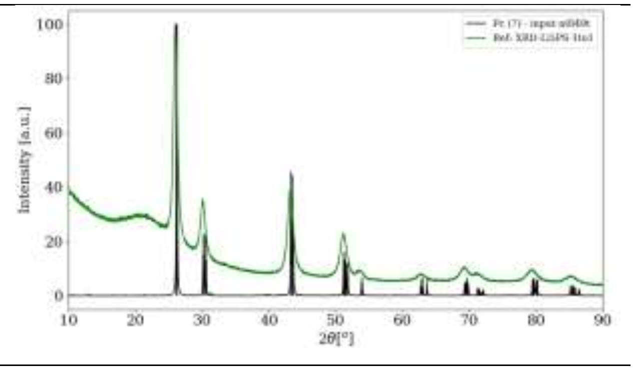

$Li_5PS$ (Pc)

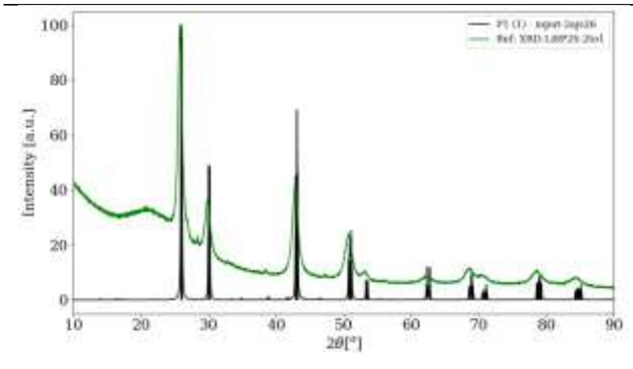

$Li_8P_2S$ (P1)

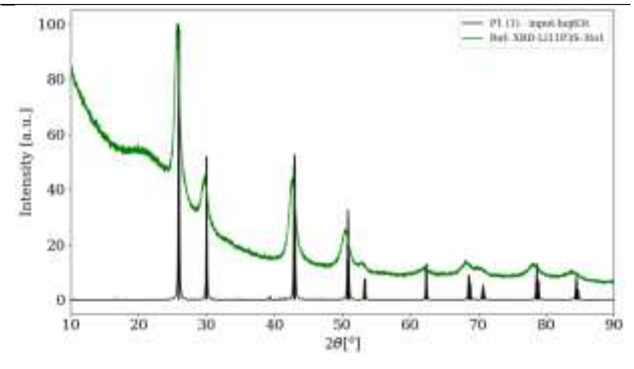

$Li_{11}P_3S$ (P1)

**Supporting Figure SB12.** Comparison of the computed XRD patterns (black) with the corresponding experimental references (green).

.

**Supporting Table SB3.** Computed hull distances (ΔE), ionic conductivities ($\sigma_{Li+}$) and activation energies ($E_a$) of the new and other common Li-P-S ternaries (from the $Li_2S$-$P_2S_5$ tie-line) as computed from ab initio molecular dynamics (AIMD) simulations and compared to the experimental measurements.

| Material | ΔE [meV/at] | Comp. $E_a$ [meV] | Exp. $E_a$ [meV] | Comp. $\sigma_{Li^+}$ [S/cm] | Exp. $\sigma_{Li^+}$ [S/cm] |
|---|---|---|---|---|---|
| <u>$Li_2S$-$P_2S_5$ tie-line</u> | | | | | |
| $Li_7P_3S_{11}$ ($P\bar{2}$) | 16.2 | 160* <br> 150*/190[47]c,d | 120[48] <br> 170[49] | 7.8 x $10^{-1}$ (525 K) * | 3.2 × $10^{-3}$ (RT)[48] <br> 1.7 × $10^{-2}$ (RT)[49] |
| β-$Li_3PS_4$ ($Pnma$) | 2.6 | 337a* | 155[50]/240[51] <br> 220-490[52–57] | 9.6 x $10^{-2}$ (525 K)b* <br> 1.8 (800 K) * | 3.0 × $10^{-2}$ (500 K)[50] <br> 1.0 × $10^{-4}$ (RT)[51] |
| γ-$Li_3PS_4$ ($Pmn2_1$) | 0.0 | 710*/372f*/ <br> 200[47]c | 213[50]/490[53] | 3.3 x $10^{-4}$ (525 K)b <br> 1.4 (800 K) | 3.0 × $10^{-7}$ (RT)[50] |
| <u>$Li_2S$-$Li_3P$ tie-line</u> | | | | | |
| $Li_2S$ (x=0.0) | 0.0 | 800*/390[47]c <br> 450[58]c/950[59]c | ca. 750[60] | 1.3 x $10^{-3}$ (525K) b <br> 1.9 x $10^{-1}$ (1075K) | $10^{-14}$ - $10^{-10}$ (RT) [60,61] <br> $10^{-7}$ (RT, nano films)[61] <br> 2.6 × $10^{-2}$ (1000 K)[62]g <br> 1.3 × $10^{-1}$ (1170 K)[62]g |
| $Li_7PS_2$ (x=0.33) | 41.0 | 275-309* | 204e | 8.5 x $10^{-2}$ to 2.0 x $10^{-1}$ (525 K) | 1.25 × $10^{-5}$ (RT, EIS)e |
| $Li_5PS$ (x=0.50) | 39.8 | 289-353* | 140−170h; 171e* | 1.9 x $10^{-1}$ to 6.4 x $10^{-1}$ (525 K) | 6.26 × $10^{-5}$ (RT, EIS)e |
| $Li_8P_2S$ (x=0.67) | 39.1 | 192-230* | 110−150h; 154e | 3.6 x $10^{-1}$ to 3.9 x $10^{-1}$ (525 K) | 1.09 × $10^{-4}$ (RT, EIS) e |
| $Li_{11}P_3S$ (x=0.75) | 31.4 | 156-231* | 140−150h; 164e* | 9.4 x $10^{-2}$ to 7.5 x $10^{-1}$ (525 K) | 3.19 × $10^{-4}$ (RT, EIS) e |
| $Li_3P$ (x=1.0) | 0.0 | 696* | 180[63], 530[64] | 5.7 x $10^{-4}$ (525 K)b <br> 3.2 x $10^{-1}$ (1075 K) | 6.6 × $10^{-4}$ (RT)[63] <br> 3.0 × $10^{-8}$ (50 °C)[64] |

*Current work; (a)For the nano-porous β-$Li_3PS_4$ model, we deleted all the Li3 site atoms, assigning the Li2 sites with full occupancy (following from a previous study for an analogous thiophosphide, $Na_3PS_4$ (see ref.[65]); (b)These calculated diffusivity values are based on non-converged MSD trends, due to the low $Li^+$ diffusivity of the electrolyte at the given temperature. (c)Using nudged-elastic-band (NEB) calculations; (d)Using Arrhenius plots from AIMD simulations; (e) measured using impedance spectroscopy in this study; (f) values correspond to the fully lithiated and under-lithiated γ-$Li_3PS_4$ cases.; (g) converted from the reported diffusivity D=1.39 × $10^{-5}$ cm$^2$/s using the Nernst-Einstein relation (Eqn. S6). (h) measured by NMR relaxometry.

## 5. Li-ion transport simulations using AIMD

Supporting Table SB3 reveals the overall agreement between our $\sigma_{Li^+}$ and $E_a$ values computed for the reference materials ($Li_7P_3S_{11}$, $Li_3PS_4$, $Li_2S$ and $Li_3P$) and the previous computational and experimental reports. Of these, γ-$Li_3PS_4$ yields the values with the largest deviation from the reported values, possibly due to the lack of Li vacancies in our model, which are included in other models.[47] Relevantly, we performed a series of MD simulations, which revealed an increasing Li diffusivity with the increasing amount of Li vacancies (i.e. γ-$Li_{3-x}PS_4$, x=0-0.5, Supporting Figure S), in line with a previous report on a similar $Na_3PS_4$ system.[66] The activation barrier computed for γ-$Li_{2.94}PS_4$ is significantly lower (372 meV) than for pristine γ-$Li_3PS_4$ (710 meV), clearly ratifying an enhanced Li mobility in the presence of vacancy defects. On the other hand, β-$Li_3PS_4$ has a higher Li conductivity than the γ-phase due to the partially occupied Li2 and Li3 crystal sites via a vacancy migration mechanism. Similarly, $Li_7P_3S_{11}$ has an open structure, which allows for the free movement of Li ions in the interstitial regions without the need for vacancies in the Li sublattice.[67,68]

Regarding the here presented new Li ion conductors, one can clearly note the enhanced Li ion conduction (accompanied by lower activation barriers) for the $Li_2S$-$Li_3P$ solid solutions compared to $Li_2S$. Along the solid solution increasing phosphorus content leads to lower activation energies. The constituents with highest $Li_3P$ contents, i.e. "$Li_8P_2S$" ($x$ = 0.67) and "$Li_{11}P_3S$" ($x$ = 0.75), with activation energies comparable to those of the known superionic conductors $Li_7P_3S_{11}$ and β-$Li_3PS_4$.

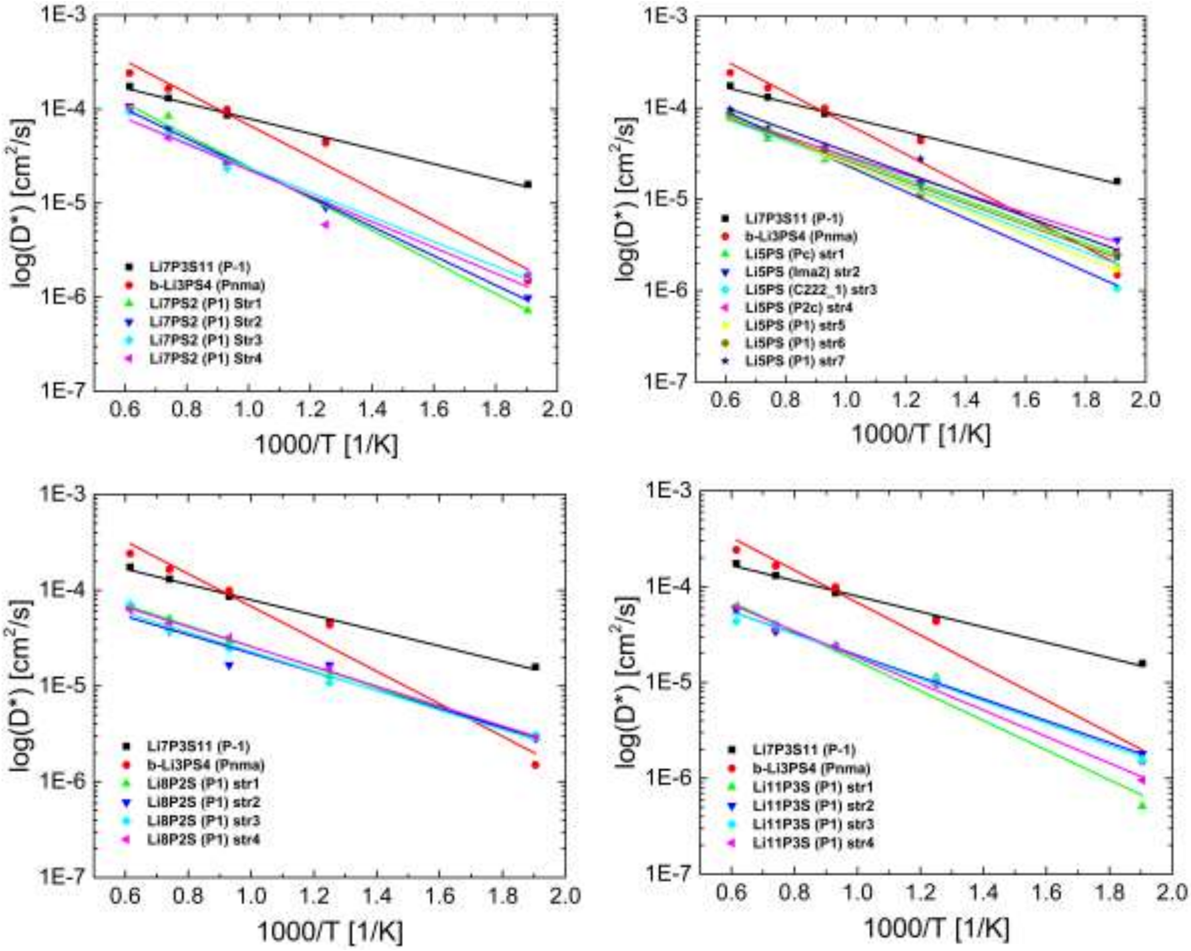


**Supporting Figure SB13.** Arrhenius plots for the different Li-P-S ternaries computed using *ab initio* molecular dynamics (NVT-type) simulations at varying temperatures. Top row gives the overall comparison for the ternaries and $Li_7P_3S_{11}$ with highest conductivity, other rows show the comparison of different configurations for each stoichiometry.

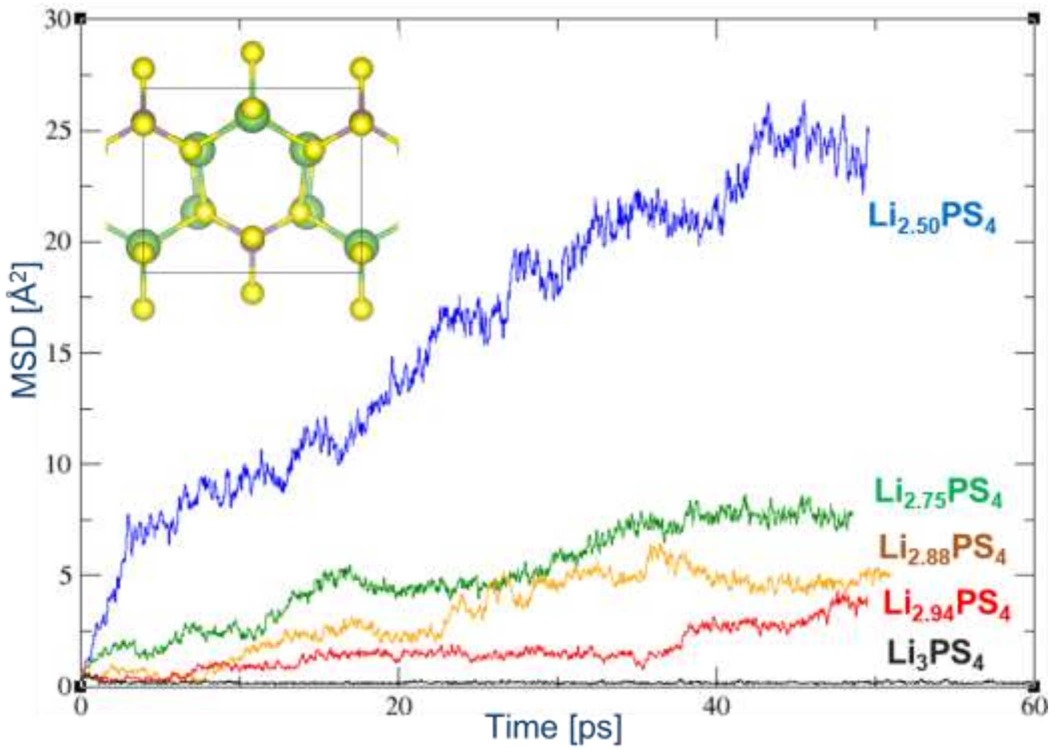


**Supporting Figure SB14.** Mean-square-displacements (MSD) for the Li ions in γ-$Li_{3-x}PS_4$, *x* = 0 - 0.5, from AIMD simulations at 525 K, showcasing the positive influence of vacancies in the Li sublattice on Li mobility.

## References


(1) Xu, Z.; Stebbins, J. F. 6Li Nuclear Magnetic Resonance Chemical Shifts, Coordination Number and Relaxation in Crystalline and Glassy Silicates. *Solid State Nuclear Magnetic Resonance* **1995**, *5* (1), 103–112. https://doi.org/10.1016/0926-2040(95)00026-M.

(2) Alam, T. M.; Conzone, S.; Brow, R. K.; Boyle, T. J. 6Li, 7Li Nuclear Magnetic Resonance Investigation of Lithium Coordination in Binary Phosphate Glasses. *Journal of Non-Crystalline Solids* **1999**, *258* (1), 140–154. https://doi.org/10.1016/S0022-3093(99)00481-0.

(3) Gamon, J.; Duff, B. B.; Dyer, M. S.; Collins, C.; Daniels, L. M.; Surta, T. W.; Sharp, P. M.; Gaultois, M. W.; Blanc, F.; Claridge, J. B.; Rosseinsky, M. J. Computationally Guided Discovery of the Sulfide $Li_3AlS_3$ in the Li–Al–S Phase Field: Structure and Lithium Conductivity. *Chem. Mater.* **2019**, *31* (23), 9699–9714. https://doi.org/10.1021/acs.chemmater.9b03230.

(4) Patel, M. U. M.; Arčon, I.; Aquilanti, G.; Stievano, L.; Mali, G.; Dominko, R. X-Ray Absorption Near-Edge Structure and Nuclear Magnetic Resonance Study of the Lithium–Sulfur Battery and Its Components. *ChemPhysChem* **2014**, *15* (5), 894–904. https://doi.org/10.1002/cphc.201300972.

(5) See, K. A.; Leskes, M.; Griffin, J. M.; Britto, S.; Matthews, P. D.; Emly, A.; Van der Ven, A.; Wright, D. S.; Morris, A. J.; Grey, C. P.; Seshadri, R. Ab Initio Structure Search and in Situ 7Li NMR Studies of Discharge Products in the Li–S Battery System. *J. Am. Chem. Soc.* **2014**, *136* (46), 16368–16377. https://doi.org/10.1021/ja508982p.

(6) Szczuka, Conrad. Investigation of the Ternary Li-P-S Phase Diagram for Application in Electrochemical Systems. Master Thesis, Master Thesis, RWTH Aachen University, Aachen, 2019.

(7) Jorcin, J.-B.; Orazem, M. E.; Pébère, N.; Tribollet, B. CPE Analysis by Local Electrochemical Impedance Spectroscopy. *Electrochimica Acta* **2006**, *51* (8), 1473–1479. https://doi.org/10.1016/j.electacta.2005.02.128.

(8) Irvine, J. T. S.; Sinclair, D. C.; West, A. R. Electroceramics: Characterization by Impedance Spectroscopy. *Advanced Materials* **1990**, *2* (3), 132–138. https://doi.org/10.1002/adma.19900020304.

(9) Bloembergen, N.; Purcell, E. M.; Pound, R. V. Relaxation Effects in Nuclear Magnetic Resonance Absorption. *Phys. Rev.* **1948**, *73* (7), 679–712. https://doi.org/10.1103/PhysRev.73.679.

(10) Abragam, A. *The Principles of Nuclear Magnetism*; Clarendon Press, 1961.

(11) Kelly, S. W.; Sholl, C. A. A Relationship between Nuclear Spin Relaxation in the Laboratory and Rotating Frames for Dipolar and Quadrupolar Relaxation. *J. Phys.: Condens. Matter* **1992**, *4* (12), 3317–3330. https://doi.org/10.1088/0953-8984/4/12/023.

(12) Kuhn, A.; Kunze, M.; Sreeraj, P.; Wiemhöfer, H.-D.; Thangadurai, V.; Wilkening, M.; Heitjans, P. NMR Relaxometry as a Versatile Tool to Study Li Ion Dynamics in Potential Battery Materials. *Solid State Nuclear Magnetic Resonance* **2012**, *42*, 2–8. https://doi.org/10.1016/j.ssnmr.2012.02.001.

(13) Heitjans, P.; Schirmer, A.; Indris, S. NMR and β-NMR Studies of Diffusion in Interface-Dominated and Disordered Solids. In *Diffusion in Condensed Matter: Methods, Materials, Models*; Heitjans, P., Kärger, J., Eds.; Springer: Berlin, Heidelberg, 2005; pp 367–415. https://doi.org/10.1007/3-540-30970-5_9.

(14) Indris, S.; Heitjans, P.; Uecker, R.; Roling, B. Li Ion Dynamics in a LiAlO2 Single Crystal Studied by 7Li NMR Spectroscopy and Conductivity Measurements. *J. Phys. Chem. C* **2012**, *116* (27), 14243–14247. https://doi.org/10.1021/jp3042928.

(15) Clark, S. J.; Segall, M. D.; Pickard, C. J.; Hasnip, P. J.; Probert, M. J.; Refson, K.; Payne, M. C. First Principles Methods Using {CASTEP}. *Z. Kristall.* **2005**, *220*, 567–570. https://doi.org/10.1524/zkri.220.5.567.65075.

(16) Segall, M.; Probert, M. First-Principles Simulation : Ideas , Illustrations and the CASTEP Code. **2002**, No. July 2015. https://doi.org/10.1088/0953-8984/14/11/301.

(17) Perdew, J. P.; Burke, K.; Ernzerhof, M. Generalized Gradient Approximation Made Simple. *Physical Review Letters* **1996**, *77* (18), 3865–3868. https://doi.org/10.1103/PhysRevLett.77.3865.
(18) Monkhorst, H. J.; Pack, J. D. Special Points for Brillouin-Zone Integrations. *Physical Review B* **1976**, *13* (12), 5188–5192. https://doi.org/10.1103/PhysRevB.13.5188.
(19) Liu, D. C.; Nocedal, J. On the Limited Memory BFGS Method for Large Scale Optimization. *Mathematical Programming* **1989**, *45* (1–3), 503–528. https://doi.org/10.1007/BF01589116.
(20) Nocedal, J. Updating Quasi-Newton Matrices with Limited Storage. *Mathematics of Computation* **1980**, *35* (151), 773–773. https://doi.org/10.1090/S0025-5718-1980-0572855-7.
(21) Maxwell, J. C. *The Scientific Papers of James Clerk Maxwell*; Niven, W. D., Ed.; Cambridge University Press: Cambridge, 2011. https://doi.org/10.1017/CBO9780511710377.
(22) Morris, A. J.; Grey, C. P.; Needs, R. J.; Pickard, C. J. Energetics of Hydrogen/Lithium Complexes in Silicon Analyzed Using the Maxwell Construction. *Physical Review B - Condensed Matter and Materials Physics* **2011**, *84* (22), 1–5. https://doi.org/10.1103/PhysRevB.84.224106.
(23) Morris, A. J.; Grey, C. P.; Pickard, C. J. Thermodynamically Stable Lithium Silicides and Germanides from Density Functional Theory Calculations. *Physical Review B* **2014**, *90* (5), 054111. https://doi.org/10.1103/PhysRevB.90.054111.
(24) Stratford, J. M.; Mayo, M.; Allan, P. K.; Pecher, O.; Borkiewicz, O. J.; Wiaderek, K. M.; Chapman, K. W.; Pickard, C. J.; Morris, A. J.; Grey, C. P. Investigating Sodium Storage Mechanisms in Tin Anodes: A Combined Pair Distribution Function Analysis, Density Functional Theory, and Solid-State NMR Approach. *Journal of the American Chemical Society* **2017**, *139* (21), 7273–7286. https://doi.org/10.1021/jacs.7b01398.
(25) Evans, M. MATADOR: An Aggregator, Manipulator and Runner of First-Principles Calculations, Http://Matador.Science/. 2019.
(26) Richards, W. D.; Hautier, G.; Ong, S. P.; Cholia, S.; Persson, K. A.; Jain, A.; Gunter, D.; Ceder, G.; Kocher, M.; Chevrier, V. L. Python Materials Genomics (Pymatgen): A Robust, Open-Source Python Library for Materials Analysis. *Computational Materials Science*. 2012, pp 314–319. https://doi.org/10.1016/j.commatsci.2012.10.028.
(27) Grau-Crespo, R.; Hamad, S.; Catlow, C. R. A.; De Leeuw, N. H. Symmetry-Adapted Configurational Modelling of Fractional Site Occupancy in Solids. *Journal of Physics Condensed Matter* **2007**, *19* (25), 0–16. https://doi.org/10.1088/0953-8984/19/25/256201.
(28) Pickard, C. J.; Mauri, F. All-Electron Magnetic Response with Pseudopotentials: NMR Chemical Shifts. **2001**, *63*, 1–13. https://doi.org/10.1103/PhysRevB.63.245101.
(29) Yates, J. R.; Pickard, C. J.; Mauri, F. Calculation of NMR Chemical Shifts for Extended Systems Using Ultrasoft Pseudopotentials. *Physical Review B - Condensed Matter and Materials Physics* **2007**, *76* (2), 1–11. https://doi.org/10.1103/PhysRevB.76.024401.
(30) Middlemiss, D. S.; Blanc, F.; Pickard, C. J.; Grey, C. P. Solid-State NMR Calculations for Metal Oxides and Gallates: Shielding and Quadrupolar Parameters for Perovskites and Related Phases. *Journal of Magnetic Resonance* **2010**, *204* (1), 1–10. https://doi.org/10.1016/j.jmr.2010.01.004.
(31) Zhang, X.; Tamaru, H.; Khan, S. I.; Horton, J. R.; Keefe, L. J.; Selker, E. U.; Cheng, X. Structure of the Neurospora SET Domain Protein DIM-5, a Histone H3 Lysine Methyltransferase. *Cell* **2002**, *111* (1), 117–127.
(32) Sturniolo, S. *Soprano — A Library to Crack Crystals, Https://Ccpforge.Cse. Rl.Ac.Uk/Gf/Project/Soprano/*.
(33) Friauf, R. J. Correlation Effects for Diffusion in Ionic Crystals. *Journal of Applied Physics* **1962**, *33* (1), 494–505. https://doi.org/10.1063/1.1777148.
(34) Kresse, G.; Hafner, J. Ab Initio Molecular Dynamics for Liquid Metals. *Physical Review B* **1993**, *47* (1), 558–561. https://doi.org/10.1103/PhysRevB.47.558.
(35) Kresse, G.; Furthmüller, J. Efficiency of Ab-Initio Total Energy Calculations for Metals and Semiconductors Using a Plane-Wave Basis Set. *Computational Materials Science* **1996**, *6* (1), 15–50. https://doi.org/10.1016/0927-0256(96)00008-0.

(36) Kresse, G. Efficient Iterative Schemes for Ab Initio Total-Energy Calculations Using a Plane-Wave Basis Set. *Physical Review B* **1996**, *54* (16), 11169–11186. https://doi.org/10.1103/PhysRevB.54.11169.
(37) Blöchl, P. E. Projector Augmented-Wave Method. *Physical Review B* **1994**, *50* (24), 17953–17979. https://doi.org/10.1103/PhysRevB.50.17953.
(38) Kresse, G. From Ultrasoft Pseudopotentials to the Projector Augmented-Wave Method. *Physical Review B* **1999**, *59* (3), 1758–1775. https://doi.org/10.1103/PhysRevB.59.1758.
(39) Perdew, J. P.; Yue, W. Accurate and Simple Density Functional for the Electronic Exchange Energy: Generalized Gradient Approximation. *Physical Review B* **1986**, *33* (12), 8800–8802. https://doi.org/10.1103/PhysRevB.33.8800.
(40) Karasulu, B.; Emge, S. P.; Groh, M. F.; Grey, C. P.; Morris, A. J. Al/Ga-Doped Li7La3Zr2O12 Garnets as Li-Ion Solid-State Battery Electrolytes: Atomistic Insights into Local Coordination Environments and Their Influence on 17O, 27Al, and 71Ga NMR Spectra. *J. Am. Chem. Soc.* **2020**, *142* (6), 3132–3148. https://doi.org/10.1021/jacs.9b12685.
(41) Pickard, C. J.; Needs, R. J. High-Pressure Phases of Silane. *Physical Review Letters* **2006**, *97* (4), 1–4. https://doi.org/10.1103/PhysRevLett.97.045504.
(42) Pickard, C. J.; Needs, R. J. Ab Initio Random Structure Searching. *Journal of Physics Condensed Matter* **2011**, *23* (5). https://doi.org/10.1088/0953-8984/23/5/053201.
(43) Bergerhoff, G.; Brown, I. D.; Allen, F.; others. Crystallographic Databases. *International Union of Crystallography, Chester* **1987**, *360*, 77–95.
(44) Jain, A.; Ong, S. P.; Hautier, G.; Chen, W.; Richards, W. D.; Dacek, S.; Cholia, S.; Gunter, D.; Skinner, D.; Ceder, G.; Persson, K. A. Commentary: The Materials Project: A Materials Genome Approach to Accelerating Materials Innovation. *APL Materials* **2013**, *1* (1), 011002. https://doi.org/10.1063/1.4812323.
(45) Saal, J. E.; Kirklin, S.; Aykol, M.; Meredig, B.; Wolverton, C. Materials Design and Discovery with High-Throughput Density Functional Theory: The Open Quantum Materials Database (OQMD). *JOM* **2013**, *65* (11), 1501–1509. https://doi.org/10.1007/s11837-013-0755-4.
(46) Dietrich, C.; Weber, D. A.; Culver, S.; Senyshyn, A.; Sedlmaier, S. J.; Indris, S.; Janek, J.; Zeier, W. G. Synthesis, Structural Characterization, and Lithium Ion Conductivity of the Lithium Thiophosphate Li2P2S6. *Inorg. Chem.* **2017**, *56* (11), 6681–6687. https://doi.org/10.1021/acs.inorgchem.7b00751.
(47) Wang, Y.; Richards, W. D.; Ong, S. P.; Miara, L. J.; Kim, J. C.; Mo, Y.; Ceder, G. Design Principles for Solid-State Lithium Superionic Conductors. *Nature Materials* **2015**, *14* (10), 1026–1031. https://doi.org/10.1038/nmat4369.
(48) Yamane, H.; Shibata, M.; Shimane, Y.; Junke, T.; Seino, Y.; Adams, S.; Minami, K.; Hayashi, A.; Tatsumisago, M. Crystal Structure of a Superionic Conductor, Li7P3S11. *Solid State Ionics* **2007**, *178* (15–18), 1163–1167. https://doi.org/10.1016/j.ssi.2007.05.020.
(49) Seino, Y.; Ota, T.; Takada, K.; Hayashi, A.; Tatsumisago, M. A Sulphide Lithium Super Ion Conductor Is Superior to Liquid Ion Conductors for Use in Rechargeable Batteries. *Energy Environ. Sci.* **2014**, *7* (2), 627–631. https://doi.org/10.1039/C3EE41655K.
(50) Homma, K.; Yonemura, M.; Kobayashi, T.; Nagao, M.; Hirayama, M.; Kanno, R. Crystal Structure and Phase Transitions of the Lithium Ionic Conductor Li3PS4. *Solid State Ionics* **2011**, *182* (1), 53–58. https://doi.org/10.1016/j.ssi.2010.10.001.
(51) Stöffler, H.; Zinkevich, T.; Yavuz, M.; Senyshyn, A.; Kulisch, J.; Hartmann, P.; Adermann, T.; Randau, S.; Richter, F. H.; Janek, J.; Indris, S.; Ehrenberg, H. Li + -Ion Dynamics in β-Li 3 PS 4 Observed by NMR: Local Hopping and Long-Range Transport. *The Journal of Physical Chemistry C* **2018**, *122* (28), 15954–15965. https://doi.org/10.1021/acs.jpcc.8b05431.
(52) Lin, Z.; Liu, Z.; Fu, W.; Dudney, N. J.; Liang, C. Lithium Polysulfidophosphates: A Family of Lithium-Conducting Sulfur-Rich Compounds for Lithium-Sulfur Batteries. *Angewandte Chemie International Edition* **2013**, *52* (29), 7460–7463. https://doi.org/10.1002/anie.201300680.
(53) Murayama, M.; Sonoyama, N.; Yamada, A.; Kanno, R. Material Design of New Lithium Ionic Conductor, Thio-LISICON, in the Li2S–P2S5 System. *Solid State Ionics* **2004**, *170* (3–4), 173–180. https://doi.org/10.1016/j.ssi.2004.02.025.

(54) Teragawa, S.; Aso, K.; Tadanaga, K.; Hayashi, A.; Tatsumisago, M. Liquid-Phase Synthesis of a Li3PS4 Solid Electrolyte Using N-Methylformamide for All-Solid-State Lithium Batteries. *Journal of Materials Chemistry A* **2014**, *2* (14), 5095. https://doi.org/10.1039/c3ta15090a.
(55) Teragawa, S.; Aso, K.; Tadanaga, K.; Hayashi, A.; Tatsumisago, M. Preparation of Li2S–P2S5 Solid Electrolyte from N-Methylformamide Solution and Application for All-Solid-State Lithium Battery. *Journal of Power Sources* **2014**, *248*, 939–942. https://doi.org/10.1016/j.jpowsour.2013.09.117.
(56) TACHEZ, M.; MALUGANI, J.; MERCIER, R.; ROBERT, G. Ionic Conductivity of and Phase Transition in Lithium Thiophosphate Li3PS4. *Solid State Ionics* **1984**, *14* (3), 181–185. https://doi.org/10.1016/0167-2738(84)90097-3.
(57) Mizuno, F.; Hayashi, A.; Tadanaga, K.; Tatsumisago, M. New, Highly Ion-Conductive Crystals Precipitated from Li2S-P2S5 Glasses. *Advanced Materials* **2005**, *17* (7), 918–921. https://doi.org/10.1002/adma.200401286.
(58) Moradabadi, A.; Kaghazchi, P. Thermodynamics and Kinetics of Defects in Li 2 S. *Applied Physics Letters* **2016**, *108* (21), 213906. https://doi.org/10.1063/1.4952434.
(59) Jand, S. P.; Zhang, Q.; Kaghazchi, P. Theoretical Study of Superionic Phase Transition in Li 2 S. *Scientific Reports* **2017**, No. June, 2–7. https://doi.org/10.1038/s41598-017-05775-2.
(60) Lin, Z.; Liu, Z.; Dudney, N. J.; Liang, C. Lithium Superionic Sulfide Cathode for All-Solid Lithium–Sulfur Batteries. *ACS Nano* **2013**, *7* (3), 2829–2833. https://doi.org/10.1021/nn400391h.
(61) Lorger, S.; Narita, K.; Usiskin, R.; Maier, J. Enhanced Ion Transport in Li2O and Li2S Films. *Chem. Commun.* **2021**, *57* (53), 6503–6506. https://doi.org/10.1039/D1CC00557J.
(62) Altorfer, F.; Bührer, W.; Anderson, I.; Schärpf, O.; Bill, H.; Carron, P. L.; Smith, H. G. Lithium Diffusion in the Superionic Conductor Li2S. *Physica B: Condensed Matter* **1992**, *180–181* (92), 795–797. https://doi.org/10.1016/0921-4526(92)90471-4.
(63) Nazri, G. Preparation, Structure and Ionic Conductivity of Lithium Phosphide. *Solid State Ionics* **1989**, *34* (1–2), 97–102. https://doi.org/10.1016/0167-2738(89)90438-4.
(64) Wegner, F.; Kamm, F.; Pielnhofer, F.; Pfitzner, A. $Li_3$ As and $Li_3$ P Revisited: DFT Modelling on Phase Stability and Ion Conductivity. *Zeitschrift anorg allge chemie* **2022**. https://doi.org/10.1002/zaac.202100358.
(65) Klerk, N. J. J. De; Wagemaker, M. Diffusion Mechanism of the Sodium-Ion Solid Electrolyte Na 3 PS 4 and Potential Improvements of Halogen Doping-SI. *Chemistry of Materials* **2016**, *28* (9), 1–4. https://doi.org/10.1021/acs.chemmater.6b00698.
(66) de Klerk, N. J. J.; Wagemaker, M. Diffusion Mechanism of the Sodium-Ion Solid Electrolyte Na3PS4 and Potential Improvements of Halogen Doping. *Chem. Mater.* **2016**, *28* (9), 3122–3130. https://doi.org/10.1021/acs.chemmater.6b00698.
(67) Xiong, K.; Longo, R. C.; Kc, S.; Wang, W.; Cho, K. Behavior of Li Defects in Solid Electrolyte Lithium Thiophosphate Li7P3S11: A First Principles Study. *Computational Materials Science* **2014**, *90*, 44–49. https://doi.org/10.1016/j.commatsci.2014.03.030.
(68) Yamane, H.; Shibata, M.; Shimane, Y.; Junke, T.; Seino, Y.; Adams, S.; Minami, K.; Hayashi, A.; Tatsumisago, M. Crystal Structure of a Superionic Conductor, Li7P3S11. *Solid State Ionics* **2007**, *178* (15), 1163–1167. https://doi.org/10.1016/j.ssi.2007.05.020.
